# Comparative genomics suggests loss of keratin K24 in three evolutionary lineages of mammals

**DOI:** 10.1038/s41598-019-47422-y

**Published:** 2019-07-29

**Authors:** Florian Ehrlich, Maria Laggner, Lutz Langbein, Pamela Burger, Andreas Pollreisz, Erwin Tschachler, Leopold Eckhart

**Affiliations:** 1 Research Division of Biology and Pathobiology of the Skin, Department of Dermatology, Medical University of Vienna, Vienna, Austria; 2 Department of Ophthalmology, Medical University of Vienna, Vienna, Austria; 3 German Cancer Research Center, Department of Genetics of Skin Carcinogenesis, Heidelberg, Germany; 4 Research Institute of Wildlife Ecology, University of Veterinary Medicine Vienna, Vienna, Austria; 5 Present Address: Aposcience AG, Vienna, Austria

**Keywords:** Molecular evolution, Gene expression

## Abstract

Keratins are the main cytoskeletal proteins of epithelial cells and changes in the expression of keratins have contributed to the evolutionary adaptation of epithelia to different environments. Keratin K24 was proposed to be a differentiation marker of epidermal keratinocytes but the significance of K24 expression in the epidermis versus other tissues has remained elusive. Here, we show by RT-PCR, western blot, and immunofluorescence analyses that K24 is highly expressed in the epithelium of the cornea whereas its expression levels are significantly lower in other stratified epithelia including in the epidermis. To investigate the evolutionary history of K24, we screened the genome sequences of vertebrates for orthologs of the human *KRT24* gene. The results of this comparative genomics study suggested that *KRT24* originated in a common ancestor of amniotes and that it was lost independently in three clades of mammals, i.e. camels, cetaceans, and a subclade of pinnipeds comprising eared seals and the walrus. Together, the results of this study identify K24 as component of the cytoskeleton in the human corneal epithelium and reveal previously unknown differences of keratin gene content among mammalian species.

## Introduction

Keratins are intermediate filament (IF) proteins that form the cytoskeleton of epithelial cells^1–4^. Human epithelia contain 28 type I and 26 type II keratins that have a conserved central α-helical domain but differ in several molecular properties such as the presence or absence of glycine and serine-rich sequences in the amino- and carboxy-terminal domains^5^. The 54 keratin genes in the human genome have evolved from a common precursor and acquired specific transcription-regulatory elements during their diversification^6–8^. Accordingly, all type I and all type II keratins have unique expression patterns in the various epithelia of the body whereby many, but not all, type I keratins are co-expressed with a specific type II keratin^9–14^. For instance, keratins K5 and K14 are co-expressed in the basal layer of stratified epithelia, and K1 and K10 are co-expressed in the suprabasal layers of the epidermis to form heterodimers. However, K2 is also expressed in human suprabasal epidermis^15^ so that the presence of both K1:K10 and K2:K10 dimers in the same cells causes complexity in the cytoskeleton of differentiated keratinocytes.

**Figure 1:**
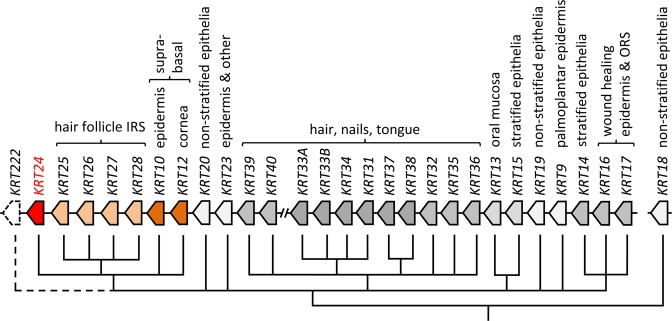
Loci and phylogenetic relationships human type I keratin genes. The schematic shows the loci of *KRT24* and other human type I keratin genes and the phylogenetic relationships of the genes^27^. Branches without significant bootstrap support are collapsed. Arrows indicate the genes with arrow tips pointing in the direction of gene transcription. The expression pattern of each keratin in human tissues is indicated above the genes. With the exception of *KRT18* (chromosome 12q13), all type I keratin genes are clustered on chromosome 17q21. *KRTAP* genes, located between *KRT40* and *KRT33A*, are not shown. Note that *KRT222* (indicated by an arrow drawn with a broken line) encodes a keratin-like protein of unknown function. IRS, inner root sheath; ORS, outer root sheath.

Keratin K24 is encoded by the *KRT24* gene that is located at one end of the type I keratin gene cluster where it is flanked by *KRT222*, which encodes an as-yet uncharacterized keratin-like protein, and *KRT25*^16–18^. *KRT24* and *KRT25* belong to a type I keratin gene subcluster that is characterized by expression in differentiated cells within the suprabasal layers of stratified epithelia or epithelial appendages (Fig. 1). The other members of this subcluster are *KRT26*, *KRT27*, and *KRT28*, which like *KRT25* are expressed in the inner root sheath (IRS) of hair follicles^19^, *KRT10* which is expressed in the epidermis, and *KRT12* which is expressed in the epithelium of the cornea. Recently, K24 was reported, based on immunodetection, to be expressed in the suprabasal layers of human epidermis and in epidermal *in vitro* models^20,21^. Another article reported expression of K24 mRNA in the corneal epithelium^22^.

In the present study, we compared the expression levels of *KRT24* in the epithelia of the cornea and the skin and investigated the *KRT24* gene locus in phylogenetically diverse amniotes. We provide evidence for a predominant expression of human *KRT24* in differentiated corneal epithelial cells and for differential conservation and loss of *KRT24* orthologs among mammals.

## Results

### *KRT24* is expressed at high levels in the human cornea

**Figure 2:**
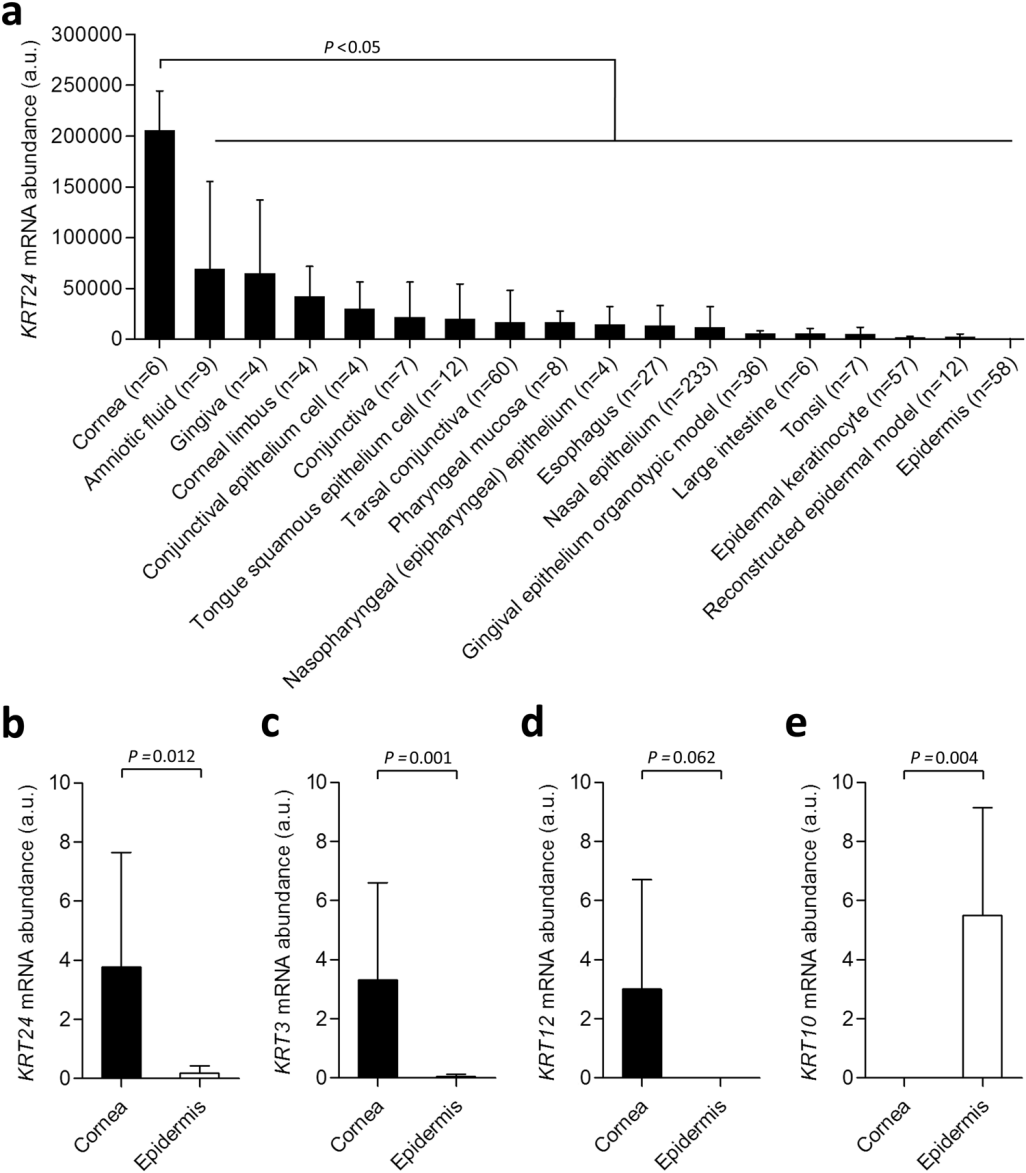
*KRT24* mRNA levels are high in the cornea. (**a**) *KRT24* mRNA levels in human tissues and cell types were extracted from the Genevestigator database. Expression values in arbitrary units (a.u.) according to Genevestigator^23^ are shown. Bars indicate means and error bars show standard deviations. The number (n) of samples per tissue or cell type is indicated below the bars. *P*-values were calculated with the two-sided t-test. (**b**–**e**) Quantitative RT-PCR analysis of mRNAs of selected keratin genes in human cornea (n = 8) and epidermis (n = 5). Expression levels of *KRT24* (**b**), *KRT3* (**c**), *KRT12* (**d**), and *KRT10* (**e**) were calculated relative to that of GAPDH and normalized to the highest value (defined as 10 arbitrary units, a.u.) among the samples investigated. Bars indicate means and error bars show standard deviations. *P*-values (Mann-Whitney U Test) are shown above the graphs.

The expression levels of *KRT24* in human tissues and cell types were analyzed in the Genevestigator database, a comprehensive collection of public microarray and RNA-Seq study results^23^. The highest levels of *KRT24* mRNA were found in the cornea, followed by the amniotic fluid, conjunctiva, oral epithelia, and the esophagus (Fig. 2a). Only very low amounts of *KRT24* mRNA were found in the epidermis (Fig. 2a), although previous reports had suggested a role of *KRT24* in differentiation of epidermal keratinocytes.

Next, we compared *KRT24* mRNA amounts in cornea and epidermis by quantitative RT-PCR analysis. In agreement with the Genevestigator database (Fig. 2a), mRNA levels of *KRT24* were significantly higher in the cornea than in the epidermis (Fig. 2b). The expression of *KRT24* resembled that of *KRT3* (Fig. 2c) and *KRT12* (Fig. 2d), i.e. the established marker keratins of the differentiated corneal epithelium^24,25^, and was opposite to the expression of *KRT10*, a marker of epidermal differentiation (Fig. 2e).

### K24 protein is present in the superficial cells of the corneal epithelium

**Figure 3:**
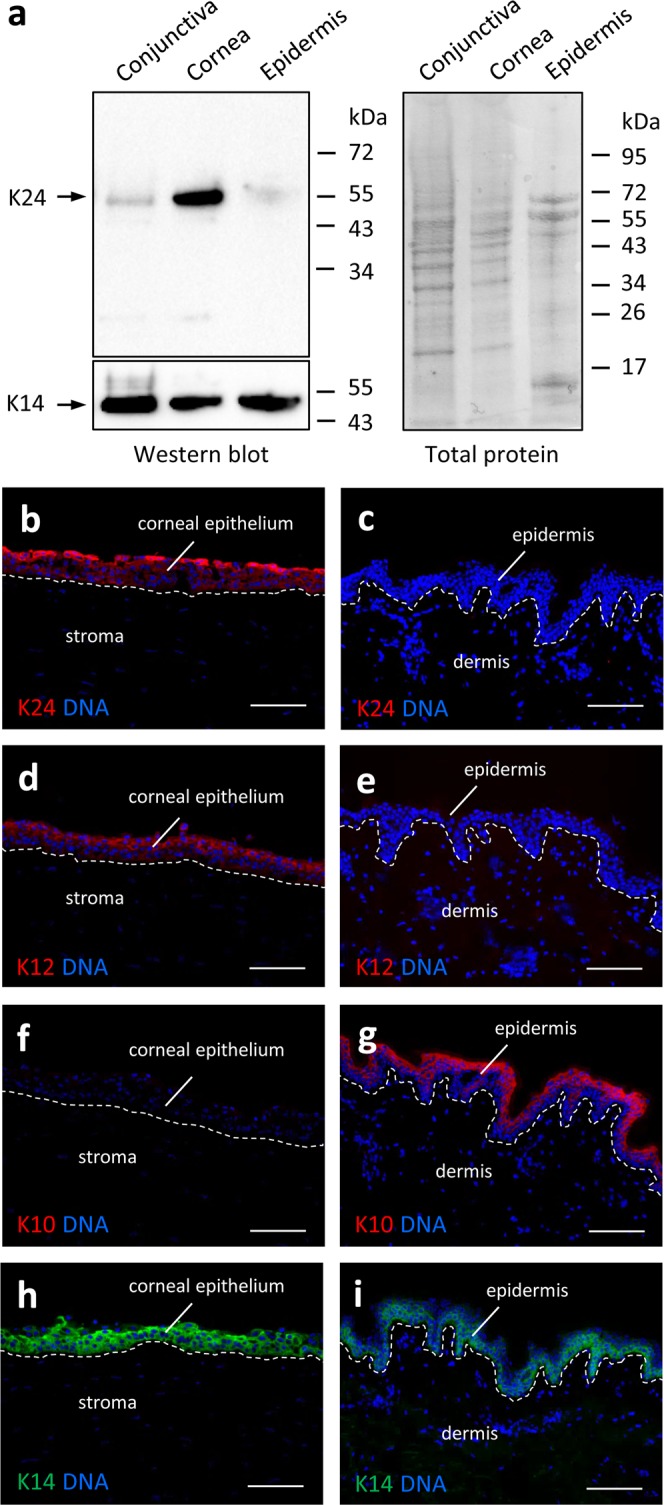
Immunodetection of K24 protein in the human cornea. (**a**) Western blot analysis of K24 and K14 in human conjunctiva, cornea and epidermis. The total protein blotted onto the membrane was visualized by Ponceau staining prior to incubation with blocking buffer and probing with antibodies. Results are representative for n ≥ 3 samples of each tissue. Positions of size markers are shown on the right. kDa, kilo-Dalton. (**b**) Immunofluorescence labeling of K24, K12, K10 (red) and K14 (green) in human cornea and skin. DNA was labeled with Hoechst 33258 dye (blue). The junctions between epithelia and mesenchymal tissues are indicated by broken lines. Results are representative for at least 3 samples of each tissue. Scale bars: 100 µm.

To determine the distribution of K24 protein in human tissues, we generated an antibody against an epitope within the tail domain of K24. In western blot analysis, this antibody detected a band at the expected size of 55 kD (Fig. 3a). Only low amounts of K24 were present in the epidermis and conjunctiva, whereas a prominent band of K24 was detected in lysates obtained from human corneas (Fig. 3a).

Immunofluorescence analysis revealed expression of K24 in the superficial layer of the corneal epithelium (Fig. 3b) whereas no significant labeling was detected in the epidermis (Fig. 3c). K12 showed a similar distribution but was also expressed in lower suprabasal corneal epithelial cells (Fig. 3d,e). K10 was absent in the cornea and abundant in the epidermis (Fig. 3e,f). K14 was expressed in the basal layer of both corneal epithelium and epidermis (Fig. 3h,i). Thus, *KRT24* mRNA and K24 protein distribution patterns were highly consistent and provided solid evidence for predominant expression of *KRT24* in the corneal epithelium.

Besides the cornea of the eye and the skin, we performed immunofluorescence analysis of the esophagus, representing one of the organs that contain low but significant levels *KRT24* mRNA (Fig. 2)^26^. K24 was detected in suprabasal epithelial cells of the esophagus with immunofluorescence signal intensities weaker than in the cornea (Suppl. Fig. S1).

### K24 is an evolutionary ancient keratin that has been differentially conserved or lost in different clades of mammals

**Figure 4:**
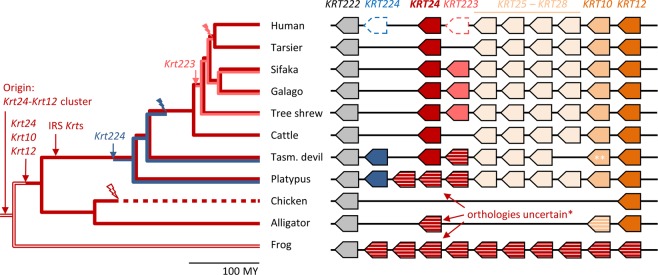
Evolution of the *KRT24* gene locus. A model for the evolutionary history of *KRT24* and neighboring keratin genes was developed from the comparison of corresponding gene loci in tetrapods. Genes are indicated by arrows that point from the 5′-end to the 3′-end of the coding sequence. A timed phylogenetic tree^46^ is depicted besides the gene loci. Gene gain and loss events were inferred by the principle of maximum parsimony from the species distribution of the genes. The presence or absence of *KRT24*, *KRT223*, and *KRT224* genes in ancestors of modern species is indicated by lines of the same color as the corresponding gene in the schematic on the right. The scale bar below the phylogenetic tree indicates a period of 100 million years (MY). Note that *KRT223* and *KRT224* are pseudogenes in the human genome and that orthology relationships could not be faithfully resolved by molecular phylogenetics for *Krt24*-like genes of several species (*). Two copies of the *Krt10* gene are present in marsupials but only one copy is shown in this schematic (**). IRS *Krt*s, inner root sheath keratin genes (*KRT25-28*).

Epithelia of the body surface are subjected to environment-dependent evolutionary constraints that affect their molecular composition, including proteins of the cytoskeleton. Therefore, we hypothesized that the locus of the *KRT24* gene might have undergone differential evolution in species with different lifestyles. To test this hypothesis, we performed a comparative genomics study and determined the presence or absence of *KRT24* orthologs in phylogenetically diverse vertebrates. The investigation was focused on the locus bordered by *KRT222* and *KRT12* (Fig. 4). In line with results of previous studies^27,28^, we identified *Krt24*-like genes but not 1:1 orthologs of *Krt24* besides *Krt222* in fish and amphibians. In the alligator a *Krt24*-like gene is located between *Krt222* and *Krt10*, and three *Krt24*-like genes are present in the platypus, representing basal mammals (Supplementary Tables S1–S7; Suppl. Fig. S2; Fig. 4). By contrast, no *Krt24* homolog is present in the chicken (Fig. 4). *Krt24* orthologs are conserved in the majority of mammals with a few notable exceptions that will be discussed below.

Interestingly, we found diversity in the genes immediately flanking *Krt24* in different mammals. *Krt223* is located on the 5′-side of *Krt24* in primates of the suborder Strepsirrhini (galago and sifaka) and the tree shrew whereas only a remnant of this gene (*KRT223P*, keratin 223 pseudogene) is present in the human genome (Fig. 4). Similarly, *Krt224* is located on the 3′-side of *Krt24* in the platypus and marsupials (Tasmanian devil) but not in the placental mammals investigated, such as human and other primates, tree shrew, and cattle (Fig. 4). A pseudogene corresponding to *Krt224* was identified in the human genome (Suppl. Fig. S3). By mapping these genes and pseudogenes onto the phylogenetic tree of mammals (Fig. 4), we inferred that the evolution of the human set of type I keratins involved the loss of at least two genes, i.e. *Krt223* and *Krt224*, that were retained in some other extant mammals.

**Figure 5:**
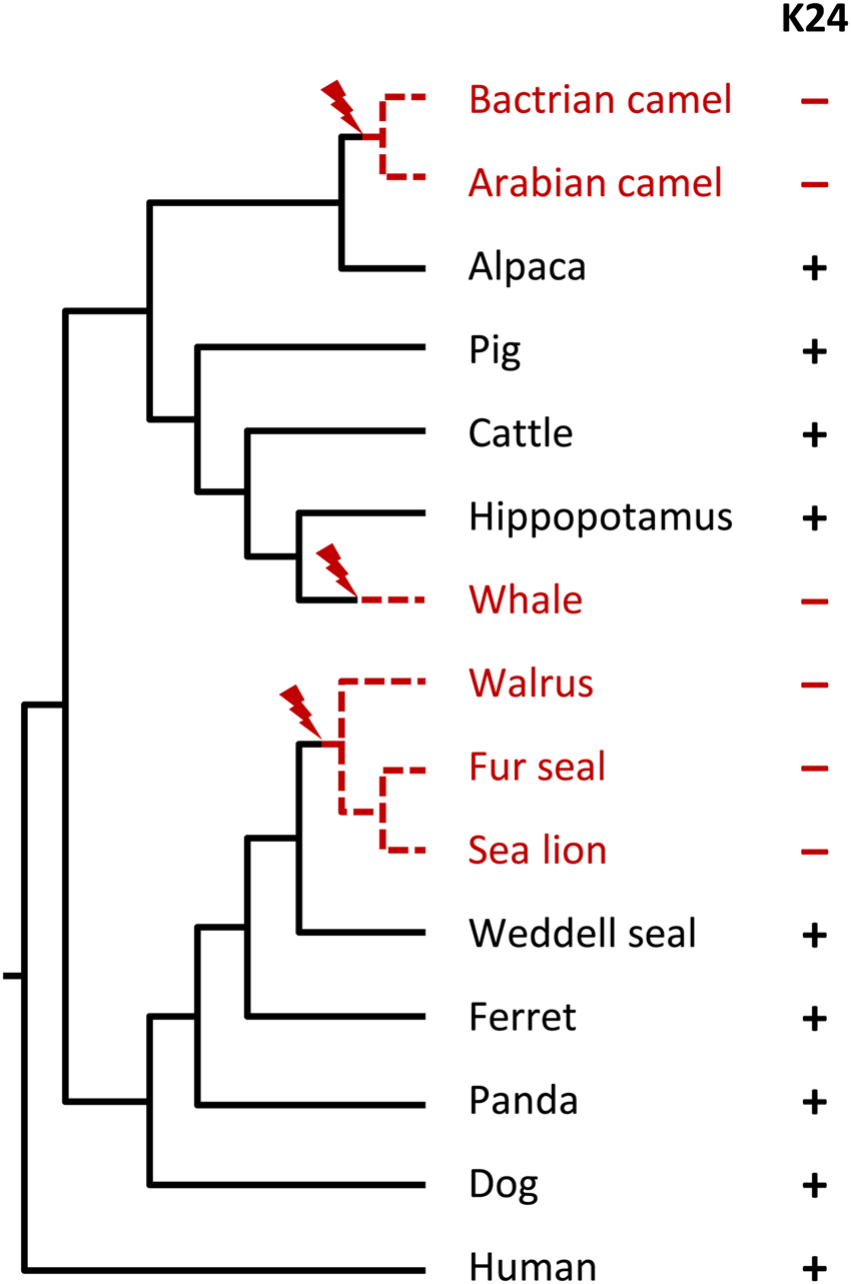
Loss of *Krt24* in different clades of mammals. The presence (+) or absence (−) of functional *Krt24* genes was mapped onto a phylogenetic tree of mammals. Only *Krt24*-deficient species and close relatives are shown.

The analysis of publicly available genome sequences showed that *Krt24* was conserved in most mammals but not in cetaceans^18^, a subclade of pinnipeds, and camels (Fig. 5, Suppl. Fig. S4). *Krt24* was lost in cetaceans after their evolutionary divergence from the lineage leading to their next extant relative, the hippopotamus (Suppl. Fig. S4). *Krt24* gene remnants were found in the genome of several cetaceans, including the bottlenose dolphin (*Tursiops truncatus*). Amplification and sequencing confirmed that the open reading frame of *Krt24* is disrupted in the dolphin (Suppl. Fig. S5). Within pinnipeds, the walrus (*Odobenus rosmarus divergens*), the northern fur seal (*Callorhinus ursinus*) and the California sea lion (*Zalophus californianus*), together representing the clade Otaroidea, lack a functional *Krt24* gene (Suppl. Fig. S4). Within the family Camelidae, *Krt24* has been inactivated in the genus Camelus including *C*. *dromedarius*, *C*. *bactrianus*, and *C*. *ferus*, whereas *Krt24* is intact in the alpaca (*Vicugna pacos*) (Suppl. Fig. S5). These cases of gene loss indicate that *Krt24* had played a redundant or even disadvantageous role in ancestors of several extant mammals. It will be interesting to determine whether loss of *Krt24* correlates with adaptions of the corneal epithelium or other epithelia in these species.

**Figure 6:**
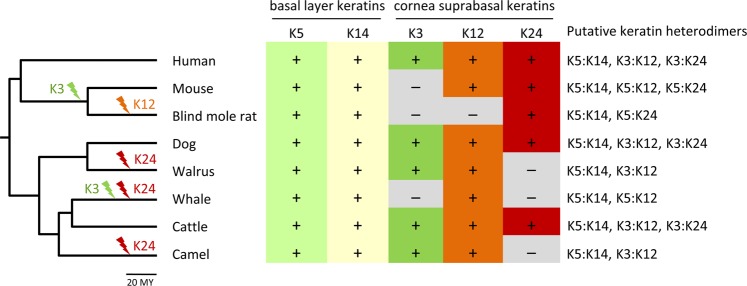
Evolution of corneal epithelial keratins in mammals. The presence (+) or absence (−) of functional orthologs of human corneal epithelial keratins K5, K14, K3, K12, and K24 was mapped onto a phylogenetic tree of mammals. Gene loss events were inferred from the distribution of functional genes and known relationships of species. Putative keratin heterodimers in the cornea are listed for each species. The scale bar beneath the timed phylogenetic tree indicates a period of 20 million years (MY).

Finally, we compared the pattern of conservation and loss of K24 with that of other keratins. Neither did the pattern of K24 conservation among species correlate with that of any other keratin nor did the corneal keratins K3 and K12 show strict co-evolution (Fig. 6). Thus, the functions of each K24, K12, and K3 can be replaced by other keratins or have become dispensable in distinct evolutionary lineages of mammals.

## Discussion

The results of this study provide important new insights into the expression pattern and evolution of the *KRT24* gene. *KRT24* is predominantly expressed in the corneal epithelium and, within this epithelium, in terminally differentiated cells on the surface. With regard to this epithelial cell differentiation-associated expression, *KRT24* resembles the other members of the type I keratin subcluster also comprising suprabasal epidermal keratin *KRT10*, suprabasal corneal keratin *KRT12*, and the IRS keratins *KRT25*, *KRT26*, *KRT27*, and *KRT28* (Fig. 1). It is therefore likely that a suprabasally expressed type I keratin gene was the molecular ancestor of all these genes including *KRT24* and that duplications and modifications of promoter and coding sequences have led to the diversification and specification of expression patterns and protein properties.

*KRT24* differs from the other members of the suprabasal type I keratin gene cluster because it has a relatively broad tissue expression pattern (Fig. 2a). While *KRT10*, *KRT12*, and the IRS keratins are hardly expressed outside of their predominant sites, i.e., the suprabasal epidermis, the corneal epithelium and the differentiated keratinocytes of the IRS, respectively, *KRT24* mRNA levels are significant in the cornea, oral epithelium, and esophagus. This tissue distribution indicates that K24 contributes to the cytoskeleton of stratified squamous epithelia that, unlike the epidermis, do not form a cornified layer on the surface. It is also interesting to note that all K24-positive epithelia contain at least one further suprabasal type I keratin and K24 is probably a quantitatively minor keratin at all these sites.

Type I keratin K24 is coexpressed with different type II keratins in different epithelia. At its predominant expression site, the suprabasal corneal epithelium, K3 is the main type II keratin and therefore the prime candidate for heterodimerization with K24. In other human epithelia and in the corneal epithelium of the mouse^29^, K3 is missing whereas other type II keratins such as K4 and K5 are abundant. Therefore, K24 appears to be a promiscuous heterodimerization partner of type II keratins, however, coexpression experiments and ultrastructural localization studies will be necessary to test this hypothesis. The distribution of K24 in the corneal epithelium differs from that of K3 and K5 because K24 is confined to the most superficial cells. This distribution and our demonstration of absence of K24 in several species suggest that K24 is not indispensable for the formation of a cytoskeleton in corneal epithelial cells. Similar to the start of expression of K2, besides expression of K1 and K10, in terminally differentiating keratinocytes of the epidermis^15^, the increase of expression of K24 in terminally differentiating cells is likely to add new components to the cytoskeleton, thereby modifying its properties. Targeted deletion of the *Krt24* gene in mice will help to define the roles of K24 in the corneal epithelium in future studies.

Importantly, our data indicate that expression of *KRT24* in the epidermis, as it was reported recently^20^, is smaller by almost 2 orders of magnitude than that in the cornea. Accordingly, we recommend K24 not to be considered an epidermal differentiation marker. Our side-by-side comparison of cornea and epidermis by RT-PCR, western blot and immunofluorescence analysis showed consistently that mRNA and protein levels of K24 in the epidermis were very low as compared to the cornea. The identity of *KRT24* RT-PCR products was confirmed by sequencing, K24 protein was only detected in the tissue that contained high amounts of *KRT24* mRNA, and our anti-K24 antibody yielded only a single western blot band at the expected size of K24. Another anti-K24 antibody (ab180486) that was reported to yield strong signals in the granular and cornified layers of the epidermis^20,21^ was not distributed by the manufacturer when the present manuscript was written (March 2019). Yet another antibody (HPA022978) that was tested in the Human Protein Atlas project yielded staining signals in several tissues that do not contain *KRT24* mRNA^26^. The reasons for the discrepancies in the immunoreactivities of different antibodies is not known but likely involves cross-reactivities with other antigens. Based on our RT-PCR, western blot and immunofluorescence data, we can conclude that the abundance of K24 in the epidermis, if it is present there, is considerably lower than in the cornea.

*KRT24* originated in amphibious or terrestrial vertebrates and was conserved in most but not all amniotes. It is presently unknown whether *Krt24* is expressed in the corneal epithelium of species other than human. The expressed sequence tag (EST) profile of murine *Krt24* in GenBank (https://www.ncbi.nlm.nih.gov/UniGene/ESTProfileViewer.cgi?uglist=Mm.46378, last accessed 11 March 2019), shows expression in “eye” besides “tongue” and “embryonic tissue”, indicating that conserved expression of *Krt24* in the cornea is likely. A quantitative comparison of K24 expression in the cornea and the tongue was not possible in this study because tongue samples were not available. However, *KRT24* mRNA is also present in the esophagus (Fig. 2a) and immunolabeling of formalin-fixed and paraffin-embedded esophagus confirmed expression of K24, though at lower levels than in the cornea (Suppl. Fig. S1). These data suggest that *Krt24* has additional roles in extra-corneal tissues of both mice and humans. In line with the notion of *Krt24* function(s) outside of the cornea, the blind mole rat (*Spalax galili*) has retained an intact *Krt24* gene (Fig. 6) despite degeneration of the corneal epithelium during the evolution of this species^30,31^.

Although *Krt24* has been conserved in phylogenetically diverse mammals, we have also found evidence for loss of *Krt24* in three clades of mammals, i.e. cetaceans, Otaroidea and camels. The premature stop codons identified in the *Krt24* genes of these mammals are predicted to cause nonsense-mediated decay of mRNA^32^ as they are located before the last exon. Even if mRNA is translated, the protein would lack a part or the complete intermediate filament domain which is required for the function of K24 as a component of the cytoskeleton. Mutations in camels and a representative cetacean were confirmed by our sequencing of genomic DNA. A premature stop in *Krt24* genes of walrus and sea lion was located at a conserved position, suggesting that this stop codon was inherited from a common ancestor. It is noteworthy that a *Krt24*-like gene is present in the alligator but not in birds (Fig. 4), possibly indicating loss of this gene during the evolution of birds. In mammals, *Krt24* is flanked by the genes *Krt25* through *Krt28*, which have originated after the divergence of mammals from reptiles. *Krt25* through *Krt28* are expressed specifically in hair follicles and were lost when hair follicles degenerated during the evolution of cetaceans^18^.

The identified cases of evolutionary loss of *Krt24* indicate that this gene is dispensable in three clades of mammals. Furthermore, these data raise the question as to whether loss of *Krt24* was associated with phenotypic changes. Cetaceans have many adaptations to aquatic life that include thickening of the epidermis^18,33,34^ and perhaps also changes in the cornea and internal epithelia. In the present study we show that the next relative of cetaceans, i.e. the hippopotamus does not have inactivating mutations in *Krt24*, whereas other largely aquatic mammals, i.e. members of a subclade (eared seals + walrus) of pinnipeds, lack functional *Krt24*. Yet other aquatic mammals such as sirenians and earless seals (Phocidae) have intact *Krt24* genes, suggesting that loss of *Krt24* is not an obligatory adaptation to aquatic life. Interestingly, *Krt24* was inactivated in camels but not in their next relative, the alpaca. Camels have a thicker corneal epithelium than most or all other mammals^35^, possibly representing an adaptation to the low humidity of their environment or to mechanical abrasion of superficial cells during exposure to sand and dust particles. It will be interesting to determine links between loss of *Krt24* and specific features of epithelia in the aforementioned species.

Taken together, *Krt24* has a complex evolutionary history in amniotes and its expression pattern in humans suggests that it contributes to the keratin cytoskeleton of several stratified epithelia. High levels of K24 expression in the superficial layer of the corneal epithelium point to a predominant role of human K24 in the cornea. Accordingly, possible aberrations of K24 function should be tested in future studies using samples from normal and diseased corneas displaying impaired differentiation.

## Materials and Methods

### Human tissues

The cornea bank at the Medical University of Vienna procured postmortem human corneal tissues (ethics approval number EK1578/2013). Human skin was obtained from plastic surgery (ethics approval number EK2011/1149). All donors provided written informed consent.

### Comparative genomics

The genome sequences of frog (*Xenopus tropicalis*)^36^, Chinese alligator (*Alligator sinensis*)^37^, platypus (*Ornithorhynchus anatinus*)^38^, Tasmanian devil (*Sarcophilus harrisii*)^39^, cattle (*Bos taurus*), tree shrew (*Tupaia chinensis*)^40^, mouse (*Mus musculus*), galago (*Otolemur garnetti*), sifaka (*Propithecus coquereli*), tarsier (*Carlito syrichta*), and human (*Homo sapiens*) were screened for the presence or absence and sequence integrity of *Krt24* and neighbouring genes. In addition, corneal epithelium-specific keratin genes *Krt3* and *Krt12* were screened with the same criteria in blind mole rate (*Spalax galili*)^30^, sperm whale (*Physeter catodon*), dolphin (*T*. *truncatus*) and camels (*Camelus dromedarius* and *Camelus bactrianus*). The genome sequences of other species were used for sequence comparisons. The sequences were retrieved from the GenBank database of the National Center for Biotechnology Information (NCBI), USA (http://www.ncbi.nlm.nih.gov/). Local sequence similarity was obtained using the Basic Local Alignment Search Tool (BLAST)^41^. The conservation of blocks of order of genetic elements (synteny) was tested by manual alignment of gene maps including conserved genes on each side of the gene(s) of interest. Gene orthologies between species were inferred from shared synteny and phylogenetic analysis.

### Sequence alignments and phylogenetic analysis

Alignments were performed with Multiple Sequence Comparison by Log-Expectation (MUSCLE)^42^, only the unambiguously aligned intermediate filament structures of the keratins were used for subsequent phylogenetic analyses. Maximum likelihood estimation was reconstructed using the Seaview platform^43^, using a JTT model with 100 bootstrap replicates to assess clade support. A mixed amino acid model with gamma correction for rate heterogeneity and invariable sites was used for estimating Bayesian posterior probabilities in MrBayes 3.2.6^44^. Two parallel Markov chain Monte Carlo (MCMC) runs of four chains each were performed, with a length of 3,500,000 generations, a sampling frequency of 1 per 1,000 generations. To define the burnin and check the convergence of the MCMC runs, Tracer v1.7.1 was used^45^. Trees were visualized with FigTree v1.4.2. Phylogenetic relationships and divergence times were obtained from the Timetree website (www.timetree.org)^46^.

### Sequence analysis of Krt24 genes

DNA from two camel species (*Camelus dromedarius* and *Camelus bactrianus*)^47^, transferred from the Austrian Science Fund project P1084-B17 (P. Burger), was investigated by PCRs using the following primers annealing to the *Krt24* gene of both *C*. *dromedarius* (GeneID:105031793) and *C*. *bactrianus* (GeneID:105073329): camel-K24-s (5′-TCCCCTCCTTCAGTGTCCTA-3′) and camel-K24-a (5′-GAAGCAGCTTGTGTGTGACC-3′). Twenty-five ng template DNA were used for PCR, involving an annealing temperature of 59 °C and 38 cycles. DNA of bottlenose dolphin (*T*. *truncatus*)^31^ was subjected to PCR amplification with the primer pair dolphin-K24-s (5′-CCCCACGTCCATTCTATGACAGT-3′) and dolphin-K24-a (5′-TGATGAGATGAGCGGAAGTGGC-3′) using an annealing temperature of 62 °C and 35 cycles. Sequencing of PCR products was conducted by Microsynth AG, 6961 Wolfurt, Austria.

### Quantification of mRNAs in tissues

The Genevestigator database^23^ was used to determine mRNA levels of *KRT24* in human tissues and cell types. The expression values in Genevestigator are calculated using standard normalization methods for different microarray platforms and scaled between experiments to make the expression values comparable^23^.

For quantititative reverse transcription-polymerase chain reaction (RT-PCR), RNA was purified from human cornea and epidermis using the Precellys system (VWR International, Radnor, PA) and TriFast (VWR International) according to the manufacturers’ instructions. 500 ng RNA were reverse-transcribed to cDNA using the Iscript^TM^ Kit (Biorad, Hercules, CA). Quantitative PCRs were performed using the LightCycler 480 DNA SYBR Green I Master Kit (Roche Applied Science) and the LightCycler^®^ technology (LC480). The primer pairs K24-s (5′-GGAGGTGGTTCTAGTTTTGCA-3′) and K24-a (5′-GACGAGACAACCTTGCCATC-3′), K12-s (5′- GCTCGCCATGAAGAAATCCC-3′) and K12-a (5′-CTTTCCTCCAAACCATCACCTT-3′), K3-s (5′-ATCGAGGGTGTCAAGAAGCA-3′) and K3-a (5′-GACATCCTGTACTCCTCGCC-3′), K10-s (5′-GTGGGCGAGTCTTCATCTAA-3′) and K10-a (5′-GAGACTCTTTCCTCTTGATGCA-3′), and GAPDH-s (5′-CAGTCAGCCGCATCTTCTTTTG-3′) and GAPDH-a (5′-CGCCCAATACGACCAAATCC-3′) were used for the amplification of human K24, K12, K3, K10, and glyceraldehyde 3-phosphate dehydrogenase (GAPDH), respectively. mRNA levels were normalized to the GAPDH level of each sample. A published mathematical model was used for calculating the quantities of target relative to reference transcripts^48^. Statistical analyses were performed using a two-tailed Mann-Whitney U Test with GraphPad Prism version 5.01 (GraphPad Software, San Diego, CA, USA).

### Immunofluorescence analysis

Cornea and epidermis samples were embedded in optimal cutting temperature (OCT) medium and frozen. The tissues were sectioned at 4 µm thickness and fixed in acetone at −20 °C. In other experiments, cornea was fixed in 4.5% phosphate-buffered formalin, embedded in paraffin according to a published protocol^15^, sectioned at 4 µm thickness and subjected to antigen retrieval with citrate buffer at pH 6. Paraffin-embedded esophagus from another study^49^ was investigated in parallel. The sections were incubated with rabbit anti-K10 (Covance, PRB159P, 1:1000), guinea pig anti-K12 (Progen, GP-K12, 1:1000), mouse monoclonal anti-K14 (Abcam, ab7800, 1:200), guinea pig anti-K72 (Progen, GP-K6irs2, 1:1000) as a negative control, and guinea pig anti-K24 (1:1000). The latter antiserum was raised against the synthetic peptide C-SGSVNMGSEDLVSGD, corresponding to amino acid residues 466–480 of human K24, coupled to keyhole limpet protein.

The fluorescence-labelled secondary antibodies, raised in goat, were anti-guinea pig immunoglobulin G (IgG) conjugated to Alexa Fluor 594 (Invitrogen, A-11076, 1:500), anti-rabbit IgG conjugated to Alexa Fluor 546 (Invitrogen, A-11010, 1:500), and anti-mouse IgG conjugated to Alexa Fluor 488 (Invitrogen, A11001, 1:500). 10% goat serum (DAKO) was added to the secondary antibody to suppress unspecific binding. Nuclei were counterstained with Hoechst 33258 solution (bisBenzimid H 33258, Sigma Aldrich, B2883).

### Western blot analysis

Proteins from cornea and epidermis samples were homogenized with the Precellys system (VWR International, Radnor, PA) in Laemmli extraction buffer containing 2% SDS. Protein concentrations were determined with the Micro BCA protein assay kit (Thermo Fisher Scientific). Thirty µg of total protein per lane were electrophoresed through an ExcelGel SDS 8–18% polyacrylamide gradient gel (GE Healthcare Life Sciences, Chicago) and blotted onto a nitrocellulose membrane. The membrane was incubated with guinea pig anti-K24 (dilution 1:1000) overnight at 4 °C. After washing, the membrane was incubated for 1 h at room temperature with goat anti-guinea pig IgG (Abnova, AB10611, 1:10000) coupled to horseradish peroxidase. Bands were visualized using the enhanced chemiluminescence system (SuperSignal West Dura Extended Duration Substrate, Thermo Fisher Scientific). The membrane was reincubated with monoclonal mouse anti-K14 (Abcam, ab7800, 0.33 µg/ml) and sheep anti-mouse IgG (GE Healthcare, NXA931V, 1:10000) coupled to horseradish peroxidase as a secondary antibody.

### Ethical approval and informed consent

This research has been approved by the ethics committee at the Medical University of Vienna (ethics approval numbers EK1578/2013 and EK2011/1149). All procedures involving human tissues were in accordance with the ethical standards of the institutional research committee and with the 1964 Helsinki declaration and its later amendments. This research has been approved by the ethics committee at the Medical University of Vienna. Postmortem cornea samples were procured from the cornea bank at the Medical University of Vienna (ethics approval number EK1578/2013). Human skin was obtained from plastic surgery (ethics approval number EK2011/1149). All donors provided written informed consent. No tissues were procured from prisoners.

## Supplementary information

Supplementary Information

## Data Availability

The datasets generated and analysed during the current study are available from the corresponding author on reasonable request.
