# Supplementary material for: Comparative genomics suggests loss of keratin K24 in three evolutionary lineages of mammals
Source: Sci Rep. 2019 Jul 29;9:10924. doi: 10.1038/s41598-019-47422-y (PMC6662840; doi:10.1038/s41598-019-47422-y)
Supplement: Supplementary file 1 — Supplementary Information [file 41598_2019_47422_MOESM1_ESM.pdf]

Supplementary Information

Comparative genomics suggests loss of keratin K24 in three evolutionary lineages of mammals

Florian Ehrlich, Maria Laggner, Lutz Langbein, Pamela Burger, Andreas Pollreisz, Erwin Tschachler, Leopold Eckhart

Content

Supplementary Tables S1-S7

Supplementary Figures S1-S6

Supplementary Table S1. *Krt24* and neighboring genes of the tarsier (*Carlito syrichta*)

Gene (GenBank)	Gene ID (GenBank)	Protein[1]	Functional protein	Genomic DNA sequence (acc. nr.)	CDS start	CDS end	Protein[2] (GenBank acc. nr.)	Comments
Krt222	103258098	K222	unknown	NW_007246053	399150	406060	XP_008054006.1	isoform x1
Krt24	103258101	K24	yes	NW_007246053	337538	342682	XP_008054008.1	
Krt25	103258102	K25	yes	NW_007246053	305501	311642	XP_008054009.1	
Krt26	103258103	K26	yes	NW_007246053	285481	294230	XP_008054010.1	
Krt27	103258104	K27	yes	NW_007246053	265510	271275	XP_008054012.1	
LOC103258105	103258105	K28	yes	NW_007246053	245175	253206	XP_008054013.1	
Krt10	103258106	K10	yes	NW_007246053	221048	225293	XP_008054014.1	
Krt12	103258107	K12	yes	NW_007246053	173823	180191	XP_008054015.1	

Notes: [1]Keratins were named based on orthology according to molecular phylogenetics (Suppl. Fig. S2) and gene synteny (Fig. 4).
[2]Accession numbers (acc. nr.) of amino acid sequences predicted in GenBank.
CDS, coding sequence. Genome assembly accession number: GCF_000164805.1

Supplementary Table S2. *Krt24* and neighboring genes of the sifaka (*Propithecus coquereli*)

Gene (GenBank)	Gene ID (GenBank)	Protein[1]	Functional protein	Genomic DNA sequence (acc. nr.)	CDS start	CDS end	Protein[2] (GenBank acc. nr.)	Comments
Krt222	105823591	K222	unknown	NW_012135969.1	3526214	3533105	XP_012516298.1	isoform x1
LOC105823668	105823668	K24	yes	NW_012135969.1	3493682	3497808	XP_012516370.1	termed "*Krt24-like*" in GenBank, phylogenetics suggest *Krt24* ortholog
Krt24	105822735	K223	yes	NW_012135969.1	3481193	3484760	XP_012515228.1	termed "*Krt24*" in GenBank, phylogenetics suggest *Krt223* ortholog
Krt25	105823131	K25	yes	NW_012135969.1	3455796	3461095	XP_012515689.1	
Krt26	105823124	K26	yes	NW_012135969.1	3446158	3446158	XP_012515676.1	
Krt27	105823626	K27	yes	NW_012135969.1	3435617	3440377	XP_012516331.1	
Krt28	105823546	K28	yes	NW_012135969.1	3423921	3428924	XP_012516241.1	
Krt10	105823517	K10	yes	NW_012135969.1	3403177	3407595	XP_012516213.1	
Krt12	105823526	K12	yes	NW_012135969.1	3356250	3361040	XP_012516221.1	

Notes: [1]Keratins were named based on orthology according to molecular phylogenetics (Suppl. Fig. S2) and gene synteny (Fig. 4).
[2]Accession numbers (acc. nr.) of amino acid sequences predicted in GenBank.
CDS, coding sequence. Genome assembly accession number: GCF_000956105.1

Supplementary Table S3. *Krt24* and neighboring genes of the galago (*Otolemur garnettii*)

Gene (GenBank)	Gene ID (GenBank)	Protein[1]	Functional protein	Genomic DNA sequence (acc. nr.)	CDS start	CDS end	Protein[2] (GenBank acc. nr.)	Comments
Krt222	105886671	K222	unknown	NW_003852413.1	23372033	23379764	XP_012658445.1	isoform x1
LOC100964857	100964857	K24	yes	NW_003852413.1	23325061	23329316	XP_012658460.1	termed "*Krt24-like*" in GenBank, phylogenetics suggest *Krt24* ortholog
LOC100964538	100964538	K223	yes	NW_003852413.1	23309790	23314122	XP_012658359.2	termed "*Krt24*" in GenBank, phylogenetics suggest *Krt223* ortholog
Krt25	100945237	K25	yes	NW_003852413.1	23282591	23288982	XP_003786433.1	
Krt26	100964224	K26	yes	NW_003852413.1	23270123	23275284	XP_012658458.1	
Krt27	100944916	K27	yes	NW_003852413.1	23258331	23263272	XP_003786432.1	isoform x2
Krt28	100944600	K28	yes	NW_003852413.1	23239544	23246348	XP_003786431.1	
Krt10	100963915	K10	yes	NW_003852413.1	23213716	23216725	XP_012658358.1	predicted: exon 7 starts in sequence gap, highly conserved end with both splice forms; K10 exon 7 (<23217416-23217471), K10x1 exon 7 (<23217416-23217491) K10 exon 8 (23217795-23217801), K10x1 (23217795-23217901); compare Ehrlich et al, 2019
Krt12	105886317	K12	yes	NW_003852413.1	23178460	23184046	XP_012658357.1	

Notes: [1]Keratins were named based on orthology according to molecular phylogenetics (Suppl. Fig. S2) and gene synteny (Fig. 4).
[2]Accession numbers (acc. nr.) of amino acid sequences predicted in GenBank.
CDS, coding sequence. Genome assembly accession number: GCF_000181295.1

Supplementary Table S4. *Krt24* and neighboring genes of the tree shrew (*Tupaia chinensis*)

Gene (GenBank)	Gene ID (GenBank)	Protein[1]	Functional protein	Genomic DNA sequence (acc. nr.)	CDS start	CDS end	Protein[2] (GenBank acc. nr.)	Comments
Krt222	102501437	K222	unknown	NW_006159986	2121646	2130972	XP_006156005.1	isoform x1
LOC102502411	102502411	K24	yes	NW_006159986	2087226	2091315	XP_006156008.1	termed "*Krt24-like*" in GenBank, phylogenetics suggest *Krt24* ortholog
LOC102469611	102469611	K223	yes	NW_006159986	2056697	2062555	XP_014446146.1	termed "*Krt24*" in GenBank, phylogenetics suggest *Krt223* ortholog
Krt25	102502843	K25	no	NW_006159986	2035545	2041559	XP_006156009.1	mutation: 5 nucl. deletion at nucl. 2035659 compared to *Callithrix jacchus* (common marmoset)
Krt26	102503272	K26	yes	NW_006159986	2023668	2029246	XP_006156010.1	
Krt27	102467926	K27	yes	NW_006159986	2013262	2017296	XP_006156011.1	
Krt28	102470045	K28	yes	NW_006159986	1990416	2000152	XP_006156097.2	
Krt10	102470190	K10	yes	NW_006159986	1973301	1977526	XP_006156016.1	
Krt12	102469180	K12	yes	NW_006159986	1928238	1933431	XP_006156014.1	isoform x1

Notes: [1]Keratins were named based on orthology according to molecular phylogenetics (Suppl. Fig. S2) and gene synteny (Fig. 4).
[2]Accession numbers (acc. nr.) of amino acid sequences predicted in GenBank.
CDS, coding sequence. Genome assembly accession number: GCF_000334495.1

Supplementary Table S5. *Krt24* and neighboring genes of the tasmanian devil (*Sarcophilus harrisii*)

Gene (GenBank)	Gene ID (GenBank)	Protein[1]	Functional protein	Genomic DNA sequence (acc. nr.)	CDS start	CDS end	Protein[2] (GenBank acc. nr.)	Comments
Krt222	105750385	K222	unknown	NW_003838837.1	573121	589971	XP_012403798.1	isoform x1
LOC100922568	100922568	K224	yes	NW_003838837.1	543679	555755	XP_012403785.1	termed "Krt24-like" in GenBank, phylogenetics suggest Krt224 ortholog, 8 exons, ortholog in opossum
LOC100933924	100933924	K24	yes	NW_003838837.1	525306	531869	XP_012403781.1	termed "Krt24-like" in GenBank, phylogenetics suggest Krt24 ortholog, note: C-terminus of protein, isoform x1
LOC100922314	100922314	K24L	yes	NW_003838837.1	501326	506983	XP_012403786.1	termed "Krt24-like" in GenBank, weak support for Krt223 orthology in ML phylogenetics
Krt25	100933661	K25	yes	NW_003838837.1	471274	483520	XP_003768298.1	
Krt26	100933409	K26	yes	NW_003838837.1	453282	465400	XP_003768297.1	
LOC100933149	100933149	K27	yes	NW_003838837.1	441076	446937	XP_003768296.1	
LOC100932884	100932884	K10L	yes	NW_003838837.1	421357	426627	XP_023358136.1	
LOC105750380	105750380	K10	yes	NW_003838837.1	385688	390976	XP_012403757.1	exon 1 ends in sequence gap, corrected range: 385688 - >386138
Krt12	100921792	K12	yes	NW_003838837.1	326811	338459	XP_012403779.1	

Notes: [1]Keratins were named based on orthology according to molecular phylogenetics (Suppl. Fig. S2) and gene synteny (Fig. 4).

[2]Accession numbers (acc. nr.) of amino acid sequences predicted in GenBank.

CDS, coding sequence. Genome assembly accession number: GCF_000189315.1

Supplementary Table S6. *Krt24* and neighboring genes of the platypus (*Ornithorhynchus anatinus*)

Gene (GenBank)	Gene ID (GenBank)	Protein[1]	Functional protein	Genomic DNA sequence (acc. nr.)	CDS start	CDS end	Protein[2] (GenBank acc. nr.)	Comments
Krt222	100074593	K222	unknown	NW_001601406.1	3916	17863	XP_007654369.1	isoform x1, different scaffold
LOC100091675	100091675	K224	yes	NW_001766343.1	474208	484025	XP_007662430.1	termed "*Krt10-like*" in GenBank, phylogenetics suggests *Krt224*
LOC100091681	100091681	K24L3	yes	NW_001766343.1	461812	466395	XP_001512134.1	copy of K24L1 (Gene ID: 100091718), relation to K223P and K24 uncertain
LOC100091690	100091690	K24L2	yes	NW_001766343.1	447283	452557	XP_007662424.1	copy of K24L1 (Gene ID: 100091718), relation to K223P and K24 uncertain
LOC100091696	100091696	n.a.	no	NW_001766343.1	429260	433491	n.a.	pseudogene, according to position this is very likely human K24 orthologous gene
LOC100091718	100091718	K24L1	yes	NW_001766343.1	409950	414474	XP_001520519.1	called K24L, relation to K24 and K223 uncertain
LOC100091723	100091723	K25	yes	NW_001766343.1	393492	403448	XP_001520523.1	
LOC100091728	100091728	K26	yes	NW_001766343.1	376399	383492	XP_001512895.1	termed K27 in GenBank, K26 ortholog because of gene synteny and phylogenetics
LOC100091669	100091669	K27	yes	NW_001766343.1	360079	366910	XP_001513002.1	
LOC100091735	100091735	K28	yes	NW_001766343.1	344752	354236	XP_001520530.1	
Krt10	100091742	K10	yes	NW_001766343.1	325587	331604	XP_007662425.1	isoform x1
LOC100091750	100091750	K12	yes	NW_001766343.1	308095	314746	XP_007662432.1	called K10L, uncommon N-terminal protein sequence, no C-terminal sequence found

Notes: [1]Keratins were named based on orthology according to molecular phylogenetics (Suppl. Fig. S2) and gene synteny (Fig. 4).

[2]Accession numbers (acc. nr.) of amino acid sequences predicted in GenBank.

CDS, coding sequence; Genome assembly accession number: GCF_000002275.2

n.a., not applicable.

Supplementary Table S7. *Krt24* and neighboring genes of the alligator (*Alligator mississippiensis*)

Gene (GenBank)	Gene ID (GenBank)	Protein[1]	Functional protein	Genomic DNA sequence (acc. nr.)	CDS start	CDS end	Protein[2] (GenBank acc. nr.)	Comments
Krt222	102567256	K222	unknown	NW_017707913	11697580	11706381	XP_006276728.1	isoform x1
LOC102569129	102569129	K24L	yes	NW_017707913	11672164	11681432	XP_019355566.1	
LOC102568897	102568897	K10L	yes	NW_017707913	11640091	11650073	XP_019355558.1	
LOC102566338	102566338	K12	yes	NW_017707913	11607814	11614109	XP_014460170.1	

Notes: [1]Keratins were named based on orthology according to molecular phylogenetics (Suppl. Fig. S2) and gene synteny (Fig. 4).

[2]Accession numbers (acc. nr.) of amino acid sequences predicted in GenBank.

CDS, coding sequence. Genome assembly accession number: GCF_000281125.3

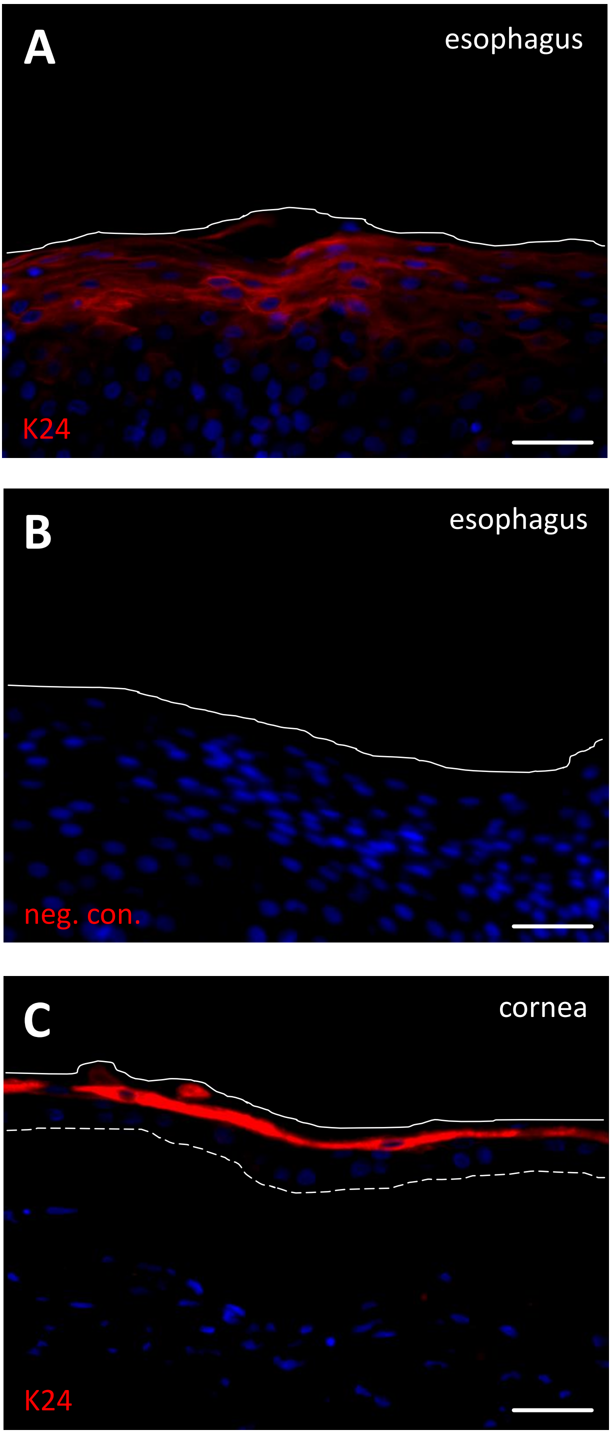

Suppl. Fig. S1. Immunofluorescence labeling of K24 in the esophagus. (A) Formalin-fixed and paraffin-embedded samples from esophagus (Wagner et al. 2019) were immunolabeled for K24 (red). Nuclear DNA was labeled with Hoechst 33258 dye (blue). A representative result of 3 experiments is shown. **(B)** In negative control (neg. con.) experiments, the primary antibody was replaced with an isotype control antibody. **(C)** Immunolabeling of formalin-fixed and paraffin-embedded cornea served as a positive control. The surfaces of the epithelia are indicated by a white continuous line. The junction of the epithelium and the stroma of the cornea is indicated by a broken line. Scale bars, 50 µm. Reference: Wagner T, *et al.* The differentiation-associated keratinocyte protein cornifelin contributes to cell-cell adhesion of epidermal and mucosal keratinocytes. *J Invest Dermatol.* doi: 10.1016/j.jid.2019.04.019 (2019)

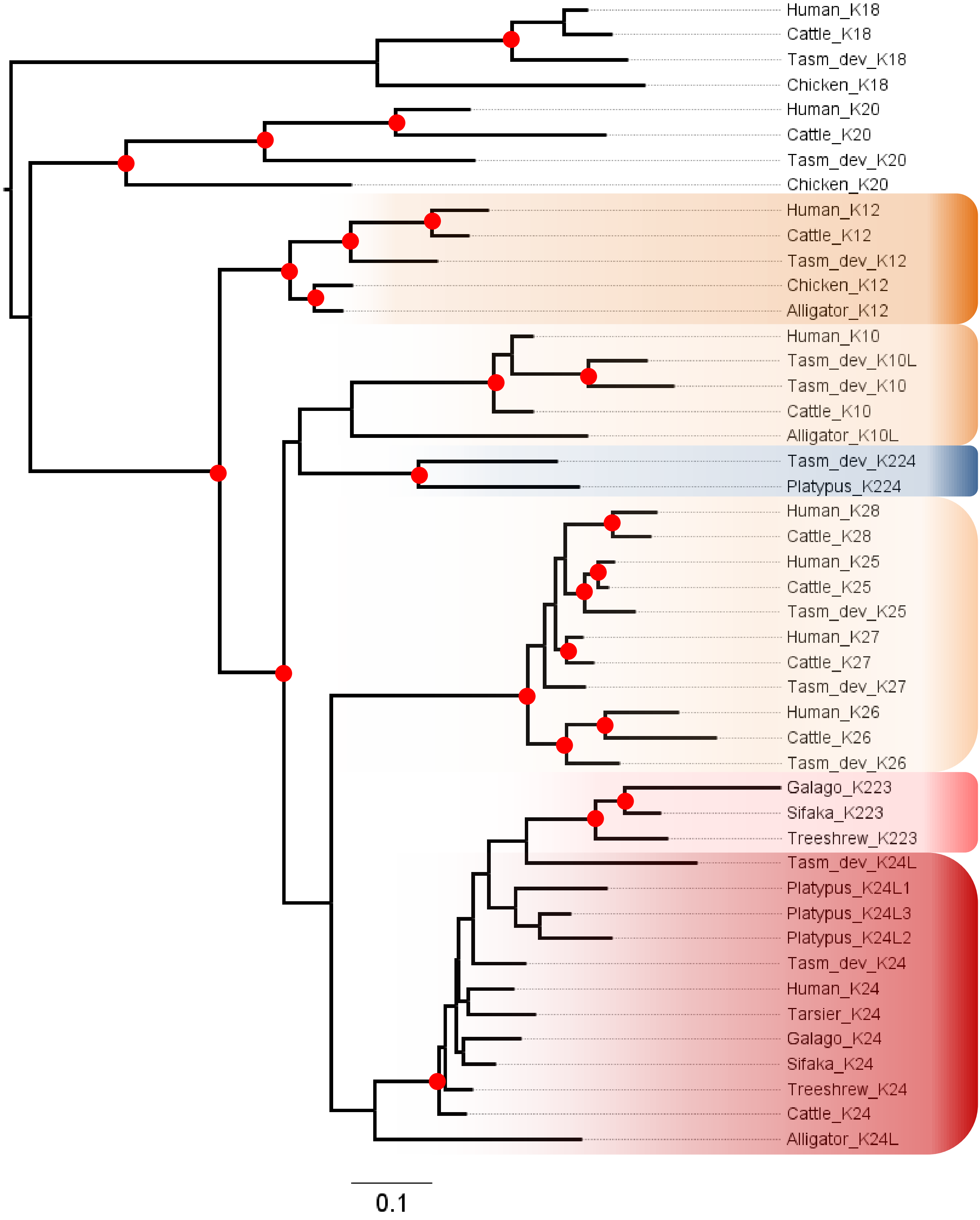

Suppl. Fig. S2. Phylogeny of K24 and related keratins of mammals and sauropsids. Red circles indicate clades with bootstrap support ≥75% obtained by ML analyses, model JTT with 100 replicates and posterior propability >0.95 in Bayesian anaylsis, two parallel Markov chain Monte Carlo (MCMC) runs of four chains each, with a length of 3,500,000 generations and a sampling frequency of 1 per 1,000 generations. L (in keratin names), like; Tasm_dev, Tasmanian devil.

```
                1                                                                                                  100
Hs_KRT224P      AAGTCACACGCAGCCCAAAATCTACTCTATGGCAATTAGATACTGATCATTGGA--------------AAGAAAGAATTATAGGCAGAAATGTAACCCAG
Sh_Krt224       ATGTCATACTCTGTCCAAAAGTCAAGTCTTTGGCAGTTCAGTTCTGGCCAAGGGAGAGGGGGATCCATAAGAAAAAAGCATGGTCCTGGTGGTACCTCAG
Sh_K224_mRNA    ATGTCATACTCTGTCCAAAAGTCAAGTCTTTGGCAGTTCAGTTCTGGCCAAGGGAGAGGGGGATCCATAAGAAAAAAGCATGGTCCTGGTGGTACCTCAG
Md_Krt224       ATGCCATACTCAGTCCAAAAATCGAGCCTGTGGCAGTTCAGTTCTGGCCAGGGGAGAGGGGGAACCGCAGGGAAAAAGTATGGTCTGAGTGGTTCCCTGG
Md_K224_mRNA    ATGTCATACTCTGTCCAAAAGTCAAGTCTTTGGCAGTTCAGTTCTGGCCAAGGGAGAGGGGGATCCATAAGAAAAAAGCATGGTCCTGGTGGTACCTCAG

                101                                                                                                200
Hs_KRT224P      ATGGAGATTTTCTACCAACTT------------------GGGAGGAGGTGGGTGGGTTCTTCTCTGGTAAGAAGAAGGATGCAGTGTAAAATCTAGATGA
Sh_Krt224       GCGGAGTCTTCAGTAGTACCTTTGGTGGATATACTTTTGGTGGAAGGGAAAATGGGCTTCTCTCTGGCAATGAAAAGGGCACCATGCAAAATCTGAATGA
Sh_K224_mRNA    GCGGAGTCTTCAGTAGTACCTTTGGTGGATATACTTTTGGTGGAAGGGAAAATGGGCTTCTCTCTGGCAATGAAAAGGGCACCATGCAAAATCTGAATGA
Md_Krt224       GGGGAAGCTTCAGTAGTACCTTTGGTGGGTATAATTTTGGTGGGGGAGAAAATGGTCTTCTCTCTGGCAATGAAAAGGGAACTATGCAGAACTTGAATGA
Md_K224_mRNA    GGGGAAGCTTCAGTAGTACCTTTGGTGGGTATAATTTTGGTGGGGGAGAAAATGGTCTTCTCTCTGGCAATGAAAAGGGAACTATGCAGAACTTGAATGA

                201                                                                                                300
Hs_KRT224P      CCATGTTGCAGTTTCCCTGGACAAGGTCTCAACTCTGGTGCAGGCAAAGAGTACACTTGAATATAAAATCAAAGAATGGAATAAGAAATTTGAGCCTGGG
Sh_Krt224       CCGCCTGGCAATCTATCTAGATAAGGTTCGAGCTTTAGAGGAAGCAAACACTGAACTTGAATATAAAATTAAAGAATGGTATAAGAAATTTGGACCTGGC
Sh_K224_mRNA    CCGCCTGGCAATCTATCTAGATAAGGTTCGAGCTTTAGAGGAAGCAAACACTGAACTTGAATATAAAATTAAAGAATGGTATAAGAAATTTGGACCTGGC
Md_Krt224       CCGCCTGGCAGTCTACCTAGATCAGGTTCGAGCTCTGGAGCAGGCAAACAATGAGCTTGAATCTAAAATCAAAGAATGGTATAAGAAATTTGGGCCAGGC
Md_K224_mRNA    CCGCCTGGCAGTCTACCTAGATCAGGTTCGAGCTCTGGAGCAGGCAAACAATGAGCTTGAATCTAAAATCAAAGAATGGTATAAGAAATTTGGGCCAGGC

                301                                                                                                400
Hs_KRT224P      CTAATGA-TCAAGGAGAAAAACAGGACAACAAGAAGTATTACCAGATAATTACAGAACTTAAAAACAAGGTAAGACGTTTTGCTA-------GACAAAGA
Sh_Krt224       AGTACCAGTCATGGAGAAAATCGGGACTACTCTGAATATTACCAAATAATTGAAGAACTCAAACAAAAGGTAAGAAATGTCATTATTGCTAAGATTTGCA
Sh_K224_mRNA    AGTACCAGTCATGGAGAAAATCGGGACTACTCTGAATATTACCAAATAATTGAAGAACTCAAACAAAAG-------------------------------
Md_Krt224       AGTACCAGCCATGGAGAAAATCGGGACTACTCCGAATATTACCAGATCATTAAAGAACTTAAACATAAGGTAAGAAAGTTCATTATTACTAAGATTTGCA
Md_K224_mRNA    AGTACCAGCCATGGAGAAAATCGGGACTACTCCGAATATTACCAGATCATTAAAGAACTTAAACATAAG-------------------------------

                401                                                                                                500
Hs_KRT224P      ACCACACTAAAAATGACAAAATTTGAAAAGATTGGCAATATCTTCCACATATACCTGTACTTTTCTCAATTTGGCAGATTAAGGAAGAAATTTAACAAGC
Sh_Krt224       GTTACATTAAAAACAATAGAC--TCAGTTCCTAG--AGCATCTTATAACCATGCCCATCTTT-GCCAAAGAAAATTAAATCTGTAATTAATGGAACATGT
Sh_K224_mRNA    ----------------------------------------------------------------------------------------------------
Md_Krt224       GTCACACTAAACGTGGTCAGACTTAATTTCCTTG--AGCATCTTGTAACCATGCCCATTTTTTGCTAAGGAAAATGAAATCACTGAATATTAGAAAATGT
Md_K224_mRNA    ----------------------------------------------------------------------------------------------------

                501                                                                                                600
Hs_KRT224P      AACAGAATATACTTCTTAGCTATGTTTTTTTTCCTAATTTGTTTACTTTTGTATAGTTCATTTTACGGAAAGAATTCTAGAAATGTTTCCAAAATACTTA
Sh_Krt224       ATTAAGAAATAATAGTGAATATT-TATTTT-----AATTTCTTTATTCTTATACCATTCACATTA-GGAGGGAAAAATAGAATTATTTCCAAGATTCCAA
Sh_K224_mRNA    ----------------------------------------------------------------------------------------------------
Md_Krt224       ATTGAGAAATAGGAAGGAATGTTCTCTTTT-----AACTTCCTTGTTGTTATACCATTTGTGTTA---AGGGGAGAAAAGAATTATTTCCAAGATTCCAA
Md_K224_mRNA    ----------------------------------------------------------------------------------------------------

                601                                                                                                700
Hs_KRT224P      AGAAAAAGTAA-----ACTAAATTCCTTACAGTTCTTTTATTCTCAAAAGCAGAGGTATATGCTATTTTCCATTAAAGAACCGTATACATATAAAATTTG
Sh_Krt224       AGTTTGATTCACTAGTAATCAACTGCTATGATTTG--TCACTTCCCAAAATTGCATT-TCTTACAAAGAAATGTAAAATCTTTGTTGAATGAATGCTGTT
Sh_K224_mRNA    ----------------------------------------------------------------------------------------------------
Md_Krt224       AGTTTGATTCGTTAGTAATAAATTGCCATCATTATCATCATTTCCCAAAATTGCCTTATCTTAGGAAGAAATGTAAAATAATTGCTGAATGAATGTTGTT
Md_K224_mRNA    ----------------------------------------------------------------------------------------------------

                701                                                                                                800
Hs_KRT224P      TTTGAAAGACCTGAAAT----------------------------------TGACATGCCTCAACACCAGCATACAGAGGTTCAGAATGCAAGTGTATCA
Sh_Krt224       ATTTAAAAACTAGATATTTTGCATATTACTTTAAAAGGCATTTGAAGTGGGTGTCCTGCCATCTGTT-ATGGTTCTATCGCAG-----GGAAATTTAGCA
Sh_K224_mRNA    ----------------------------------------------------------------------------------------------------
Md_Krt224       ATTCAAGAGCTAGATACCTTGAAT-------------GGCCTTGAAGTGGGTGGCCTGCCATCTCACCAAGATTCTATCACAGAACAGGGAAATTTAGTA
Md_K224_mRNA    ----------------------------------------------------------------------------------------------------

                801                                                                                                900
Hs_KRT224P      GACACAACTTTTTGTGTCCTGCGTTTCCAGATTTCAGATGCAAACACTGACAATGCA-ACTTCATGCTACAGATCGTCAGCGTAAAACTAGCAGCTGTGG
Sh_Krt224       TACATTTCATTATGTTTT-TTTATGTCCAGATCTTGGATTCATCTTTGAGCAATACTGACATCATGCTCCAGGTTGATAATGTGAGACTAGCGGCTGATG
Sh_K224_mRNA    ------------------------------ATCTTGGATTCATCTTTGAGCAATACTGACATCATGCTCCAGGTTGATAATGTGAGACTAGCGGCTGATG
Md_Krt224       TG--TTCCTTTATGTTCTCTTTTTGTCCAGATCTTGGATTCAACAATTAGCAATGCCGACATCATGCTCCAAACTGATAATGTGAGACTGGCTGCTGATG
Md_K224_mRNA    ------------------------------ATCTTGGATTCAACAATTAGCAATGCCGACATCATGCTCCAAACTGATAATGTGAGACTGGCTGCTGATG

                901                                                                                               1000
Hs_KRT224P      ACTTCAGACAAAAGTGTGATATAG--------------------------------TTACCCTTAGCAGCATGATGAAAAACATAAGCTATGGAGATAAG
Sh_Krt224       ACTTCAGACTAAAGTAAGAAGTAAAGATCTTGGTGGGAGGGGGAGAGAACTTGATATGAATACAAGTCTCACTAAAAATAACAAACCATATAGTGCTTTG
Sh_K224_mRNA    ACTTCAGACTAAA---------------------------------------------------------------------------------------
Md_Krt224       ACTTCAAACTAAAGTAAGAGGCAAATGGTTGGGCTGGAAGGAAAGGGAATTCAATATTAATGTCAGCCTCACTAGTAATGGTAGGCTATATAGTGCTTTA
Md_K224_mRNA    ACTTCAAACTAAA---------------------------------------------------------------------------------------

                1001                                                                                              1100
Hs_KRT224P      CACTAGGTAAGGCCGGGCGTGG-------------------------------TGGTTCACACCTATAATCCCAACACTT--------TGGGAGGCCGAG
Sh_Krt224       G-GAAGCTGGGTGGTATAGTGGATAGAGTACTGGGCATAGAGTCAGGAAGACCTGATTTTAAATTCGGATTCAGAAACTTACTAGCTGTGTGACTCTGGG
Sh_K224_mRNA    ----------------------------------------------------------------------------------------------------
Md_Krt224       GAGCAGTTAAGTGGCACAATAGATAGAACATTGGACTCATTTTCA--------TGAGTTCAAATCCTGCCTCAGACACTTA---GCTGTGTGACCCTGGG
Md_K224_mRNA    ----------------------------------------------------------------------------------------------------

                1101                                                                                              1200
Hs_KRT224P      GCAGGCGGATCACC--------------------------------------------------------------------------------------
Sh_Krt224       CAAATCACTTAACCCTGTTTGCCTTAGTTTCCTCATCTGTAAAATGAGCTGGAAAATTAAATGGCAAA-------------------AAAAAACCTAAAT
Sh_K224_mRNA    ----------------------------------------------------------------------------------------------------
Md_Krt224       CAAATCACTTAGCCCTGTTGGCCTCAGTTTCCTCATATGTAAAAGGAACTAGAGAAGGAAATGGCAAATCACTCCAGTGTCTTTGTAATAAAACCTCAAC
Md_K224_mRNA    ----------------------------------------------------------------------------------------------------

                1201                                                                                              1300
Hs_KRT224P      ----TGAGGTCAGGAGTTTGAGACCAGACTGACCAATATGATGAAACCC---------------------------------------------------
Sh_Krt224       AGGGTTATGAAGAGTTAGTTGGACACAACTGTGCAACAAAATATAGTGCTTTAGTCCATAGAGAAATTCTCTCACAAAGTATCCCTTATATAGAAAGCAT
Sh_K224_mRNA    ----------------------------------------------------------------------------------------------------
Md_Krt224       TGGATTCACAAAG---ACTCAGACACGATTGAACAATTAAAAAAAGCT-TGGAGTGATGATGTAATGTGCCCATCATTGTGCAGCTAAGTGATTAAACTA
Md_K224_mRNA    ----------------------------------------------------------------------------------------------------

                1301                                                                                              1400
Hs_KRT224P      ---------------CATCTCTACTAAAAATACAAAAATTAGCCAGGCATGGTGGCATACACCTGCAATCCCAGCTACTCAGGAGGCTGAGACAGGAGAA
Sh_Krt224       AAATGTTATTTTCTTCATAGAGGAGAAAGAGGATATGCCAAAGTTGATTTAGAGGTATTGGAATCCTGGCTCTGCTACTCACTATCTGTGTGAAATTGGG
Sh_K224_mRNA    ----------------------------------------------------------------------------------------------------
Md_Krt224       GAAGGCAAGCCAAGACCTTTCCATTCCAGATCCAAGTCTTCAGATTAGATTTCTTCTTTCAGCTCTTTCCACTACAACACAGCTGTCAGTAACAGGGAGG
Md_K224_mRNA    ----------------------------------------------------------------------------------------------------

                1401                                                                                              1500
Hs_KRT224P      TCGCTTGAATCCGGGAGGCAGAGGTTGCAGTGAGCTGAGGCCATACCATCGCACTCCAGCCTGGGCAACAAGAGCGAAACTCCGTCTCAAAAAAAAAAAA
Sh_Krt224       CAAGTCATATC----TCTCTAAGTTTCTTTATTTATAAAAGGAGGATGTTAAACTAAATTATTTTTAAGGTCCCCTTAATATT---TTAACATAAGACAG
Sh_K224_mRNA    ----------------------------------------------------------------------------------------------------
Md_Krt224       GGGGGAAGCAC----ATAAATAGTGTTTATTTTCCTAATGGTGCTATAATACATTTAGCACTGCCTTAGACCTTATGAAACAGGAATAACCTTGAGGAAA
Md_K224_mRNA    ----------------------------------------------------------------------------------------------------

                1501                                                                                              1600
Hs_KRT224P      AAAAAAAAAAAAAAAAAACACTATGTGAAGCCATGCTTCTTTTTACACTGGAATCACAGTTATA------------------------------------
Sh_Krt224       AGGGGAAAAGCACCTGGATTCTCTGAGAATCTAAGAGTATCCAGAGTCAAAAGTTATTGAGTCATTTCAGTCATGTCCAATTCTTCATGACTTTATTTGA
```

```
Sh_K224_mRNA   ----------------------------------------------------------------------------------------------------
Md_Krt224      AAAAGCCAAAAAGAAAGGTACATAAAGAATGTCTATTTCCCTGCTGGCCCTACAGGTTGTTAGAACAGTGCCTTAAAC---------TTAATAGAATAGA
Md_K224_mRNA   ----------------------------------------------------------------------------------------------------

               1601                                                                                            1700
Hs_KRT224P     -----------------------AAAGAATATTTTTCTTCTTAGAAACAGAATCCTAAACAATGGAAAAA------------------------------
Sh_Krt224      GGGTTTCTTGGCAGAAATACAGGAAGGGTTTGTCACTTTCTTCATTTTACAAATGAGAAAACTAAGACAAGCAGGTTAGAATTTGCCTAGGGACACACAG
Sh_K224_mRNA   ----------------------------------------------------------------------------------------------------
Md_Krt224      GGAAATCCTGGGAGATGACCATTATTGTATCTTATTTTACATAATTAGGGGATCTCATTGCCTACAAGAATCCTACATAATTTATATTA-----------
Md_K224_mRNA   ----------------------------------------------------------------------------------------------------

               1701                                                                                            1800
Hs_KRT224P     -TATTTTAGTTAAAAACCTCAGGGAAGCACATTATTTACGAGAATGCTGTGCTGATTCTACGTCCGAAGGAACCACCACTTAATTTTCTTCCGTAAGTAA
Sh_Krt224      CTAGTGTCAGATTTGAGTTCAAGTTCTCCCAACTCCAGTGCCAGCACTCTACCACTTGTGTGGTCTTAGGAAAGTCAATTAACTT---TTATGGGTCATA
Sh_K224_mRNA   ----------------------------------------------------------------------------------------------------
Md_Krt224      -TATTATATTATATTATATTATATTATATTATATTATATTATATTATATTATATTATATTATATTATATTATATTACATTACATTACATTATATTATATT
Md_K224_mRNA   ----------------------------------------------------------------------------------------------------

               1801                                                                                            1900
Hs_KRT224P     GAATTTCTAGTTAGG-------------------------------------------------------------------------------------
Sh_Krt224      GTTTCTTCATCTGTGAAATGAGGGGATTTAAACTAGATCCCTTCTGGGATTCTTTCCAGTTTTTGATTTACATGCCTGTAATCCACTTTGCTGAACATTG
Sh_K224_mRNA   ----------------------------------------------------------------------------------------------------
Md_Krt224      ATATTATATTATACTATATTATACTATATTATACTATATTATACTATATTATATTATGTTATGTTATGTTATGTTATGTTATGTTATGTTATGCTATGCT
Md_K224_mRNA   ----------------------------------------------------------------------------------------------------

               1901                                                                                            2000
Hs_KRT224P     ------------------------------------------------------------------------------------TATATTTCAAGTGAGA
Sh_Krt224      GAGAAATGATATAATTTGC-ACATTATATAGCTCAGAAGCAATTGAACTAGAACTCGAACATAAG---TCACAAGTCTTCAGATTCAATTTCTCCTTTTA
Sh_K224_mRNA   ----------------------------------------------------------------------------------------------------
Md_Krt224      ATGCTATGCTATGCTATGCTATATTGTATCGTATCGTATCGTATCATATCATATCATATCATATCATATCATATCATATCATATTATATTATATTATTAC
Md_K224_mRNA   ----------------------------------------------------------------------------------------------------

               2001                                                                                            2100
Hs_KRT224P     TTTCTGAAAAGAACCAGCATGTGTTTCTGAGGATTCTTTAATTTAACTCCCTACTTTATGTGTGTGTAGAAGACCCCAACACACAAGAGGCCAACAAGGT
Sh_Krt224      ACTATTTCCACTAAAATACAGCTATTCTTAATAGGGAAAAATAGATATGTAAATAGTAATTGTTTCTCTAAAGGCCCTATAATACATATTATAGCAGTGC
Sh_K224_mRNA   ----------------------------------------------------------------------------------------------------
Md_Krt224      ATTATATTACATTATATCATATATTATATTATATTGTATCATATATCATATTATGTTATGTTATGCTATGCTATGC-TATGCTATATTATATTGTATTGT
Md_K224_mRNA   ----------------------------------------------------------------------------------------------------

               2101                                                                                            2200
Hs_KRT224P     ATGGTCACTCTG----------------------------------------------------------------------------------------
Sh_Krt224      TTTAGACCTATTGGAATAGGGGAAATCTTGGGAAATGATGACTACTGAATCTTATTTCACATAAAGGCACTAGGGTGCTCACAGGAATCCTACATAATAA
Sh_K224_mRNA   ----------------------------------------------------------------------------------------------------
Md_Krt224      ATTA----TATTAGATTATATTGTATTATATTATATTATATCATATCA-TATCATATTATATTATTACATTATATTACATTACATTATATCATATATTAT
Md_K224_mRNA   ----------------------------------------------------------------------------------------------------

               2201                                                                                            2300
Hs_KRT224P     --------------------------------------------------------------------------------------------TTCTAAGA
Sh_Krt224      CTAACACTT----ACAGAACACATTAAGGTTTGTAAGATGTGGCCTTTGGCATTCTCAGAAAGGACATCACCAAACCACAAATTCTGGCAGATCCTTTTA
Sh_K224_mRNA   ----------------------------------------------------------------------------------------------------
Md_Krt224      ATTACATTGTATCATATATCATATTATGTTATATTATGT-TGTCATATTAAATTATATTATTTTATATTGTTATGCTATGTTATATGTAACATTAAAATT
Md_K224_mRNA   ----------------------------------------------------------------------------------------------------

               2301                                                                                            2400
Hs_KRT224P     AACAAAAATGCTGAAAAGGTTCTTCCCCGAAGCTTGCAGCATTTTATATGGAGTGCCATCATACTGCCAAAAATGGGGAAA------------GTTAATA
Sh_Krt224      AATACTAGTGCCATGATTGACTGTTTGTGACTGAAGAAACAGCTTAGGTTCATAACCAGGCCCAGCCCTAGAAGGAGGAGATTTTTGATAGCAGAAACCC
Sh_K224_mRNA   ----------------------------------------------------------------------------------------------------
Md_Krt224      TGCAAAA-TGCTTTATAGCTAGCTTTTCCAAATATTACACAGCTCCGATTTTTCCTCAAACAACCCCTGGGAGGTAGAAGCTATT--------GTTATTC
Md_K224_mRNA   ----------------------------------------------------------------------------------------------------

               2401                                                                                            2500
Hs_KRT224P     GCAAATTATATTAACAACAAATAAGGAACAGACGT-TTGGCATTTCTATAATATTCTAATAAAATA----------------------------------
Sh_Krt224      TGAAAGTATCTACCAAGCAACAGAGGGATTGAAATCTACCCCTCTCAATAAAATTATAATTTAATTCAGTTCAATTGGATAAAAAGTTATTAGTAGCAAG
Sh_K224_mRNA   ----------------------------------------------------------------------------------------------------
Md_Krt224      TCATTTTATAGTTAAAGAAACTGAGCCGTGGTAGGGATGACTGTGGAATAGTAAAGCAAGAACAGGACTTAGGTTTGAAAATCCAGGCTCCATTTGAGAG
Md_K224_mRNA   ----------------------------------------------------------------------------------------------------

               2501                                                                                            2600
Hs_KRT224P     ----------------------------------------------------------------------------------------------------
Sh_Krt224      TGCAAGGCACTGCAAAAAAAATTAGAAACAAAAATAACCCTTTGCCCTTTTGTCATTGAACTATTTCAGTTGTGCCCAACTCTTCATGACTCCATCTGTG
Sh_K224_mRNA   ----------------------------------------------------------------------------------------------------
Md_Krt224      TCT---------CTTCACCCCTCGGAAGCTCAGGCAACTCTAAGACTTTTATTGTTGAATCTTTTCAGTTGTTATCTATCTTTTCATGACCCCATTTGGG
Md_K224_mRNA   ----------------------------------------------------------------------------------------------------

               2601                                                                                            2700
Hs_KRT224P     -----------------------------------TTTAAAATGCAGCATGATATTAATAATACAAACCTAACG--------------------------
Sh_Krt224      GTGTTCTTGGAAAAGATACTGAAGTGATTTGTCTTTTCTTTATCCAACTCATTTTACAAATGAGGAAACTGAG--------------TGACTTGCCCAGG
Sh_K224_mRNA   ----------------------------------------------------------------------------------------------------
Md_Krt224      GTTTTCTTGGCAAAGATACTGGAGTAATTTGCCATTTTTTTCTCCTGCTCCTTTTATATAGGAGAAAACTGAGGCAAAAGGATTAAGTGACTTGCCCAG-
Md_K224_mRNA   ----------------------------------------------------------------------------------------------------

               2701                                                                                            2800
Hs_KRT224P     ----------------------------------------------------------------------ACCACTGAAAGCATT---------------
Sh_Krt224      GTCACATAGCTAGGAAGTATCTGATGTTGGATTTGAACTTAGAAAGATGAATTGGCCTTACTCTAGGCTCACCACTCTATTCATTGGGTCACCTAGCTGC
Sh_K224_mRNA   ----------------------------------------------------------------------------------------------------
Md_Krt224      -TTACCTAGCTCAGAAGTATCTGAGGTCACATTTGAACCTGGATCCTA-------CCGACTTCCAGGTCTAGTACTCTATCCACTGTGCTCACTACCTGC
Md_K224_mRNA   ----------------------------------------------------------------------------------------------------

               2801                                                                                            2900
Hs_KRT224P     ----------------------------------------------------------------------------------------------------
Sh_Krt224      CCCCTTACCCCAAAACCTCAAGGAATACATGTTCTATTAAATAGCTAAAGTACTAAAGTATTGGAGTCTTTTTAGTGGGAAAATGACCTGACACCATGGA
Sh_K224_mRNA   ----------------------------------------------------------------------------------------------------
Md_Krt224      CACATTA---------------------------------------------------------------------------------------------
Md_K224_mRNA   ----------------------------------------------------------------------------------------------------

               2901                                                                                            3000
Hs_KRT224P     ----------------------------------------------------------------------------------------------------
Sh_Krt224      TAGAGCTCTGAATTAGAGTCAGAGGGCCTGAATTCAAAACTTAGCTCTACTACTCAGTAATTGTGTGACCATGAAGAAATCACTCTCTGGGTCTCAATCT
Sh_K224_mRNA   ----------------------------------------------------------------------------------------------------
Md_Krt224      ----------------------------------------------------------------------------------------------------
Md_K224_mRNA   ----------------------------------------------------------------------------------------------------

               3001                                                                                            3100
Hs_KRT224P     ----------------------------------------------------------------------------------------------------
Sh_Krt224      TTTTATTGTAAAATGAAGGGGTTGAATTATATGGCCTCTAAATCTGTAATCTTGAGAACCAACTTCAGAAAATACTGAATTAATTTCTAAGAACTATTTC
Sh_K224_mRNA   ----------------------------------------------------------------------------------------------------
Md_Krt224      ----------------------------------------------------------------------------------------------------
Md_K224_mRNA   ----------------------------------------------------------------------------------------------------
```

```
             3101                                                                                               3200
Hs_KRT224P   ----------------------------------------------------------------------------------------------------
Sh_Krt224    TACCAATACTATAATTTTAAAAGTGTAAAACAAAATTTATATCAAAATGATGTCTTCTTTTGTTATCTCTAATTTATTCTGTATGTAGCTTATTTCTACA
Sh_K224_mRNA ----------------------------------------------------------------------------------------------------
Md_Krt224    ----------------------------------------------------------------------------------------------------
Md_K224_mRNA ----------------------------------------------------------------------------------------------------
             3201                                                                                               3300
Hs_KRT224P   ----------------------------------------------------------------------------------------------------
Sh_Krt224    TAGCTGTTTGCATGTTGTTTCCCCATTAGACTGTGTTCCTCAAAGACAGAAACTGTCTTTTGCTTTTCTTTGTTTCCCCAGCTCTTAATATAGTGTCTGG
Sh_K224_mRNA ----------------------------------------------------------------------------------------------------
Md_Krt224    ----------------------------------------------------------------------------------------------------
Md_K224_mRNA ----------------------------------------------------------------------------------------------------

             3301                                                                                               3400
Hs_KRT224P   -------------------------------------------------------AGAAACTTTTTCCAGGTTTGAGATTGAACTGACCCTGCGCTGGAG
Sh_Krt224    CATATCAGAGGAATTTAATAAATATTTATTGACTGATTTAATAAAAAAATTGTCAACATTTTTTTCAAGGTATGAGACTGAACTGGCCCTCCGCCAGAG
Sh_K224_mRNA --------------------------------------------------------------------GTATGAGACTGAACTGGCCCTCCGCCAGAG
Md_Krt224    -----------------ATGAATATTTATTGACTCA---------AAATTTTTTCAACATTTTTTCCAAGGTATGAAACTGAACTGGCCCTTCGCCAGAG
Md_K224_mRNA --------------------------------------------------------------------GTATGAAACTGAACTGGCCCTTCGCCAGAG

             3401                                                                                               3500
Hs_KRT224P   CGTC-AAGCTGACATCAACAGTCTGCACCAAGTCCGGAATGACATGACTTTGTCCAAGAT-GATCTAGAAATGCAGATTGAAACCCGG-CGGAGGAGTTC
Sh_Krt224    TGTGGAATCTGATATCAATGGTCTACGAAGAGTCTTGGATGACCTGACTTTGGCCAAGACAGACCTAGAGATGCAAATTGAAACCCTGTCAGAGGAAGTG
Sh_K224_mRNA TGTGGAATCTGATATCAATGGTCTACGAAGAGTCTTGGATGACCTGACTTTGGCCAAGACAGACCTAGAGATGCAAATTGAAACCCTGTCAGAGGAAGTG
Md_Krt224    TGTAGAATCTGATATCAATGGTCTACGAAGAGTCCTGGATGACCTGACTTTGGCCAAGACGGACCTGGAGATACAGATCGAAACCCTGTCAGAGGAAGTG
Md_K224_mRNA TGTAGAATCTGATATCAATGGTCTACGAAGAGTCCTGGATGACCTGACTTTGGCCAAGACGGACCTGGAGATACAGATCGAAACCCTGTCAGAGGAAGTG

             3501                                                                                               3600
Hs_KRT224P   ACTCATCTCAAAAAGAACCAGGAAGAGGTATGTGCCCATACAGC---------ACGTCGTAACTTTCCTCTTTCTTACATAATTCCTGCCTTCAATTTGA
Sh_Krt224    GCTTATCTCAAGAAGAACCATGCAGAGGTATACATATATCCAGACACCAAAATAAACATCACCTTTCAAATATTTTCCTTTAGTTTTTGATAGCAATCTG
Sh_K224_mRNA GCTTATCTCAAGAAGAACCATGCAGAG-------------------------------------------------------------------------
Md_Krt224    GCTTATCTCAAGAAGAACCATGCTGAGGTATGTGTATCTCCAGACACCAAAATTAG-ATCGCCTTTCAGATATTTT------GTTTTGCTCAGAATCGCC
Md_K224_mRNA GCTTATCTCAAGAAGAACCATGCTGAG-------------------------------------------------------------------------

             3601                                                                                               3700
Hs_KRT224P   GTCTGATGAGGTGAAGGAAAATTCTAATAAATGAAGTTATTTTC--------TTACCATAATGGCCGAATTCCTTGAGCGAGGG--TTGTTTCCAATTCA
Sh_Krt224    ATAAGATTAAAAAAAAAAACCATGCTGTGGATGAAGTGGATTCTGTGGTACATTCCTGTTTTTACCAGGTTACTATAACACATTAATTGTTCTTAATTCA
Sh_K224_mRNA ----------------------------------------------------------------------------------------------------
Md_Krt224    ATCGGATGAGGTAAAGAAACTCCTCTGCGGATGAAGCGAATTCCACAACACATTCCTATTTTCACCAAGTCGCTAGAGCTCACTAATTGTTCTTAATCCA
Md_K224_mRNA ----------------------------------------------------------------------------------------------------

             3701                                                                                               3800
Hs_KRT224P   GGAAATTAAAGGCCAGCCAGCTGTTGCAGGCTCTGAGTTGCAGGTGGAATGGGTCAGCCCACCTGGAGCTGATCTAACACAGCTCCT-CATGACATGAGA
Sh_Krt224    GGAAATGAAAGGTCTACAAAAAGCTACAAGTTCGGACGTGAACGTGGAAATGAATGCTGCTCCTGGGACAGATCTTACCAAATATCTGCATGACATGAGA
Sh_K224_mRNA -GAAATGAAAGGTCTACAAAAAGCTACAAGTTCGGACGTGAACGTGGAAATGAATGCTGCTCCTGGGACAGATCTTACCAAATATCTGCATGACATGAGA
Md_Krt224    GGAAATGAAAGGTCTACAAAAGGTGGCAAGTTCAGATGTAAACGTGGAAATGAACGCTGCTCCTGGGACCGATCTTACCAAACTTCTGAACGACATGAGA
Md_K224_mRNA -GAAATGAAAGGTCTACAAAAGGTGGCAAGTTCAGATGTAAACGTGGAAATGAACGCTGCTCCTGGGACCGATCTTACCAAACTTCTGAACGACATGAGA

             3801                                                                                               3900
Hs_KRT224P   ATGCACTATGAAGCCATGGCCAATCACAATCACCAAGATGCTGAAATTAGATTCAACAATAAAGTAAACAAAAAACACAGCCAAGGTCAATAAATCTAGG
Sh_Krt224    GCTCAGTATGAAGCCCTGGCTGAGCAGAACCGCAAAGATGTTGAAACTTGGTTCAATGACAAGGTAAATCAGAGATT--GCTCGAGGTCATTAAGCCAAG
Sh_K224_mRNA GCTCAGTATGAAGCCCTGGCTGAGCAGAACCGCAAAGATGTTGAAACTTGGTTCAATGACAAG-------------------------------------
Md_Krt224    GCTCAGTACGAAGCCCTGGCTGAACAGAATCGCAAAGATGTTGAAACTTGGTTCAATAGCAAGGTAAATCAAAGCAC--GCTGAGGGTCATTAGACCAAG
Md_K224_mRNA GCTCAGTACGAAGCCCTGGCTGAACAGAATCGCAAAGATGTTGAAACTTGGTTCAATAGCAAG-------------------------------------

             3901                                                                                               4000
Hs_KRT224P   ACAATTTCGCTTCACTGATAAATGACTCTGTTTTTATTTTGGTATTTCAGAAAAGAATGCTGCAACGGCAGG---------TTGCCGCTGCTGCATCCAA
Sh_Krt224    TTTTGTCATGTGGG-TAGCACTCAACCGGCTTCATGACAATGTCATTT--CAGAGTGAGGTACTACAGCAGCAAATCTCCTTTGCCAATGATGAATCCAA
Sh_K224_mRNA -------------------------------------------------------AGTGAGGTACTACAGCAGCAAATCTCCTTTGCCAATGATGAATCCAA
Md_Krt224    TTTTATCCTGTCCACTGGCCCTCAGCCAGCTTCATGACGGCATCATTTTTCAGAGCGAGTTGCTACAGCAACAGATCTCCCTTGCCACGGATGAATCCAA
Md_K224_mRNA -------------------------------------------------------AGCGAGTTGCTACAGCAACAGATCTCCCTTGCCACGGATGAATCCAA

             4001                                                                                               4100
Hs_KRT224P   TTCTGCTAAGACTGAGACGAATGTACAGGAGCCAGCCTTGTAGGCCCTGGAGATTGAACTACAGTCTACAGTAGACTTGGTACATAAAAATCTCTCCCTG
Sh_Krt224    TTCTGCCAAGACGGAGATATCAGAGCTGAAACGGACTTCACAAACCCTGGAAATTGAGCTACAGTCAGCCTTAGCTCTGGTAGGTAAAATACAA-----A
Sh_K224_mRNA TTCTGCCAAGACGGAGATATCAGAGCTGAAACGGACTTCACAAACCCTGGAAATTGAGCTACAGTCAGCCTTAGCTCTG---------------------
Md_Krt224    TTCTGCCAAGACAGAGATATCAGAGCTGAAGAGGACTTCACAGACCCTGGAGATTGAGCTGCAGTCAGCCTTGGCCCTGGTAGGTAAAAGTCAACACGGA
Md_K224_mRNA TTCTGCCAAGACAGAGATATCAGAGCTGAAGAGGACTTCACAGACCCTGGAGATTGAGCTGCAGTCAGCCTTGGCCCTG---------------------

             4101                                                                                               4200
Hs_KRT224P   AGTGGGGCCCAGCGCGGTGGCTCACACCTGTATTCCCAGCACTT---------------------------------------------------------
Sh_Krt224    AATGAAAAGCAACAAGGTAATACAGTAGTTAGAACACTGTACCAGAAAGC--------CCTGATTCAAATTCATTCTCAGACACTTAATTAATGACCTCA
Sh_K224_mRNA ----------------------------------------------------------------------------------------------------
Md_Krt224    AATTAGAAACAACTAGGTGGTTCAGTGGCTAGGACACTAGGCCTGGAGTCAGGGTGTCCTGGATTCGAATCAGACCTCAGACACTGA-------------
Md_K224_mRNA ----------------------------------------------------------------------------------------------------

             4201                                                                                               4300
Hs_KRT224P   --------------------------------------------------------------------------TGGGAGGCCGAGGCAGGCAGATCACTC
Sh_Krt224    GGTTCCTCAAATGAAAAATGGAGATCATGATAGCAACCTACCTCACAGGGTTGGTGTGAGATAATATTTGTAAAGTGCCTGGCATAATGAATGCTACCTA
Sh_K224_mRNA ----------------------------------------------------------------------------------------------------
Md_Krt224    ------------------------------------------------------------------TTAGCTCTGTGACCCCAGGCAAGTCACTTACCTT
Md_K224_mRNA ----------------------------------------------------------------------------------------------------

             4301                                                                                               4400
Hs_KRT224P   GAGGTTGGGAGCTCGAGACCAGCCTGACCAACATGGAGAAACTCCATCTCTACTAAAAAAATACAAAATTAGCCAGGCG--------------------
Sh_Krt224    TTATTATTTCTCCATCTACAACTTGTGTTCAACACAGATAATCC-ATCAGTCAAGGAACATTTGTGTACCAGGTACT-GTTAAGCAGTGAAGTTGCCTAG
Sh_K224_mRNA ----------------------------------------------------------------------------------------------------
Md_Krt224    TTTTGTTTTAAATCCTTACCTTCTATCTTAAAATCAATAATTAATATCAGTTCCAAGTCATAAGAGCAGTAAGGACTAGGCAATTGGGGTTAAGACACAC
Md_K224_mRNA ----------------------------------------------------------------------------------------------------

             4401                                                                                               4500
Hs_KRT224P   ---------------------------------------------TGGTGGTGCATGCCTGTAATCCCAGCTACTCAGGAGGCT-GAGGCAGGAGAATTGC
Sh_Krt224    ACAAAGCTAGGCTTTGAGCTTTGTACTTAGCAGAGCTTTTCATTTTTTGGTCCAAACTTGAGGTTTCATCACTTCAGGGACCTCTTGATGGGAAAACTGC
Sh_K224_mRNA ----------------------------------------------------------------------------------------------------
Md_Krt224    AGCAAGAAAGAATCTGAGGTCAAATCTGAACCCAAGACCTCACATGTGTCTCCAGAACTGGCTTTCTATACACTAAGCCACCTAGTTATT-------TGC
Md_K224_mRNA ----------------------------------------------------------------------------------------------------

             4501                                                                                               4600
Hs_KRT224P   TTGAACCCAGGAGGCAGAGGCTGCAGTGAGCCAAGATCACGCCACTGCCCTCCAG---------------------------------------------
Sh_Krt224    CTCCATCAGGGTAGCTCTGGCACTGGTTACTTAGGGATACTGCAAAGCC-------ATCTCAATCATTAAATCTGTCAGTCAGCTAACAAGTA-------
Sh_K224_mRNA ----------------------------------------------------------------------------------------------------
Md_Krt224    CTCAGTTTCTTTACCTGTAAAATGAGGTGGAGAAGGAAATAGCAAATATCTCCAGTATCTTTACCAAGAAAGCTCCAAATGGAATCAAAAAGAGAGGGAC
Md_K224_mRNA ----------------------------------------------------------------------------------------------------

             4601                                                                                               4700
Hs_KRT224P   ----------------------------------------------------------------------------------------------------
Sh_Krt224    -----TTTACTCAATGTCTATTCAATACTCTATTCTATATGACCTGTGTTAAGCCCTTGAGATACAAAGGCAGAAGATACAGGTCCCTAACTTCAAGGAG
```

```
Sh_K224_mRNA  ----------------------------------------------------------------------------------------------------
Md_Krt224     ACAACTGTACAACAAGGTGATGCAGTAGATAGAGATCCAGGACTGGTGTCAAA------AGAATCTGAGTTCAAATCTACCCTCGGATTCTTACTAATGG
Md_K224_mRNA  ----------------------------------------------------------------------------------------------------

              4701                                                                                            4800
Hs_KRT224P    ---------------------CCTGGCCAACAAGAGCGAAACTCCGTCTCAAAACAAAAACAAACAAAAAAAATCTTTCCCTGGGTGAAAACACCATAAA
Sh_Krt224     CTGGAAATCTCATGGTAGAGCCTTCATGTACAAGATTCAGAGGGTGTCCCAAAAAAAATTAGTGCAATTTAAACTCTTAAAGCTAAAAAGGAATCAGAGGT
Sh_K224_mRNA  ----------------------------------------------------------------------------------------------------
Md_Krt224     CTTGGGTTCCTCAGCT-GAGAAGTGGAGATCATGATAGCATCTACCTCCCAGACTCCTTACAAATATTAACTCATTCGATCCTCACACCAACGCTAGTAT
Md_K224_mRNA  ----------------------------------------------------------------------------------------------------

              4801                                                                                            4900
Hs_KRT224P    CCAATTGCCCACACAGACAACTGCACGATTTCACATAAATTCT----------------------CAGCAATATGTTTCCAAAACATGATATTCCCTTT-
Sh_Krt224     T--TAAAACTAGACCAAGACTTTGGGGGCACCATATATAAAGAGGGGGTATCTCAGAGAGGAAAGCATGAGCAGCATGGAGGAACCTGAAAAAACCTCTT
Sh_K224_mRNA  ----------------------------------------------------------------------------------------------------
Md_Krt224     TATTATTTCCCCACCTACAAACTGTGTTCAGCACAGGCAATCCG--------TCAGTCAACAAAGGAAGACTTGTGTGCTAAACAGTGAAGTTACC----
Md_K224_mRNA  ----------------------------------------------------------------------------------------------------

              4901                                                                                            5000
Hs_KRT224P    ----------------------------------------------------------------------------------------------------
Sh_Krt224     GTAAAAGCACACATTTAAGCTGTCTTGAAGGAAACCAGAGCAGTCAGAAGGTGGTAGTGAGGGGGAAGTACGTTCCAATCCAGGGTAAAATGCTCAGTCA
Sh_K224_mRNA  ----------------------------------------------------------------------------------------------------
Md_Krt224     ----AAGGCAAAAATTAAACAGTCGTGATTCTAATCCATTAGGAAAAATCGA-------------ATGCATATACATAAGCTAATCTCAGAAAGGAGACA
Md_K224_mRNA  ----------------------------------------------------------------------------------------------------

              5001                                                                                            5100
Hs_KRT224P    -AAAAAGGGTTTTTTTTAGGGTTCTTAAGAAAAAGCCAAAATCTTGGGCTTTTCCAATTAGTAACTTGGACAG---------------------------
Sh_Krt224     GCAGTTAGCATGTTAGAATGTAGTGTGTGAGAGAGGAATGGTGCAGCTGTTCAAAGAATAGATGATGGGGAAGAGGGGGGA-----------AGAGCCTA
Sh_K224_mRNA  ----------------------------------------------------------------------------------------------------
Md_Krt224     GACATTGGCATGGTTTCATGTCA-GAAACAAAGAGATATTGGATATTTTTGTTATTATTATAATATAGAACATCATTCTGATATTATTGTATATAATATA
Md_K224_mRNA  ----------------------------------------------------------------------------------------------------

              5101                                                                                            5200
Hs_KRT224P    -----------GAAAATTTCCATTTAATGTGTGAAAAACACCTGGAAACAAAATTATTAAC-TGCACATGTCAGGCCCTATTTTGCAAACACGACAGCCA
Sh_Krt224     AGAATGTAGGGGGAGGGCAGATTGTAAAGGGTTTTAAATGTCTTGATAACATTTAAATAGCATTTATATGCCAGGCACTGTTCTAAGTGATTTATAGTTA
Sh_K224_mRNA  ----------------------------------------------------------------------------------------------------
Md_Krt224     TGGTCGTTATATATAATAATATTGTATAATGTATTCAATATAATAATATTATATTATTATCTTTGGTCAAACTTTCA---TTCTCAATGTTAGACAGCTG
Md_K224_mRNA  ----------------------------------------------------------------------------------------------------

              5201                                                                                            5300
Hs_KRT224P    CTGAATTAGCTG--------------------AGATACCACAAATGTCAGCATGAACTTGCTGTTAGGGAAATGTTGGCCCACGTTCAGAATGAGATCAC
Sh_Krt224     TTATCTCATTTGGTCCTCACAATTACCCTAGAAGGTAGGTGATATTATTATTTCCATTTTACAGATGAGGAAAC-TGGAGATACAGAAAGATTATATGAC
Sh_K224_mRNA  ----------------------------------------------------------------------------------------------------
Md_Krt224     GTGTAACAGTGGCT----AGAATGCTGGTCAGGCAGAGCTTGAGTTCATATCCCACTCTAGCCATTGAATAAGCCTTCCGACCCCTGCCAAATAAATATC
Md_K224_mRNA  ----------------------------------------------------------------------------------------------------

              5301                                                                                            5400
Hs_KRT224P    TGTTTCTCTCTC-----------------------CAGGTAGGTGATTAGGCAGGGCTCCTGGCTGAAAGCTTAAAAGAATTAATCTAGATAAAATTCCT
 Md_Krt224    TTAATCTCTGTCTCTCTCAGTTTCCTCATCTCTAAAATGAATGTGCATTAGAATAATGCCTAAGTTGTAGAGCATAAGGACCAAATGAGATCATGTTCTT
 Sh_Krt224    TTATCCAGGATCACGCAACTAGTTAGCAACTGAGGCCAGATTTTAACTCAGGTCTTTTTTACTTCAGTTAAGCCTCTTTCCACTGTACCATCTAATTGTT
Md_K224_mRNA  ----------------------------------------------------------------------------------------------------
Sh_K224_mRNA  ----------------------------------------------------------------------------------------------------

              5401                                                                                            5500
Hs_KRT224P    GCGTATAGGTCAGCTAGGATTTCATTGAATATCAAGAAGTATATATGCTCCTTTTTAAGACGATTTAACATATTTAAACTTGAATAACAGGCTGGGCGCT
Sh_Krt224     CTCAATTACTGAAATGGGAGGGGGATGCCTCAGT---------CAAACTGAAATTTGTTAAAGACCTTAGCTTTAAAAGACCAAAGTCTCCCACTGCATC
Sh_K224_mRNA  ----------------------------------------------------------------------------------------------------
Md_Krt224     TGTATATCTTAAAGAGCTATAGAAATGTCAACCTGGGGGTTCACAAACTTTTTTTTAATTATGATTTTTTCAAAAGAATTTGTTTCTTTTGCAATCCTTT
Md_K224_mRNA  ----------------------------------------------------------------------------------------------------

              5501                                                                                            5600
Hs_KRT224P    GTGGCTCTCTTCTATAATCCCAGCACATTGGGAGGCCGAGGGAGGCAGATCAC-----------------------------------------------
Sh_Krt224     CAAGGTCATCTCCAGTTGTCCTGATCCATATCTTACCACTGGATCCAGATGGCTCTGCAGGAAGAGTAACTTTGCACATCCCTGCTTCATATAAATTTAA
Sh_K224_mRNA  ----------------------------------------------------------------------------------------------------
Md_Krt224     ATATTTTATTTCATGCATTTAAAAAAACATAGTTAGCCTCAGACCCT--------TCCCAGATGTGTGACCCTGGGCAAGTCACTTAACCCCAGGTGTCT
Md_K224_mRNA  ----------------------------------------------------------------------------------------------------

              5601                                                                                            5700
Hs_KRT224P    -----------------------------------------------------CTGAGGTCAGGAGTTCAAGAACAGCCTGGCCACCATGGCAAAACCCT
Sh_Krt224     TTCATTTGCATGTTTTGCATCAT-----CATGATATCATAGTCATCTTCAAGAATGAAGAAATAGTAACTAGGGCAGTGAGGTAGCACAGTGGAGAGAGC
Sh_K224_mRNA  ----------------------------------------------------------------------------------------------------
Md_Krt224     AGCCTTTACCACTCTTCTGCCTTGGAACCAATAGGTCATATTGATTCT-AAGCCTGAAGGTAAGGGCTTAAAAAAACAAAAAAAGTTCTGAGAAAATATC
Md_K224_mRNA  ----------------------------------------------------------------------------------------------------

              5701                                                                                            5800
Hs_KRT224P    GTCTCTACTAAAAATACAAAAATTACCCAGGCTTGGT---------------------------------------GGTGATGCCTGTAATCCCAGCTAC
 Md_Krt224    CCTAGGCTGTCAGAGGGTTCCATTACACAAGCAAGGTTATAGAAGCCCTTTTTAAAAGGTGAGCTCAGAAATGCAAGTTTTACACTTTGTTACTGTATCC
 Sh_Krt224    ACAGGGCTTAAAGTCAAAAATACTCATCTTACTGAGTTCAAATCTGACCTCCTACTAGCTGTGTG---------ACCTTAGTGACTTGAACTCTGTATGC
Md_K224_mRNA  ----------------------------------------------------------------------------------------------------
Sh_K224_mRNA  ----------------------------------------------------------------------------------------------------

              5801                                                                                            5900
Hs_KRT224P    TTGGGAGGCTGAGGCAGGAAAATCACTTGA--ACCCAGGAGGTGGAGGTTGCAGTGAACTGAGATCGCGCCAATGCATCGCAGCCTGGGCAACAGGACAA
Sh_Krt224     CTCAGCTGCCTCATCTGTAAAATAATTGGAGAAGAAAATAGTAAACCATTGCAATGTCTATGCCAAGAAAACCCCACATGGGTCATGGAGAGTTGTACAT
Sh_K224_mRNA  ----------------------------------------------------------------------------------------------------
Md_Krt224     ATTAGCTCATTGAAAAATATTTCCAGTAAATTCGCCAGGAAGGAAAACTTGGAAGGAAAATGAACACTGATATCATCACGTCCACTTGACAAACATTCGA
Md_K224_mRNA  ----------------------------------------------------------------------------------------------------

              5901                                                                                            6000
Hs_KRT224P    GACT--------------------------------CTGTCTCAAAGAATAAATAA--------------------------------ATAAATAAACAA
Sh_Krt224     GACTGAAAACAAATCACAATAAAAGGCTTATTGCCCCTCACTTAGTAACTATTCAATAGATAGATTTTGAATGAATGGATGCAACATTATAAATGTGCAA
Sh_K224_mRNA  ----------------------------------------------------------------------------------------------------
Md_Krt224     GTCCGTGAAATATTTCCATTTGCCATCAGGTGAATATTGTCTTAAGGCTGGAATCCTTACTGGGCTTTGCACTTAGTAGGGCGTTTTCTTTACTC--CAA
Md_K224_mRNA  ----------------------------------------------------------------------------------------------------

              6001                                                                                            6100
Hs_KRT224P    ACTTGAAT-------------------AGCTATGTGTCACTTTTAAAATCAATTTTTCACTTAGGAGCATTCATG-------------------------
Sh_Krt224     AATTATATCTGCGTTAAGGAAACACTGAACAGTGTTCATAATTAATCCACCAGGGTTATCTCTGGGGAAATGGT-----------------------AAA
Sh_K224_mRNA  ----------------------------------------------------------------------------------------------------
Md_Krt224     ACATGAAGCCTTGGTGTGAAAACTGCCTCCACGGATGCAGTTCTGGCATACACGGGTAACTTAGTGGAGGCTTTGTCGAGCGTTAATTCAGTCAGTCAGC
Md_K224_mRNA  ----------------------------------------------------------------------------------------------------

              6101                                                                                            6200
Hs_KRT224P    --ATAAGAATTTATTAAGTGTCCAATGTTTCAGGAGAAGC--------------------------AAAATAGGAACAAGGATGGCAGCAGCATCTTTAT
Sh_Krt224     AATGCACTCTTACTTGAAGGAGAATTGATCTATATAAATTGTTTTTGTGAATT--------TAGTAAATATTAAAAATGCATGTGTAACTTTAACTTCTC
Sh_K224_mRNA  ----------------------------------------------------------------------------------------------------
Md_Krt224     TAACAAGGATTTATTAAAGTGCCAACGAGATATGCCAGGGATGTGTGTTAAGCCCTGGGGATCCAAAAAATAAGAGGGGGTGGGGAGACAGTCCCTGCTC
Md_K224_mRNA  ----------------------------------------------------------------------------------------------------
```

```
              6201                                                                                              6300
Hs_KRT224P    CTTCTGGGAATTACACTTTAGCCATAGGGAAACTTTGATCAAACTAATAATACATAAGGAAGTT---------GCTGTATTTTTCATTGACTTGGTAGGC
Sh_Krt224     TAACAATGGTTTTAAATCAATTTATCTTTTTGCTCTCATAAATTTGCAATTGATATCAGGATGGTAATTGCAAGCATTTGTTAAGCATCCACTCACATGT
Sh_K224_mRNA  ----------------------------------------------------------------------------------------------------
Md_Krt224     TCAAGGAGCTAGAAATCTAATCAGAGAGACAACTCGTACAGGACTAGGTATGTACAATAAATAGAGGGTGTCCCAAGAATCTTAGTGTGCTTTTAAGAGC
Md_K224_mRNA  ----------------------------------------------------------------------------------------------------

              6301                                                                                              6400
Hs_KRT224P    CTGAGTCTCAGTCTTAGGTTGTCTCAAAGCTCACCATGTAACCTTAGATAAGTCATTCCTCTAAAAGTA-------------------------------
Sh_Krt224     CTCAGGTGCCAATGCTCAGAAAACAAAAAATTACTTTAAAATGATGTTTGTCCTCTCAGAAGTGCAATACATCTAAAT----------------------
Sh_K224_mRNA  ----------------------------------------------------------------------------------------------------
Md_Krt224     TTAAAGCTAAATGGTACCGACACTCTAAAATCACACTAAGACTTTTGGGACACCCTTTGACGGAGGAGAAAGTTACTTGCCGCCAGAGGAGCCAGAAAAG
Md_K224_mRNA  ----------------------------------------------------------------------------------------------------
              6401                                                                                              6500
Hs_KRT224P    -TCTATCTCTAAGCCTCATTTTT----------TTCTGCAAAGCAAGGGAGTTGGTGATTACTAAGGTACTTATCGTCTAGCTCTAAAATCTATGACTCT
Sh_Krt224     ---TATATACATAACTAATACTTCATAGG------GAGAGAAACAAAT---------ATCACCGAAAACTTCTTAGTTTTCTTAGAATAAACAGGAAAAT
Sh_K224_mRNA  ----------------------------------------------------------------------------------------------------
Md_Krt224     GTCTCTTGTAAAAGCTAATGGTTGAGCTGAGTCTTGAAGGAAGCCAAGCTGGTGGCAATGAGGGAAAACGTTCTTGCCCAAGGCAAAAATAGAGTCAGAA
Md_K224_mRNA  ----------------------------------------------------------------------------------------------------

              6501                                                                                              6600
Hs_KRT224P    GCAAATAGAAACTGTTTGAATTAAAAAAAAAATCAAAGTGGCAG------AATTAATGTTACAGGGAATTAAAATTAGATCAGATTCACCCTAATTCAAG
Sh_Krt224     ATTAAAATATCCCCGT----GAATTAAAACTAGCATATATACATCCCATAAAGGAGGGGAAGGAGGAAACGAGTTTATTGAGCATTTGCTATATACTAGT
Sh_K224_mRNA  ----------------------------------------------------------------------------------------------------
Md_Krt224     GATTGAGTGCCCCGTGTGAGGAGTAGCAAGGAGGCTAGTGCCACCGATCCAAGAAAGGGCAGCAGGGTGGCACAGTAGATAGAATGTCAGGACTGTTGGA
Md_K224_mRNA  ----------------------------------------------------------------------------------------------------

              6601                                                                                              6700
Hs_KRT224P    CAGTTTCCTGAAACTTTT-------------------------------------------------GAAATGCCTAACATCCAAATGTCAGGTTTTAAG
Sh_Krt224     CACTGTGTTAAATTTTTTAT-AAATGTTATCTCCTTTAATGCC--------CACTGTAAATGCTATAGTTATCCCCACTTTATAACTGAATCAGATAAAG
Sh_K224_mRNA  ----------------------------------------------------------------------------------------------------
Md_Krt224     CAGACTCATCTTCCTGAGTTCAAATCCTACCTCAGATATTTCCTGCACACTCAGTGTCAATATTATAGTTATCCCCATGTTACAGCTGGAGCAAAAAGAA
Md_K224_mRNA  ----------------------------------------------------------------------------------------------------

              6701                                                                                              6800
Hs_KRT224P    GCAGAGTGTCTCAACC------------------------------------------------------------------------------------
Sh_Krt224     ATAAAGGGACTTGTCCAAC----------TAGTGAGTATCTGAGGCTGGATTTGAACTCC----------------------------------------
Sh_K224_mRNA  ----------------------------------------------------------------------------------------------------
Md_Krt224     GTTAAGTGACTTGCCCAAGGTCACCCAGTTAGTGAGGGTCTAAGGTTAGATTTGAACTCTTATCTTCCTAACTCTAGGCCCAATTCTCTATCTTCTGGAC
Md_K224_mRNA  ----------------------------------------------------------------------------------------------------

              6801                                                                                              6900
Hs_KRT224P    ----------------------------------------------------------------TTGGCACTCTAGACATTTTTGGTTGGATAATTCTTG
Sh_Krt224     -----------------------------------ATAACATCCTGAAGAGGAAGAGCAGGATTTTTTTTTCCTGTGCTTTCAGGAGTATGATTTTTTTC
Sh_K224_mRNA  ----------------------------------------------------------------------------------------------------
Md_Krt224     CACTAAAGTTCACTTTTTGTTCATAACATCCAAAGATCCCTCCCTGAAGAGGGAGAGCAACATTTTCT----CCATGCTTTTTGGAGCCTATATTTTCAC
Md_K224_mRNA  ----------------------------------------------------------------------------------------------------

              6901                                                                                              7000
Hs_KRT224P    GGGAGCTGTCCTGTGGGTTTATGGAAGTTTATGGCCTCTACCTAGTAGGTACCAAT----------AGTACACCCCCTACCCCTTATTATGTCAGCCAAA
Sh_Krt224     ACAAAAGATAAATGG------------ACACAGACTTTCACAAAGCACTCAACAAAGAT---AGTAAGTCAGCACATTGGGTCATAAGTTTAAAGCTAGA
Sh_K224_mRNA  ----------------------------------------------------------------------------------------------------
Md_Krt224     ACAGAAAGGAAGATGTATTTTGGCGTGATGAACCCTTCCTTAAAGTACTCAACAAAGATTAAACAAAGTAGGTAAAGTGGATCATAGGTTGAGAGCTAGA
Md_K224_mRNA  ----------------------------------------------------------------------------------------------------

              7001                                                                                              7100
Hs_KRT224P    AATGTCTCCAAATATTGCCAAATG-----------------------------------------------------------AGAGTGGAAGGGAGGCA
Sh_Krt224     ACAGTTTTTAGAGGACTTCTGGTCCAATTCTGCTGTTTTACAGATAAGGAAACTGAGGCCC-----------------TGCCCAAGGTCACA---TGGCT
Sh_K224_mRNA  ----------------------------------------------------------------------------------------------------
Md_Krt224     GGAGTCTTTAAAGGTCTTCTGGTTTAACTCTATTATTTTATAAATGTTAAAACTGAAGTCCACAGAAGTTAAGTGACTTGTCTAGGGTCACACAGTGGCT
Md_K224_mRNA  ----------------------------------------------------------------------------------------------------

              7101                                                                                              7200
Hs_KRT224P    CAAAATCACCTGAAAATCACTGCTCTACCTTAAGAAAGCAGGATTTTCCTACACCATAGGGATGCACTAGGTACAGAAGACAGGCATCCTGAGATACTTT
Sh_Krt224     AGTAAGCATTTGAGAAG-GGACGTTTGTCTCTAAATATAAAGCTCTTTCCACTTCATCATGCTGCAGCAGAATCCCAAAG--------------------
Sh_K224_mRNA  ----------------------------------------------------------------------------------------------------
Md_Krt224     ATTTAGCATCTGAACCCAGGTCCTCTGTCTCCAAATACAAGGTTCTTTCTACTGCATCATACCACAGTAGAATCTCAAAA--------------------
Md_K224_mRNA  ----------------------------------------------------------------------------------------------------

              7201                                                                                              7300
Hs_KRT224P    CCTTCCAGTTAAATCAAAACCTCGCCCTCTTAAAACACAGCATCTGCATAGAAGATTATCTCTGTTTTACTTGAAAGAGAAGAGGTTAGAAGATGAGACA
Sh_Krt224     --TTCC-CCAACCTACAAATTTCTTGTTAATGCAAAAGATG---TGACTC----ATTGTCTTATATAATCATGAAAGATGGTAACAGAACAGAACAAATA
Sh_K224_mRNA  ----------------------------------------------------------------------------------------------------
Md_Krt224     --TTCTACCAACCTGAAAACACGGTGTCAACCCAAAAGGTGATCTGACTC----ACTGTCCTACTCACTCATAAAAGATGATAAAGAACAGAACAGATA
Md_K224_mRNA  ----------------------------------------------------------------------------------------------------

              7301                                                                                              7400
Hs_KRT224P    GTAACATCCATCCAGTGTATATTCCCTTCCTGCAGAGAGAATAGGAAAACTTTCTGATGCTAATCTACCCAAATGACTCTTTATTTCATCTCTTCTCTTA
Sh_Krt224     AGTGATTATCAAAGTGACTTCCTAATTATTTTAAAAGTGAAAATAAAGCATTTTG-ATGTTTATCTCATAGCACAGTGTTTT--------CCTTTCATTA
Sh_K224_mRNA  ----------------------------------------------------------------------------------------------------
Md_Krt224     AATGATTTTCAAAAGACCTTCATAATTATTTAAAAAGTGAAAACAGAGCCCTTTGGATGCTGCTCTCACAGCACAGTGTTTT--------TCCTTCATTG
Md_K224_mRNA  ----------------------------------------------------------------------------------------------------

              7401                                                                                              7500
Hs_KRT224P    GGCAATCTTCGCTCCACTCTCAGAAAACAGCCCTCAAAGCCAAGCTGATGGAATCAAAGGCCAAG----------------CGCCAGCCTCAGGTAGAGG
Sh_Krt224     TATAATTGGTTTCCTTCTATCAGAAAAAATCCCTGGAAGGTAGCTTGTCAGAGACAGAAGCAGGGTATGGAACCCAACTCTCACACACCCAAAACCAAAT
Sh_K224_mRNA  -----------------------AAAAAATCCCTGGAAGGTAGCTTGTCAGAGACAGAAGCAGGGTATGGAACCCAACTCTCACACACCCAAAACCAAAT
Md_Krt224     CACAATTGGTTTCCTTCCCTCAGAAAAAATCCCTTGAAGGCAGCTTGTCAGAGACAGAGGCTGGGTACAGCACGCAACTCTCACAAACCCAAAATCAAAT
Md_K224_mRNA  -----------------------AAAAAATCCCTTGAAGGCAGCTTGTCAGAGACAGAGGCTGGGTACAGCACGCAACTCTCACAAACCCAAAATCAAAT

              7501                                                                                              7600
Hs_KRT224P    TTAGTGCCTGGAGACCCAGCTCCTTCAAGGGCATTGCAAGACCAAGGACCAGAGC-CAGGCTAAATGCAGCTGCTGAAGGTCAAGGCGTGCCTGGTGAAT
Sh_Krt224     CAGTTCCTTGGAGGCCCAGCTCATTCAGATCAGGGATGAAGCTGAGAACCAGAGCACAGAATACAAGCAACTTCTCGACATCAAGACTCGCCTGGAGAAT
Sh_K224_mRNA  CAGTTCCTTGGAGGCCCAGCTCATTCAGATCAGGGATGAAGCTGAGAACCAGAGCACAGAATACAAGCAACTTCTCGACATCAAGACTCGCCTGGAGAAT
Md_Krt224     CAGTTCACTGGAGGCCCAGCTCATTCAGATCCGGGATGAAGCGGAGAGTCAGAGCACAGAGTACAAGCAACTCCTGGACATCAAGACTCGCCTGGAGAAC
Md_K224_mRNA  CAGTTCACTGGAGGCCCAGCTCATTCAGATCCGGGATGAAGCGGAGAGTCAGAGCACAGAGTACAAGCAACTCCTGGACATCAAGACTCGCCTGGAGAAC

              7601                                                                                              7700
Hs_KRT224P    GAAGCTGGGACCAACCACTGCCTGCTCACAGGGCTGAGCTGGTCTGCA-AGAAGCTGTGGGTATGGT---------------------------------
Sh_Krt224     GAAATCGAGACTTATCGCCGCCTGCTAGATGAAGAGGGAGGGTAAGTAGAGAAGCAGATTATTAGG----------------------------------
Sh_K224_mRNA  GAAATCGAGACTTATCGCCGCCTGCTAGATGAAGAGGGAGG-----------------------------------------------------------
Md_Krt224     GAAATTGAGACCTATCGCCGCCTGCTCGACGAAGAAGGAGGGTAAGTGGAAAAGCAGCTGGTCACTCGTTTTAGTCGTGTCCAACTCTTTATGACCCCAT
Md_K224_mRNA  GAAATTGAGACCTATCGCCGCCTGCTCGACGAAGAAGGAGG-----------------------------------------------------------

              7701                                                                                              7800
Hs_KRT224P    ----------------------------------------------------------------------------------------------------
Sh_Krt224     ----------------------------------------------------------------------------------------------------
```

```
Sh_K224_mRNA  ----------------------------------------------------------------------------------------------------
Md_Krt224     TTGGGGTTTTCTTGGCAAAGATGCTGGAGTGGTCTGCCATTTCCCTCTCCAGATCATTTTAAAGATGAGGAACCTGAGGCAAACAGGGTTAAGTGACCTG
Md_K224_mRNA  ----------------------------------------------------------------------------------------------------

              7801                                                                                            7900
Hs_KRT224P    --------------------------------------------------------------------------------AAAGAAGGAGAGTAGTTAGG
Sh_Krt224     ----------------------------------------------------------------------------------------------------
Sh_K224_mRNA  ----------------------------------------------------------------------------------------------------
Md_Krt224     CCCAGGGTCACACAATTAATAAATGTCTGAGACTGCACTTGAACTCAGGTCTTCCTGACTCTATCCACTAGAGAGAGAAAAAATAGACATATTGGGAAAT
Md_K224_mRNA  ----------------------------------------------------------------------------------------------------

              7901                                                                                            8000
Hs_KRT224P    ACTGGGGCACGTATAGGTCTCAATGACTCCTATTTACATTTTTAGCTAACCGCAACAAGGACATA--CAACCAGGGCTGAACAACCTCCACAGGGTTCAT
Sh_Krt224     AAATAGGCATTTCTCTGTCTGGTTGCTCTTTTTTTGCATCTCATTCAAATCAGACTAAGGTTATCTCCATTCTGGGGGGAGCAAATTGCCAGGATGCCAA
Sh_K224_mRNA  ----------------------------------------------------------------------------------------------------
Md_Krt224     AAATAGGCAATTCTCTGCGTACCTGCTTTTGCTTTTCATCTCACTTGAATCACAATGAGGCTATATCTATACCAAGGGGATCAAATTCCCATGATATGAG
Md_K224_mRNA  ----------------------------------------------------------------------------------------------------

              8001                                                                                            8100
Hs_KRT224P    TCAGTAAGGATAATTTTTTTTTAATTTTTTGAGACAGAGTCTCACTCTGTCACC----------------------------------------------
Sh_Krt224     ACTACACAGAAGAATTTCCAAGAGCAATCCAGCATCTTTACTTTATCTTCCCTCATACCCTAAACCT---ATGGGTAAATACTTGATTGTTTATTGGGGG
Sh_K224_mRNA  ----------------------------------------------------------------------------------------------------
Md_Krt224     ACGACACAGAAGAATTACAAAGAACAACATGGCACCCTTACTCTACCTTTCCTTACACCCCAAACCCCAAACAGACCAATGCCTGATGGCCCACTAGGGA
Md_K224_mRNA  ----------------------------------------------------------------------------------------------------

              8101                                                                                            8200
Hs_KRT224P    ----------------------------------------------------------------------------------------------------
Sh_Krt224     CAA-------------------------------------------------------------------------------------------------
Sh_K224_mRNA  ----------------------------------------------------------------------------------------------------
Md_Krt224     CAACTAAACCTGAAATGTCAATTGAAGGGGCAGCTACATGGCTCAGTGGATTGAGAGCCAGACTGTGGGCCAGAGGGAGGGGGGAATTCTGGGTTCAAAT
Md_K224_mRNA  ----------------------------------------------------------------------------------------------------

              8201                                                                                            8300
Hs_KRT224P    ----------------------------------------------------------------------------------------------------
Sh_Krt224     ----------------------------------------------------------------------------------------------------
Sh_K224_mRNA  ----------------------------------------------------------------------------------------------------
Md_Krt224     CTGACATCAGACATTTCCTAGCTGTATGACCCTGGGCAAGTTACTTAACCCTTTATACTTGCCTTTTACCTTATATATAAATGCCTTATACCAATACATA
Md_K224_mRNA  ----------------------------------------------------------------------------------------------------

              8301                                                                                            8400
Hs_KRT224P    ----------------------------------------------------------------------------------------------------
Sh_Krt224     ----------------------------------------------------------------------------------------------------
Sh_K224_mRNA  ----------------------------------------------------------------------------------------------------
Md_Krt224     CTAGTGAATCTGTAAAAGAGAAAAAATCAGATCCAAAATGCAAGGGAACAGCCCAAGCAGGGGAAGGGCAAAGAAGACCCAAGATACCAGAAAGGAACAG
Md_K224_mRNA  ----------------------------------------------------------------------------------------------------

              8401                                                                                            8500
Hs_KRT224P    ----------------------------------------------------------------------------------------------------
Sh_Krt224     ----------------------------------------------------------------------------------------------------
Sh_K224_mRNA  ----------------------------------------------------------------------------------------------------
Md_Krt224     AGGATCTGGGGGAAGTTGAAACTGCCAGATCACTTCTTCTTGTCAGCTCTCCTCCAGCAGATGGGCTGAGAGAGGACCTCCGAGATAGAGGATCCCTCTG
Md_K224_mRNA  ----------------------------------------------------------------------------------------------------

              8501                                                                                            8600
Hs_KRT224P    ----------------------------------------------------------------------------------------------------
Sh_Krt224     ----------------------------------------------------------------------------------------------------
Sh_K224_mRNA  ----------------------------------------------------------------------------------------------------
Md_Krt224     GACTTTGACTACCTTCCAGTGGTCCCCTAAATCTCTCTGTTTCCAGCTGAAGACAAAAAGTAGATTGGGTCTTTGTAGAAAGACATCTACTAAAGAAGAT
Md_K224_mRNA  ----------------------------------------------------------------------------------------------------

              8601                                                                                            8700
Hs_KRT224P    ----------------------------------------------------------------------------------------------------
Sh_Krt224     ----------------------------------------------------------------------------------------------------
Sh_K224_mRNA  ----------------------------------------------------------------------------------------------------
Md_Krt224     TTCTCCCTATCCTCAAAATCGTTCTTGGACTTCATCTCATAGCTAGATCAACTTAGGAGGAAGGACATACCCAACAACTGACCTAAAGGAGGGTCAGACA
Md_K224_mRNA  ----------------------------------------------------------------------------------------------------

              8701                                                                                            8800
Hs_KRT224P    ----------------------------------------------------------------------------------------------------
Sh_Krt224     ----------------------------------------------------------------------------------------------------
Sh_K224_mRNA  ----------------------------------------------------------------------------------------------------
Md_Krt224     GATAGCCTGAACCCCTTGGGATTTGGGAGGGAAGTCAGTTGTCGTCCCATCTCCCACTTTCTTCACCCAACAACCCCATTCTTCCTGTCTTGTGTGTCCC
Md_K224_mRNA  ----------------------------------------------------------------------------------------------------

              8801                                                                                            8900
Hs_KRT224P    ----------------------------------------------------------------------------------------------------
Sh_Krt224     ----------------------------------------------------------------------------------------------------
Sh_K224_mRNA  ----------------------------------------------------------------------------------------------------
Md_Krt224     CAGGAATAACGTATTATCCTTTTAGATCAGGAGTGCCTGTTTTCTAACATCAAGAAAGGGAATTGAAGCTTTGGGGAGAATCTTACCTTTCTCCCCAAGG
Md_K224_mRNA  ----------------------------------------------------------------------------------------------------

              8901                                                                                            9000
Hs_KRT224P    ----------------------------------------------------------------------------------------------------
Sh_Krt224     ----------------------------------------------------------------------------------------------------
Sh_K224_mRNA  ----------------------------------------------------------------------------------------------------
Md_Krt224     AGGAGAATTGGCTCCAGACCAGGGCTGGGGAATATCTCAGGTCTCAAAGGAGAAAGGTGGCAGTTGGGAGACTGGGAAAATCCAGAGGTAGGAGAGTAAC
Md_K224_mRNA  ----------------------------------------------------------------------------------------------------

              9001                                                                                            9100
Hs_KRT224P    ----------------------------------------------------------------------------------------------------
Sh_Krt224     ----------------------------------------------------------------------------------------------------
Sh_K224_mRNA  ----------------------------------------------------------------------------------------------------
Md_Krt224     CCTGCTTCTCAGCAATAGCCCAGATCAAGAAAGGAGGCAATGGGTCAATTCTCTAGGACCCTCTCCTCCAGTCCTTAATCTTCATATTCAACCCAACCTC
Md_K224_mRNA  ----------------------------------------------------------------------------------------------------

              9101                                                                                            9200
Hs_KRT224P    ----------------------------------------------------------------------------------------------------
Sh_Krt224     ----------------------------------------------------------------------------------------------------
Sh_K224_mRNA  ----------------------------------------------------------------------------------------------------
Md_Krt224     TGAGGAGGCATATTCTGTCCCTCTGTAAAACAATTTGTGCTACCTTCCCTAAGGGGAAGAGAGGGTCATTCTTCCACTCTCTCTCTTCAGTTCCCCAGAG
Md_K224_mRNA  ----------------------------------------------------------------------------------------------------

              9201                                                                                            9300
Hs_KRT224P    ----------------------------------------------------------------------------------------------------
Sh_Krt224     ----------------------------------------------------------ACTAAACCAGTTCAAGTCAAGTCAAGAAGTA-----------
Sh_K224_mRNA  ----------------------------------------------------------------------------------------------------
Md_Krt224     TGTCTGTGTGACTTCCTTCTGGTCCTATATTACAATTCTAAGATAAGAGGTATGGGTTAAAAAAAAAAGTCAATTAAAGTCAAGAAGTACAGTTGTGTCT
Md_K224_mRNA  ----------------------------------------------------------------------------------------------------
```

```
               9301                                                                                             9400
Hs_KRT224P     ----------------------------------------------------------------------------------------------------
Sh_Krt224      ----------------------------------------------------------------------------------------------------
Sh_K224_mRNA   ----------------------------------------------------------------------------------------------------
Md_Krt224      CTATATCCATTGATTTGGTATCCAAGGAGTCAATATCTAAATTCAGTTACCCATGCATGTTACCCTGGATAAAATGAAAAAAAAGAGAGACTTGGAAATT
Md_K224_mRNA   ----------------------------------------------------------------------------------------------------

               9401                                                                                             9500
Hs_KRT224P     ----------------------------------------------------------------------------------------------------
Sh_Krt224      ----------------------------------------------------------------------------------------------------
Sh_K224_mRNA   ----------------------------------------------------------------------------------------------------
Md_Krt224      TAAAATGGAATGTTCATATTTTTCAAACCACCCACCAAGCAAGTAGATGCTGTAAAATGTGAATTCCACCAGGTCCAATATATGACCCGCAACCCTTTGT
Md_K224_mRNA   ----------------------------------------------------------------------------------------------------

               9501                                                                                             9600
Hs_KRT224P     ----------------------------------------------------------------------------------------------------
Sh_Krt224      ----------------------------------------------------------------------------------------------------
Sh_K224_mRNA   ----------------------------------------------------------------------------------------------------
Md_Krt224      CTCCATACCCAAGCTCCTATGGGGCACTATCCCCCAATCTGCCTCTAGGGTTCTGGCTAATCGAATTTGTTGTAGCATTATCCCAGTGCCTCTGTTTAAG
Md_K224_mRNA   ----------------------------------------------------------------------------------------------------
               9601                                                                                             9700
Hs_KRT224P     ----------------------------------------------------------------------------------------------------
Sh_Krt224      ----------------------------------------------------------------------------------------------------
Sh_K224_mRNA   ----------------------------------------------------------------------------------------------------
Md_Krt224      AAAAGCCTTATTTACTGTAATAATGCCCCAAAGTACAAGAGAAATAATGTTAGTAGTGCAGTAGTGCCCTCATAACTGCTGATTTGGTAATCTGTCCATG
Md_K224_mRNA   ----------------------------------------------------------------------------------------------------

               9701                                                                                             9800
Hs_KRT224P     ----------------------------------------------------------------------------------------------------
Sh_Krt224      --------------------------------------------------------------------------------------------TTTATCAA
Sh_K224_mRNA   ----------------------------------------------------------------------------------------------------
Md_Krt224      GTTAATAGCAGAAATTCACTTACATCCAAGCTTTCAGCCATCCATTGTAGATCTTGGAACATATCTCCTGAGGATGTGGTATCTCTACTGTGTTTATCAA
Md_K224_mRNA   ----------------------------------------------------------------------------------------------------

               9801                                                                                             9900
Hs_KRT224P     -----------------------------------------------CAGGCTGGAGTGCAGTGGTGCGATCTCCGCTCACTGCAACCTCCACCTCCCGG
Sh_Krt224      AAGCTTATTATGTGCCATGAACTGTGCTAAACATTAGAAATACAGAGCAGGCGTAAATAAAAATATGCAATAAACTAATGCTTCAGTATCCAACTTGCAG
Sh_K224_mRNA   ----------------------------------------------------------------------------------------------------
Md_Krt224      TAGCAGACTATGTGCCATGGACTATATCAAGCACTAAAGGAAAAAAA---GGATAGATAAAAATATATAATAAACTAATATTTCAATGTCCAATTTACAG
Md_K224_mRNA   ----------------------------------------------------------------------------------------------------

               9901                                                                                            10000
Hs_KRT224P     TT--CAAGTGATTCTTGTGCCTCAGCTTCCCAAGTAACTGGGACCAAAGGTGCGCACTACCACACACAGCTAATTTTTTGTATTTTTAGTAGAG-GCAGG
Sh_Krt224      TTTTCAAAAATGAAAATGAAATGGGATTTAAGATTCTTTAGGAATGAATTTCAGCAATTATGAAAAGTTTCTTGTTCTTGTTTTTCTTTTTTAGAGCTGG
Sh_K224_mRNA   ----------------------------------------------------------------------------------------------AGCTGG
Md_Krt224      TTTTCAAATATGAAAGTGGGATGGGTTTTAAGATTCCTTAGGAATGA-TTCCAGCAATTATGAA---------GTTTCTTTTTTTTCTTTACAGCTCTGG
Md_K224_mRNA   ----------------------------------------------------------------------------------------------CTCTGG

               10001                                                                                           10100
Hs_KRT224P     GTTCCGCCATGTTGGCCAGGCTGGTCTCGAACTCCTGACCTCAAGTGATCCACCCGCCTCGGCCTCCCAAAGTGCTGGGATTACAGGTGTGAGCCACCAG
Sh_Krt224      GCTTGAAAACAGAGCTAAGAAAGGTAAGTAACATCT---CTAGGAATTTTCCCTCATGAGAACATTTCTTTCAAGAGAGATTATATATATTAGGTAGAAG
Sh_K224_mRNA   GCTTGAAAACAGAGCTAAGAAAG-----------------------------------------------------------------------------
Md_Krt224      GCTTGAAAATGGAGCTAAGAAAGGTAAGTAACTTCT---CTAGGGATTTTCCCTCATGAGAATATTTCTCCAAATAGAGATTACATATATGAGGTAGAAA
Md_K224_mRNA   GCTTGAAAATGGAGCTAAGAAAG-----------------------------------------------------------------------------

               10101                                                                                           10200
Hs_KRT224P     TAAGGACAATTTTCAATGCCCCATTCCAGCTCTAGGAAAGCAATG-------------------------------------------------------
Sh_Krt224      TGA--AAATTTATAATTAGACAATTACTATATTTACATA-----------------------------------------------------TTAAGTAT
Sh_K224_mRNA   ----------------------------------------------------------------------------------------------------
Md_Krt224      TGA--AGATTAGTAATTATGTAATTATAATAATAAGGCAGCTGGGTGGTACAATGGATATAGTGCCAGACCTGGAGTCATGAAGACTTGAATTTAAATCT
Md_K224_mRNA   ----------------------------------------------------------------------------------------------------

               10201                                                                                           10300
Hs_KRT224P     ----------------------------------------------------------------------------------------------------
Sh_Krt224      GAGCT-----------------------------------------------------------------------------------------------
Sh_K224_mRNA   ----------------------------------------------------------------------------------------------------
Md_Krt224      GGCCTCAAACACTTACTAGCTGTGTGACCTTGGGTAAGTCACTTAACCCAATTGGCCTCAGTTTCCTCATCTGTCAATTGAGCCAGAGAAAGAAATAACA
Md_K224_mRNA   ----------------------------------------------------------------------------------------------------

               10301                                                                                           10400
Hs_KRT224P     ----------------------------------------------------------------------------------------------------
Sh_Krt224      ----------------------------------------------------------------------------------------------------
Sh_K224_mRNA   ----------------------------------------------------------------------------------------------------
Md_Krt224      AACCACTCCAGGATCTTTGCCAAGAAAACCCTAGATTGGATCACAAAGAGTCAGACATGACTAAAATGAAGGTGACAACAACAGTAATTATAATAATAGT
Md_K224_mRNA   ----------------------------------------------------------------------------------------------------

               10401                                                                                           10500
Hs_KRT224P     ------------------------------ACCACGGCCTCGATTTATGAACCCAGCCCCTAGTCCATGCATTACCACCGGCTACAAGGTGCACCACAAA
Sh_Krt224      -------------------------TTCGCAATAATCCATTTAGCATAGTATCTG----ATAGCAGATGGTTTATGTTAGTTATTATTGTTGTTCA----
Sh_K224_mRNA   ----------------------------------------------------------------------------------------------------
Md_Krt224      TCATATTTACAGAGAACACACTTGCTTCACCATAACCCTTTTAGCAGAGTGCCTGGCACATACTAAATGCTATATAATATGAGTTACCGTTGTTATTTTT
Md_K224_mRNA   ----------------------------------------------------------------------------------------------------

               10501                                                                                           10600
Hs_KRT224P     TGTTT-GCCCACCTGAATTGAAATTTAGGCAGAACTGTTGT-------------------------------------TCTGCCAGACCGTCTGTGGAAA
Sh_Krt224      ------GAAGTCATGAGATCAAATCCAGTCTCAGACACTTACTAGACAA----------------------------GTCTGCCTCCATTTCTCTAAATG
Sh_K224_mRNA   ----------------------------------------------------------------------------------------------------
Md_Krt224      TATTAGGAAAATCTAAGTTCAAATGCAGTCTCAAACACTGATTAGTTGTATGACCCTGGGCAAGTCATTTAAATTCTGTCTGTCTTGTTTTCCTCAGTTG
Md_K224_mRNA   ----------------------------------------------------------------------------------------------------

               10601                                                                                           10700
Hs_KRT224P     CAAGTGTATTATATCCAA----------------------------------------------------------CATATTAGATGATAAATAGTCATT
Sh_Krt224      TAAAATGATGATAATCATAGCG-----------------------CCCCACAAGTATTATCAACTTT-----------TTATAGATGAAGTAAC------
Sh_K224_mRNA   ----------------------------------------------------------------------------------------------------
Md_Krt224      TAAAATGAGTATAATCACAGGCATTCCTTTCACACAATGACTTTCCCCATCAAGGTTTCTATATATTGCAGGTCAGCATAATAAATGAAACAAGAATTTG
Md_K224_mRNA   ----------------------------------------------------------------------------------------------------

               10701                                                                                           10800
Hs_KRT224P     TAGGGTGGTGTAATTCAAAAACAGAACGAATAAAACATAGAAAAAGCTAAAATTCAAAGACTTCAACATAGAGCTTTTTAAAAAAA--------------
Sh_Krt224      ------------TGAAACTCAGAGAGTTTAAGTGACCTAGAAGGAGGCTCCTAGGAGTATCTTGGGTCTTCACCCACAGACCCATGTCCTAAGTATTCT-
Sh_K224_mRNA   ----------------------------------------------------------------------------------------------------
Md_Krt224      GGGGGATTTTTGTGGAAGCTACAGACAACATGTGAAGGCCAACAAACAACACAGAAAAAACTTAGAAACTCAGAAATGCATCAAATTCATGTATAGTATT
Md_K224_mRNA   ----------------------------------------------------------------------------------------------------

               10801                                                                                           10900
Hs_KRT224P     ----------------------------------------------------------------------------------------------------
Sh_Krt224      ----------------------------------------------------------------------------------------------------
```

```
Sh_K224_mRNA  ----------------------------------------------------------------------------------------------------
Md_Krt224     GAATCAGCATTTTTAGCTTTTAATTCCATAAATGTACATAATTTCTTTTACTGAAAACCCCCAAAAAAGAAAAAGAAAAATTTCAGACCTCTTCTGTGGT
Md_K224_mRNA  ----------------------------------------------------------------------------------------------------

              10901                                                                                           11000
Hs_KRT224P    ----------------------------------------------------------------------------------------------------
Sh_Krt224     -----------------------------ATCTTCCCTGAACAGGTCCATTCCTGCCCCCAAC-------------------------------------
Sh_K224_mRNA  ----------------------------------------------------------------------------------------------------
Md_Krt224     ACAAAGTGAGGGCCAAAAAATTTTACACAAATTTTCCAGATCACTGGAGCCCCATACCCCTAATCCCTGCAGTATGGAAGGGATACCTATTTTGTCAGCC
Md_K224_mRNA  ----------------------------------------------------------------------------------------------------

              11001                                                                                           11100
Hs_KRT224P    -------------AAAATCTCAGATATTACAATTTTTATGATACCTACAACAATCAAAACCAGA--------------GTATAAGACTATCCTATTTTTC
Sh_Krt224     ---------------AAGCCTTAATCTTAAGATCTAAGTTTTCTCCCCAAACCTCAAAACAGAATTTGTCTTGAAAAGTTGATTAGAAAATATCTTTTTT
Sh_K224_mRNA  ----------------------------------------------------------------------------------------------------
Md_Krt224     TTTTATAGATGAGAAAATCAAGGCTCAAAAAAGTTAAGTGACCTACCCAGGGTCACAGAACTAATACAGATCATACAGGATTAAAACCCAGATCATTTTA
Md_K224_mRNA  ----------------------------------------------------------------------------------------------------

              11101                                                                                           11200
Hs_KRT224P    TTTTCAGTCATAAGTTTGAAACTGGATCAAAAAGAGGAAATAAAATCAAGGATAAGTTCTAAATACAAG-------------------------------
Sh_Krt224     TTTATTTTGTCTGAATGAAATTTTGGGGTGTTTAAGTAGGGCACCAAAATTGCTAGTTATGGCTCCTG--------------------------------
Sh_K224_mRNA  ----------------------------------------------------------------------------------------------------
Md_Krt224     AATTCCAAGTCCAAGGTGCACGTGTCTGAGAGAGAGAGAGGCAGAGAGACAGAGAGACAGAGAGACAGAGAGAGACAGAGAGACAGAGACAGAGAGAGAC
Md_K224_mRNA  ----------------------------------------------------------------------------------------------------

              11201                                                                                           11300
Hs_KRT224P    -----------------------------------------------------------------------------------------------TTTTC
Sh_Krt224     --------------------------------------------------------------------------------------------TGTTTGGG
Sh_K224_mRNA  ----------------------------------------------------------------------------------------------------
Md_Krt224     AGAGAGGCAGAGAGACAGAGAGACAGAGAGAGACAGAGACAGAGAGAGATAAAGAGAGAATGTGTGTGTATATATCTATATGTTTGTGTGTATGTATATG
Md_K224_mRNA  ----------------------------------------------------------------------------------------------------

              11301                                                                                           11400
Hs_KRT224P    TATTATTCTATTTCATTTTCTTCAGAGGAACAGCGTACTCCAAAGTCTAAAAGTGTTGTTTTTATATGAATCCAATGTTTGTATTACCCAGTTAGAGTCA
Sh_Krt224     GATGGTTGT---TTTTAGAGGTGAAACATATAACCAAAACATCGTTTT---AATGGATAAATTAAAGAAAATGAGTATAAAGTAAATAAGTTTCATGATG
Sh_K224_mRNA  ----------------------------------------------------------------------------------------------------
Md_Krt224     TATGGTTGTGTGTTTTAGAGGTAAAACATGTAACCAACACCACCATTTTTCAGAGGATGAATTAAAGAGCATGTGTATAGAGTAGGATTTAGGTGAGAGG
Md_K224_mRNA  ----------------------------------------------------------------------------------------------------

              11401                                                                                           11500
Hs_KRT224P    TAAAATCAAGACTCTGGTTGTGGAATACTTTTAC----------------------------------TGAGACATTTGTCAAAATAGCATTGTAATTTT
Sh_Krt224     AGAGAGCAAGATTTAGGTGATGGGA--------------------------TGGGAGGAGATAAAATTTGAAACTTTGGTCCAGAACAAACAAGAGGTTT
Sh_K224_mRNA  ----------------------------------------------------------------------------------------------------
Md_Krt224     ACAGAAAAAAAAAATGGCACTTGAAACTTTTTTTCAGGAACAGAGAAAGCTTTATTAAACTAGAAAGCATGATGAATTGGGAAAGAAAGAAGCAAGCATTT
Md_K224_mRNA  ----------------------------------------------------------------------------------------------------

              11501                                                                                           11600
Hs_KRT224P    TTT-------------------------------------------------------------------------------------------------
Sh_Krt224     TGTTAGTATTTAAA--------------------------------------------------------------------------------------
Sh_K224_mRNA  ----------------------------------------------------------------------------------------------------
Md_Krt224     ATTAAGCAACTACTATGAGGGGGCAACTGGATGACTCAGGGTATAGCGAGCCAAACCTGGAGATGGGAGGTCCTGGGTTCAAATTTGACCTCTGACACTT
Md_K224_mRNA  ----------------------------------------------------------------------------------------------------

              11601                                                                                           11700
Hs_KRT224P    --------TCTTTCAACTTACACCTAAATAAACAGTAATAAA-------------------TATCTTAGAAGCTATTGCTATATCACATTT---------
 Md_Krt224    CCTTGCTGTATGACCCCGTGCAAGTCAATTAACTCCTATTGCCTAGCTCTTACCACTCATCTGCCTTGGAACCTATACACAGAATTGATTCCAAGATGGA
 Sh_Krt224    ------TCTTTTGTCCAGAGCAAACATAAAAAGTTTTATTAAA---------------------CTTGAAAGATCAATATATAATT------AGGAAGGA
Md_K224_mRNA  ----------------------------------------------------------------------------------------------------
Sh_K224_mRNA  ----------------------------------------------------------------------------------------------------

              11701                                                                                           11800
Hs_KRT224P    ---AAATGGCTTTCCTTCAACATCGTTCACCTCAAAATCAAAAGGTGGAAATGCATTTTAATCCATATTTTCT--TTGAAAGGTCAAAGGAGGTGAAGAA
Sh_Krt224     AGGAAAC----------AAACATTTACTAGGTATCTACTACCG-----GCTAGGTTCTTCAAAAATATTATCTCATGTGATTCTCACAACAATCCCACAA
Sh_K224_mRNA  ----------------------------------------------------------------------------------------------------
Md_Krt224     AGGTAAAGGTTTACTAGAAAAAAGTACCCACTATATACTATGCACTGTGCTAGGCTTTTCATAAACATTATCTTATTTGATCCTCACAATAATCCTGGGA
Md_K224_mRNA  ----------------------------------------------------------------------------------------------------

              11801                                                                                           11900
Hs_KRT224P    GGAAAGGAGATAGACTGAAGAAAGATGCATAATTGAAGACAAAAAGTTAATTAAACAAGAAG--------------------------------------
Sh_Krt224     AGTAGGTGCTGTGATTATTCTC-ATTTTATAGATGAAGAAACTTAGGCAAACAG-GGTTAAGTCACTTGCCCAGGATCACCCAGTTAATA----------
Sh_K224_mRNA  ----------------------------------------------------------------------------------------------------
Md_Krt224     AGTACGTGCCATGATTTTTTTCCATTTTACAGTTGAGGAAACTGAAGTATATAGAGGTTAAATGACTTGCCCAGGGTCACACAGCTAGGTGTCTGAGGTC
Md_K224_mRNA  ----------------------------------------------------------------------------------------------------

              11901                                                                                           12000
Hs_KRT224P    ----------------------------------------------------------------------------------------------------
Sh_Krt224     ----------------------------------------------------------------------------------------------------
Sh_K224_mRNA  ----------------------------------------------------------------------------------------------------
Md_Krt224     AGATTTGAACTCAGATCATCATCATCATAATAGCTAACATTTATATAACACTTACTGACATGCCAGACGCTATGCTAAACACTTTATAATTATTATTTAT
Md_K224_mRNA  ----------------------------------------------------------------------------------------------------

              12001                                                                                           12100
Hs_KRT224P    --------------------------------------------------------------------------------------------GTTTCAAC
Sh_Krt224     ------------------------------------------------------------------------------------------AATATACGAGG
Sh_K224_mRNA  ----------------------------------------------------------------------------------------------------
Md_Krt224     AATATTTTAAAATTTTAATTTATTTTAAATTAAAATTAAATTTTAAATTAAAAAAAATATTTAAGTGACTTGTCCAGAGTCACCCAGCTAGTGTCTAAGG
Md_K224_mRNA  ----------------------------------------------------------------------------------------------------

              12101                                                                                           12200
Hs_KRT224P    TTGTAGGTTAGTTTAAGGCAGTGTTTTTCAAGCTGCAGGCTGCGATCCATTTACTTATGTATGTGTGTATGTATGTATG---------------------
Sh_Krt224     CTGGATTGGAATTCAGGTATTCCTGACTCCAGGCCTATGGTTCTGTCCATTATGCCACCTAGTTGCTAAAGAATCATTGGCATTTCTTCATCAGTCGGTC
Sh_K224_mRNA  ----------------------------------------------------------------------------------------------------
Md_Krt224     TTGGATTTGAATTCAGGTCTTCCCAACTCCAGGCCCAGGGTTCTATCTACTATGACACCTAGCTGCTGAGGAATCCTTGGCATTTCTTCATAAGTTGTTC
Md_K224_mRNA  ----------------------------------------------------------------------------------------------------

              12201                                                                                           12300
Hs_KRT224P    -------------------------------------TATGTATGTATTTATTTTTGAGACAAGGTCTCACTCTGTCACCCAGGCTGGAGTGCAGTGGTG
Sh_Krt224     TTACTACTGTGTA-----------------------CTACGTGCTAATTTGTCTTTCACCTAAGCTCATATTTTCTGAACAGTGGGAGAACGCTGAATT-
Sh_K224_mRNA  ----------------------------------------------------------------------------------------------------
Md_Krt224     TTATTAACTTCTTTCTTGATTGTCATTTCACCTTTTCTGCATGCTAATTTGCCTTTCATTTAACTCCACATTCTCAAGACAACTGCA--------AATT-
Md_K224_mRNA  ----------------------------------------------------------------------------------------------------

              12301                                                                                           12400
Hs_KRT224P    CAATCTCTGCTTACTGCAACCTCTGCCTCCGAGGTTCAAGC-CATTCTCATGCCTCAGCCTCCCAAGAAGCTGGGATTACAGGTGTACTGGGCCATGATG
Sh_Krt224     -------TTCTTGCTCTGCTATTTGCCCCTTGGCTTCATTCTTACTTTGATGAGTATGAGAACATCTGTCTTCCCACCAACTGTCTCTTTTCAGCCTGAG
Sh_K224_mRNA  ----------------------------------------------------------------------------------------------------
Md_Krt224     -------TTCTTGCTCTGCTATTTGCCCTTTGGGTTTGTTCTCATTTTGATGAGTATGAGAACATCTATCTTCCCACCACTCCTCTCTTCCCAGCCCTGT
Md_K224_mRNA  ----------------------------------------------------------------------------------------------------
```

```
                12401                                                                                            12500
Hs_KRT224P      TAAAATGATGCCTTTGTTCTTGTGGCTTGCAGTGGGAAAAGTTACAGCCTTTACTATAGAGTTTTCTTTATTTTTCCTTTCAGTTTTTACT---------
Sh_Krt224       GAACTTCATACCCTGCCTCCCAGTCATAGGGAATTTTAAAATGTTTTACATTTCTCTGAATTATACTCTCCCAATTCTTTTATGCCTTTCTAAGTAGATC
Sh_K224_mRNA    ----------------------------------------------------------------------------------------------------
Md_Krt224       GAATTTCATACTCTTCTTCTTCATCACTGGGAGTTTTAAAATGCTTTACATT-CTCTGAATTATCCCCCCACCCCCCACC-------------------C
Md_K224_mRNA    ----------------------------------------------------------------------------------------------------

                12501                                                                                            12600
Hs_KRT224P      -----TGAACTTAATTGTATGAAATGGATTGTGG----------GCCAAGCGTGGTGGCTCATGCCTG----------------TAATCCCAGCACTTTG
Sh_Krt224       CCATCTATCTTGAATTCTATTAAGTGACTATCTGTTCCCATACATACACACTCCTATTCTGCTGCCTGCTGACTCTAATTTTCCTCCTTCTGACACTTGG
Sh_K224_mRNA    ----------------------------------------------------------------------------------------------------
Md_Krt224       CAGTCCTTTCTGAATTCTATTAAATGGCTATTTGAGTCCTCCTAGCCTGTCCTGGACTCTAGCTCCTC--------------------TCTGACCCTTGG
Md_K224_mRNA    ----------------------------------------------------------------------------------------------------

                12601                                                                                            12700
Hs_KRT224P      GGAGGCCAAGGCAGGTGGATCACCTGAGGTCAGGAGTTCGAGACCAGCCTAGCCAA--------------------------------------------
Sh_Krt224       AGAAGTATTCCCTGCTGTGCCCCCGCCCTTGAGGATGAATTTCTTATACTTTCCACACCTAGTTTCTGGACTAGTTTCATCCATTAAAAATGACGTTTTG
Sh_K224_mRNA    ----------------------------------------------------------------------------------------------------
Md_Krt224       TCCAGTATTCTCTATTGTGCCCTTTCCCTGGGATAGAGGAGGATTCTC-------------------------------TCTCTTATTACTGTGCACACT
Md_K224_mRNA    ----------------------------------------------------------------------------------------------------

                12701                                                                                            12800
Hs_KRT224P      CATAGTGAAACCTCGTCTCTATTAAAAATACAAAAATTAGCTGGGCATGGTGGTGCATGCCTGTAATCCCAGCTACTCGGGAGGCTGAGGCAGGAGAATC
Sh_Krt224       CTTATATGTTTTGCCTCTAAAACTAGTTTATACAGATAGACCATGTTCTGTTCTTTGTTTTTGAAATCCCTCTCTCATAGGAATTTTTTTTTCTGTGATT
Sh_K224_mRNA    ----------------------------------------------------------------------------------------------------
Md_Krt224       CATCATCGTTTGGGGGCTAGTTGGACATG--------------TGTGACCCTCTCAACCTTTGAAATCCCAACCCCACTGGAAATTCACTTTGGCTACTT
Md_K224_mRNA    ----------------------------------------------------------------------------------------------------

                12801                                                                                            12900
Hs_KRT224P      ACTTGAACTCTGGAGGAGGAGGTTGCAGTGAGCCGAGATCACACCACTGCACTCCAGCCTGAGTGAC---AGAGTGAGACTCCATTGAAAGAAAGAAAGA
Sh_Krt224       A--TGGACAGATATGGGGGAGTTTGACCATCTGCCTAGTGACTTTCTTGAAAATCAATTTTGGTCTCCTTAGATGTTTCTTACATACAAATAGCCTAGAA
Sh_K224_mRNA    ----------------------------------------------------------------------------------------------------
Md_Krt224       A-------------------GTGTGCAAATCTACCTGGTGATTTTCTTAAAGATCA-TTTTGCTATCCCTAAATGTTGCCTGCATATGAATAGACTAGAA
Md_K224_mRNA    ----------------------------------------------------------------------------------------------------

                12901                                                                                            13000
Hs_KRT224P      AAGAAAGAA-------------------------------------AGAAAGAAAGAAAGAAAGAAAGAAAGAAAGAAAGAAAGAAAGAGAAAGGGATTG
Sh_Krt224       AAGATTGTG-----------------------------GTAGCATGATGCAAAGCCAATCGAAGGAAAGAGGAGAATACAATAAAAATTTCAGGAGATCA
Sh_K224_mRNA    ----------------------------------------------------------------------------------------------------
Md_Krt224       AAGACTGTATCATGTATCATAAAAGATGTATCATGATTGTATCATGAAAATAAGACAATGGAAAGAGAAAACAGAACACAATAAAAATCTTAGGAGTTCA
Md_K224_mRNA    ----------------------------------------------------------------------------------------------------

                13001                                                                                            13100
Hs_KRT224P      TGAATCTGTTTGC----TATTGTTCTTGATGTCACTTCCTTCTCTATGACTGAGAATTCACTGGCA-------TTCCTAGAAGAATTCAAACCCTTCATC
Sh_Krt224       TGGAATAGTAAAAAGAGTATTGCACT-GGAATCACAGGAACTAGTTAGAATATTAACTTTGCTACTTATTTTCTGTGTTGCCTTAAGCAAGACGCTTAAC
Sh_K224_mRNA    ----------------------------------------------------------------------------------------------------
Md_Krt224       TGGAATGATAAA--GAGTATTGAACTTGGAATTCCAGGAACCCATTAGAATATCAACTCCAC----------CTGTGTTGCCTTAAGCAAATTACTTAAC
Md_K224_mRNA    ----------------------------------------------------------------------------------------------------

                13101                                                                                            13200
Hs_KRT224P      GTTTGTTGAGCTAATTTCACTATGATTTCACCATATGCCACTTTCTTGCCTATATCATTGCAATGCCTCTCTACATAGGACTTGCTGTATTATAACAATG
Sh_Krt224       ATCTCTGTACCTCAGTACAGAGGTACTCTTCATTTGTAAAATGGTTGAACTAAACAAA---CTAGATAACCACCATAGATC--------TAAAGGCTAAA
Sh_K224_mRNA    ----------------------------------------------------------------------------------------------------
Md_Krt224       ATCTCTGAGCCTCAGTTC---------CCTCATCTGTAAAATAGTTGAACTAGATTCG---ACTGCCCGGGATCTATGACTATGATCCTTGAAAGCTAAA
Md_K224_mRNA    ----------------------------------------------------------------------------------------------------

                13201                                                                                            13300
Hs_KRT224P      ATAACAAAGACAACTACCATTTTTGGAGCACCCACTATGTAAACATAATTCCCACAGCAATTCTGTAGATGGGGCATGATCCCCATTGCATAGATG----
Sh_Krt224       ATAATAAGACAAGGGAACTGATTGGAGATGAAAAAACACAAGTGAGGAATTGAAAAAAAATTTTTTGTAATGACTCCGATTTAGACAATAGCAATGGTAA
Sh_K224_mRNA    ----------------------------------------------------------------------------------------------------
Md_Krt224       ATGACAAGACATGGGAACTGA---GATATAGGAAAACAGAGGCAAGGAGTCAAAGAAAATTCTAATATTTTGTGAATGATTT------------------
Md_K224_mRNA    ----------------------------------------------------------------------------------------------------

                13301                                                                                            13400
Hs_KRT224P      ----------------------------------------------------------------------------------------------------
Sh_Krt224       TAACAGGCAAAATGGGAAGGAAAGCTGATTTGCAAATGAAGATAATGAAGTAAATTTTGGACATGTTGAATTTAAATGACATTGGCTTTAGAGGCAAAAG
Sh_K224_mRNA    ----------------------------------------------------------------------------------------------------
Md_Krt224       ----------------------------------------------------------------------------------------------------
Md_K224_mRNA    ----------------------------------------------------------------------------------------------------

                13401                                                                                            13500
Hs_KRT224P      ---------------------------------AGGAAACTGAAGTTCAGCGAGGTTAAATAGCTCACCCATGATCACAAAGCTCATCTGGGACA-----
Sh_Krt224       TTCTAGGTTCAAGTCCCAGTTTTAATATTTACTACGTACATGAGTTTGAACAAATCATTTAACTTCTCTAGGATTCAATTGGCTCCTCTGAAAAATGAAG
Sh_K224_mRNA    ----------------------------------------------------------------------------------------------------
Md_Krt224       ------------------------------------TTGTTCATTTTCATCCAGTCATTT----------------------------------------
Md_K224_mRNA    ----------------------------------------------------------------------------------------------------

                13501                                                                                            13600
Hs_KRT224P      ----------------------------------------------------------------------------------------------------
Sh_Krt224       AAATTGGACTAGATAATTTCTAAAACCTCTTGTAAATCCTATGATCAAAATGGAAATACCTGACTGAAGATGGAAACTTAAACCTATTAACTATAAGATA
Sh_K224_mRNA    ----------------------------------------------------------------------------------------------------
Md_Krt224       ----------------------------------------------------------------------------------------------------
Md_K224_mRNA    ----------------------------------------------------------------------------------------------------

                13601                                                                                            13700
Hs_KRT224P      -----------------------------GAGTAAGCCAGGATCAAACCTTTTGAGGTTCCAAAATTCCAAGTGATAATCTCATTCCCATTTTTCATCCT
Sh_Krt224       ACATCTGTATTTTATTGTTGTTCAGTTATGTCTAACTCTTCATGACCCCATTTGGGGAGTTTATTGGCAAAGATACTGGAACAATTCATCATTTCCTTCT
Sh_K224_mRNA    ----------------------------------------------------------------------------------------------------
Md_Krt224       ---------------------TCAGTCATGCCCAACCCTTCATTGATCCCATTTGGGTTTTTTTTGGCAAAGATACTGGAGTGATTTGCCATTTCCTTTC
Md_K224_mRNA    ----------------------------------------------------------------------------------------------------

                13701                                                                                            13800
Hs_KRT224P      CTCAACACTTTATGAAGTGAGTACTATTATTATCCCCAGTTTACA-------------GATAAGGACACTATAATTTTCCCAAGTTATACAGTAACTTGG
Sh_Krt224       CCAGCTCATTTTATAGATGAAGAAACTGAGGGAAATGGGGTTAAGTGACTTCCCAAGGGTTACACAGCTAGTAAGTGTCTGAGATTAGACTTTAACTCAG
Sh_K224_mRNA    ----------------------------------------------------------------------------------------------------
Md_Krt224       CCAGCTTTTATTACAGATGAGGAAACTGAATTAAACAGGGTTAAGTGACTTGCCCAGGGTCACCCATCTAGTAAGTATCCGACATCAGA-TTGAACTCAG
Md_K224_mRNA    ----------------------------------------------------------------------------------------------------

                13801                                                                                            13900
Hs_KRT224P      GAAGAGTTAGGATTCAAACCCTGAATCAAGCAGTATGTTCTTTTTCGTGTCTGGCT--------------------------------------------
Sh_Krt224       GAAGAGGAGTCTTCCTAACGTCAGGCCCAAAGTTCTGTTCATTATGGTGCCACACC------------------TAGCTGAACAAATATCTTTGTTGTGT
Sh_K224_mRNA    ----------------------------------------------------------------------------------------------------
Md_Krt224       GAAGATGAGTCTTCCTGATTCCAAGCCCAGTACTCTATCCACTATGCCACCTAGCTGCCTGAACCTAACAGCTATAAAATAGCATCTTTATTGGTTATGT
Md_K224_mRNA    ----------------------------------------------------------------------------------------------------

                13901                                                                                            14000
Hs_KRT224P      --TCTTTCCCTCAACACGCCGTGTGCGCGATTCGTCTGTGCTACTGAT------GTGGTTGTAGATCATGTTATGCCTTTAATAAACACCTGTGTTAAGT
```

```
Sh_Krt224      ACTTTTTATGTAATTGCTCTTTGTTGGAGTTTGTTTTTTAATCTTGCTAACAGG--ATTTGGAGCTAGAAGTAATGAGTCCAAGATCATATATGTAGTAA
Sh_K224_mRNA   ----------------------------------------------------------------------------------------------------
Md_Krt224      ACTTTTTGTGTAATTTGGGGTTTTGT---TTTTTTTAAGGATCTAGCTAAAATGGAATTTAGAGATAGAAATTACTTGTCCAAAGTCACATATGTAATAA
Md_K224_mRNA   ----------------------------------------------------------------------------------------------------

               14001                                                                                          14100
Hs_KRT224P     GTTGGAGATACAAAGATGTGGTCCCTCTCTCGGAGAATTTACAGTCT------------------------AGAAGGAAAAATATATGCAAACATGTAGC
Sh_Krt224      ATGGCAGAACCCATGATTTGAACTTTACCTCTAATCCAGGACATTTTTCTACCATACCTCATCATTTCTCCAACATGATATGTGCATCCATAACAGAATA
Sh_K224_mRNA   ----------------------------------------------------------------------------------------------------
Md_Krt224      GTTGCAGAACCCAGGATTTGAATTTTAACTCTAATATAGGGTATTTTT--------------------TCCAACAGGACATATGTATCAAAATGA-AATA
Md_K224_mRNA   ----------------------------------------------------------------------------------------------------

               14101                                                                                          14200
Hs_KRT224P     TAACAT---------------------------------AAAGTATTAACTACAATTCTGACTGAATTTTATCAAAGTCAC----TCCAAATTTGTGAAG
Sh_Krt224      TAAAATTCCTTGTAGCTGTAGTAGTTGTTTTTCCTCATTAAATTCA--ACTTAGATGCTGACAAAATTCTTTCCAACTTGCATAATTTAAAGTAATC---
Sh_K224_mRNA   ----------------------------------------------------------------------------------------------------
Md_Krt224      TAAAGTTCATTGTACCTATAGTAGTTGCTTTTATTATTAAATTCAATAACTTAAATGCTGGCAAATTTCTTTCTAACCTACATAATCTAAAATAATCTAA
Md_K224_mRNA   ----------------------------------------------------------------------------------------------------

               14201                                                                                          14300
Hs_KRT224P     CTTTAGAAAAGCAAAGATTAAAATCAATGAGATTTATA--------------------------------------------------------------
Sh_Krt224      ------------ACAATCTAAAATGATTGTCTGGTTTTAAAATTAGTTCCCATCCCAGCTGTTTCTT-------------------------TTTCTTTA
Sh_K224_mRNA   ----------------------------------------------------------------------------------------------------
Md_Krt224      TCTAAAAAAATCACAATCTAAAATGATTG---GTTTTAATAATTGGCTCTCATCCTAGCTGTTTCTTCTTCCTTGGGCTTTAAGCAAAATAGTTTCTTTT
Md_K224_mRNA   ----------------------------------------------------------------------------------------------------

               14301                                                                                          14400
Hs_KRT224P     ----------------------------------------------------------------------------------------------------
Sh_Krt224      GCTGCTGAAGTCTTTTCAG---------------------------------------------------------------------------------
Sh_K224_mRNA   ----------------------------------------------------------------------------------------------------
Md_Krt224      TCTCCCTAAGAATTTTCCAATTTTTAGCTGCCCTAAGTGTCCCCTCTAGACAAAATATAAAAATTATTGATTTCATCAATATTGATTATCAGCTAAATTG
Md_K224_mRNA   ----------------------------------------------------------------------------------------------------
               14401                                                                                          14500
Hs_KRT224P     ----------------------------------------------------------------------------------------------------
Sh_Krt224      ----------------------------------------------------------------------------------------------------
Sh_K224_mRNA   ----------------------------------------------------------------------------------------------------
Md_Krt224      TATTTTTAAATGCTCCTTTAATTCAACTTTTTAATCACTTATAAACAGTACACTGGTAAATGTTTAACAATCAGCTCTCTGGAAAAAAAATTGTACACAT
Md_K224_mRNA   ----------------------------------------------------------------------------------------------------

               14501                                                                                          14600
Hs_KRT224P     ----------TGCTTATCTTTCTCACTGTTGAAAGTAGGTAGAAAAGTT---------------------------------------------------
Sh_Krt224      ---------TCATGTCTGACTCTCCATAACTTCATTTGGGGT-----TTTCTTGCCAAAGTTAC------------------------------------
Sh_K224_mRNA   ----------------------------------------------------------------------------------------------------
Md_Krt224      AACACACTTTTAAGTGTAATTCACATTATTTACATTTTCTCCATCACTTTCTTGTCTAAGTTATCCAAAAAAACAAGCCCTGATTTGTAGCAATCAACAA
Md_K224_mRNA   ----------------------------------------------------------------------------------------------------

               14601                                                                                          14700
Hs_KRT224P     ------------------------------------------------------------------------------------------AATAAGTACC
Sh_Krt224      ---------------------------------------------------------------------------------------TGGAATGACTTGC
Sh_K224_mRNA   ----------------------------------------------------------------------------------------------------
Md_Krt224      TTTCTAAGGCATTAATGCTCACACTAAAAAAATTTAATAATTGGCTCCTTGGAAGCCTATTCAAATTGGCTTCAGCTTTCCCTTGATTGTTATTATTAGT
Md_K224_mRNA   ----------------------------------------------------------------------------------------------------

               14701                                                                                          14800
Hs_KRT224P     CAATACGAAAGCTGTCTTACTTTTGATTAAAGAGAAATG-------CTTTATCAATTTACTTTATTTCCATCAAT-------------------------
Sh_Krt224      CATTTCCTTCTTCAGCTTATTTTACAGATGAGGAAACTGAGGCAAGCAGTGTTAAGTGACTTGCATAGCCTAACTCTCCCT-------------------
Sh_K224_mRNA   ----------------------------------------------------------------------------------------------------
Md_Krt224      GATAACAATAATAATATTGGTTAGCAGCATTGGAAAATGGGGCTTTTTTACTTCCTCAACATGGATAGTGAAAATCAAGCACTGCAGTTAAGAGTACCTA
Md_K224_mRNA   ----------------------------------------------------------------------------------------------------

               14801                                                                                          14900
Hs_KRT224P     --------------------ATGTAATTGACCTAA---------------------------CACTTTCTTTTCGCAGAACCAACGAAATGTTTTGGCAC
Sh_Krt224      --------TCCATACTTTTGCTCAGGTTGAACTCAGATATTCC-----------------------TTCTTCATCTCCACCTTTTAGAATACCTAGTTTC
Sh_K224_mRNA   ----------------------------------------------------------------------------------------------------
Md_Krt224      GGTTTGAATCTTGGCTCTTCACCTGTGTGACCTTAGGAAAATCACTTAATCTTTTTGGACCTCAGTTTCTTTGTCTATAAAATAGATATTTCTAAGGTCC
Md_K224_mRNA   ----------------------------------------------------------------------------------------------------

               14901                                                                                          15000
Hs_KRT224P     AACACAATTGTTAAAGAACTGATT----------------------------------------------------------------------------
Sh_Krt224      CTTCAAAGTCCTATGGGCATGCCAGC--------------------------------------------------------------------------
Sh_K224_mRNA   ----------------------------------------------------------------------------------------------------
Md_Krt224      CTTCTAAGTTCTAAATTTAGGGATTCACAGATTCCTCTCACTTGTCACTTTCATCATTCCTGGGTTTTATGCTGAGTAGCAGACTGAAAACTAAGAACTT
Md_K224_mRNA   ----------------------------------------------------------------------------------------------------

               15001                                                                                          15100
Hs_KRT224P     -----------------GATGGCATTGTTGTTTTATC---------------------------------------------------------------
Sh_Krt224      ---------TTTTAGATGAAAGCTTTCCTGGTCCTTCC--------------------------------------------------------------
Sh_K224_mRNA   ----------------------------------------------------------------------------------------------------
Md_Krt224      CCAGAGGATTATTCTAGGAAAGCCATTTTCTCCTCTCCTCCATATTAAAGGGTGTACATGAATACAAAATACAGTACCTGCCAAAAATCCATATTCTATT
Md_K224_mRNA   ----------------------------------------------------------------------------------------------------

               15101                                                                                          15200
Hs_KRT224P     --------------------------------------------------------------------------------------------ACAAATAA
Sh_Krt224      --------------------------------------------------------------------------------------------AGATATTA
Sh_K224_mRNA   ----------------------------------------------------------------------------------------------------
Md_Krt224      ATGTCAATCTTATCTATTAATTGAAGTAAGGATCTCTACTTGGAGGGCAGCTAAGTAGTGTAGTGGATAGAAAGCTAGGTCTGGAGTCAGGAAGACTTAA
Md_K224_mRNA   ----------------------------------------------------------------------------------------------------

               15201                                                                                          15300
Hs_KRT224P     GAGGTGTTCACCAAAGACCAGTT-----------------------------------------------------------------------------
Sh_Krt224      CTGCCCCCTTCCAAAATTACTTTATTTTTTACT-------------------------------------------------------------------
Sh_K224_mRNA   ----------------------------------------------------------------------------------------------------
Md_Krt224      CTTCCTGAATTCAAAGACACTTATTAGTTGTATGATCCTGAGCAAATCATGCAACCCTGTTTGCCTCAGTTTCCCCATCTTTAAAATGATTTAGAGAAGG
Md_K224_mRNA   ----------------------------------------------------------------------------------------------------

               15301                                                                                          15400
Hs_KRT224P     ----------------------------------------------------------------------------------------------------
Sh_Krt224      -----------------------------ATACTTCTATGCATATGA-----------------------------------------------------
Sh_K224_mRNA   ----------------------------------------------------------------------------------------------------
Md_Krt224      AAATGGCAAACAACAACAACCTAATCCTAATTATTCTATGCATTTGAGAGTAATAAAATGGTATGTACTTTAAAACCACACTATGAGGACAGTTAGGTGG
Md_K224_mRNA   ----------------------------------------------------------------------------------------------------

               15401                                                                                          15500
Hs_KRT224P     ----------------------------------------------------------------------------------------------------
Sh_Krt224      -TCATTGGATCATAGATTTAAA------------------------------------------------------------------------------
Sh_K224_mRNA   ----------------------------------------------------------------------------------------------------
Md_Krt224      CTCAGTGGATTCTGGGTTCAAATTTGACCTCAAACATTTCCTTGCTGTGTGACCCTGAGCAAGTCACTTAACCCCCATTGCCTAATCCTTACCACTCTTC
Md_K224_mRNA   ----------------------------------------------------------------------------------------------------
```

```
              15501                                                                                              15600
Hs_KRT224P    --------------------------------------------------------------TAAGGAGCACCACACAAGCTAGCCACAAAAAC------
Sh_Krt224     ------------------------------------------------------------AATAGAGAGGATCTTTGAAATCATTTAATCCAAAATGTGA
Sh_K224_mRNA  ----------------------------------------------------------------------------------------------------
Md_Krt224     TGACTTGGAACCAATTCATAATCACAATTTTAAGACAAAAGGTAAGGGTTATGAAAAAAAAGTAGAGAAGACCTTAGAAACCATCTAATCCAAACTATCA
Md_K224_mRNA  ----------------------------------------------------------------------------------------------------

              15601                                                                                              15700
Hs_KRT224P    TAAGACAAATAAAGCACCAAGAACCATTTAACCTTGGTCACGTATATTGGATAACTGAAGCAATGTATAGAGTGAAACTTTGCAAAAGCCATGAAGACAA
Sh_Krt224     TTTTGCAAATAAGGAAACTGAGTCCCAGGAAAGTTAAGTGATTTGCCCAGAGTCACATGGTAGCAAAGATAGTATTTGATTTCATAGTGTCTGACTCCAA
Sh_K224_mRNA  ----------------------------------------------------------------------------------------------------
Md_Krt224     TTTTACAAATAAAGAAACTGAGTCCCAGGAAAGTTAAATGACTTCCCCAATGTCACACTGTAGCAGAGATAGTATTTGATTTTATATCCTCTGACTCCAA
Md_K224_mRNA  ----------------------------------------------------------------------------------------------------

              15701                                                                                              15800
Hs_KRT224P    GGGAATGTATTTCAACAATACTCATCTTTTTACACTAGGAAAAAT-------------------------------------------------AAAAGC
Sh_Krt224     ATTCATTCATTTTTCCATGGTTCATGTTGTCTCCAGCCATAAAATGTAAATTCCTTGAAGGCAGAGACTGTATCCAGAATCTAG-----TGCAAAACAAG
Sh_K224_mRNA  ----------------------------------------------------------------------------------------------------
Md_Krt224     ATTCATTGATCTTTCCACTGTTCCTGTTGTCTCCAGTCACAGTATGTAAATTCCTTGAGGGAAGAGGCTGTTTTGTTTTTGTCAGCGCCTACCACATAAG
Md_K224_mRNA  ----------------------------------------------------------------------------------------------------

              15801                                                                                              15900
Hs_KRT224P    CAGCAGGATATTTAGGGAATGTTTTTGCTCATCTTCAGTAATGT------CATGTGAATGCACCCATCTACATTTTGGAATTTTTTAATCTTTGTGGACT
Sh_Krt224     CCAATAGACACTTAATAAATATTTTT-GAAGGATTAATTGATTTATTATGCAATTAAAACCAGTAATTTTCATGCCAGGACTAATAGCGTAGGACATACT
Sh_K224_mRNA  ----------------------------------------------------------------------------------------------------
Md_Krt224     CCTATAGACACTTAATGAATGTTTTTTGAATGAATAATTGATGCATTACTCAGTCAAAACTAGCAATTTTCATGCCAAGACAAATAGCACGAGATATACC
Md_K224_mRNA  ----------------------------------------------------------------------------------------------------

              15901                                                                                              16000
Hs_KRT224P    TAACAAAAATGAGTACACTTCTAATTCAGAAGGTCTAACCTTGCAGTTTTTAGCTCTGGTCTGAAGCAATCAATTTGGGTCCAG----------------
Sh_Krt224     CATGAGAAGTGGTATAAAATACACCCTAAAAAGCATGTCGGAGAACCCATTACACAAGGTGGG----AAATAGCCAGGGAACAGCTTACCTGGGGGGAGG
Sh_K224_mRNA  ----------------------------------------------------------------------------------------------------
Md_Krt224     CAGAAGAAGTGACATGAAATACACCCTAAAAA-CATGTTGGGGAGGCCATAACATGGGGTGGGCAACAAACAGCCAGGAAACAGCTTGCCTGGGGAA---
Md_K224_mRNA  ----------------------------------------------------------------------------------------------------
              16001                                                                                              16100
Hs_KRT224P    ----------------------------------------------------------------------------------------------------
Sh_Krt224     GTGGCTTCTGGAGGAAGGAAGTAGGCAGTTTGACAGATTGTAATTTTTCTTAATTTTTTTCTTGCTAAGTAGGTGCTAAGTTCAGTTATTTCCA------
Sh_K224_mRNA  ----------------------------------------------------------------------------------------------------
Md_Krt224     -TGGATCCAAGGGGAAGGAAGTAGGTAACTT------------------------------TTGCTAACTAGGTGCTAAGCTCAGTTATTTCTATACTTG
Md_K224_mRNA  ----------------------------------------------------------------------------------------------------

              16101                                                                                              16200
Hs_KRT224P    ---------------------------------------------------------------TGCTTGGCTAAGCACTCCAGACTCCAGACAGTAAACA
Sh_Krt224     ----GAACTCCAAATATTCTAATTACCCTCTAAACTTAAATTCCCTAGAATGAAGCTTTGATCTACCTTACTTATAGTTCTGACATCTTTCTATAAAATA
Sh_K224_mRNA  ----------------------------------------------------------------------------------------------------
Md_Krt224     TAAAGAATTCCTAGTATTATAATCACCCTCTAAATCTAAATGCCCTGGGATGAAGTTCTCTTCTGCCTTAGTTTTAACTCTGGTATCTTTCTGCAAAATT
Md_K224_mRNA  ----------------------------------------------------------------------------------------------------

              16201                                                                                              16300
Hs_KRT224P    ATAAGAGACAAATATTCCCTTCTAGAAGGTACAGCCCATCTGGAGATGTGGGAGATAC-----------------------------------TCTTGAA
Sh_Krt224     ACTAAAAATCAAATTAACT--GAATTTTCTGCTTAAAAATTTAATAAATATAAAATATACAGACTTTTCACTTCAGTCTTGGACAAAGACATGTCTTTTG
Sh_K224_mRNA  ----------------------------------------------------------------------------------------------------
Md_Krt224     AGCAAAGATCAAAATTAATTGGATTTTTCTGCCTAAAAATTTAGTAGGCATAAAATATGTGGACTTTCCATCT-AGCCCTGATCAGGGCCTTATCTTCTG
Md_K224_mRNA  ----------------------------------------------------------------------------------------------------

              16301                                                                                              16400
Hs_KRT224P    ATAATTCAATGGCATCAGAATGTCACATAGAACTAAAAG-------------------------TAGTAAGTAGCACAGATGATAAGTGCCTGGGAACTC
Sh_Krt224     ATACTTG-----TATCAGAGTGTTTGTATCACATAAACACTAACTTTCATT-----TCCTTTCATTCACAGAATCAACAAAAACTTTTCTCTATAAAACC
Sh_K224_mRNA  -----------------------------------------------------------------------AATCAACAAAAACTTTTCTCTATAAAACC
Md_Krt224     ATATTTGGACTGTATCAGTGTGTTTGTATTATATAAACTATAACTTTCATTCCACTTCCTTTCATTCTCAGAACCGACAAAAACTTTTCTCTATAAAACC
Md_K224_mRNA  -----------------------------------------------------------------------AACCGACAAAAACTTTTCTCTATAAAACC

              16401                                                                   16474
Hs_KRT224P    ACAGATAATAATTCAATATGGGCCAAAGAGGCCAGTAAAAAACTCATGAAGCTGTGAATTAAAGCTCCAA-TAT
Sh_Krt224     ATTGTTGAAGAGCTAGTTGATGGAATTGTTGTCTCATCAAAAATAA--AAAATGTTCATCAGAGATCAGCCTAA
Sh_K224_mRNA  ATTGTTGAAGAGCTAGTTGATGGAATTGTTGTCTCATCAAAAATAA--AAAATGTTCATCAGAGATCAGCCTAA
Md_Krt224     ATTGTTGAAGAGCTGGTGGATGGAATTGTTGTCTCTTCAAAAATTA--AAGATGTTCATCAGAGATCAGCCTAA
Md_K224_mRNA  ATTGTTGAAGAGCTGGTGGATGGAATTGTTGTCTCTTCAAAAATTA--AAGATGTTCATCAGAGATCAGCCTAA
```

Suppl. Fig. S3. The human genome contains a *KRT224P* pseudogene. The human (*Homo sapiens*, Hs) genomic sequence of pseudogene *KRT224P* (chromosome 17, NC_000017.11, region: 40666504 – 40675035, genome assembly accession number: GCF_000001405.38) was aligned with genomic and mRNA sequence of *Krt224* of the tasmanian devil (*Sarcophilus harrisii*, Sh) (GeneID: 100922568, transcript ID: XM_012548331.1, genome assembly accession number: GCF_000189315.1) and the opossum (*Monodelphis domestica*, Md) (GeneID: 100922568, transcript_id: XM_012548331.1, genome assembly accession number: GCF_000189315.1). Red fonts indicate nucleotides that are identical in all sequences. Start and stop codons are highlighted by green shading. Mutations that introduce reading frame shifts or destroy the start or stop codon in the human sequence are indicated by red shading. Splice donor and acceptor signals at the borders of introns are indicated by blue shading.

A

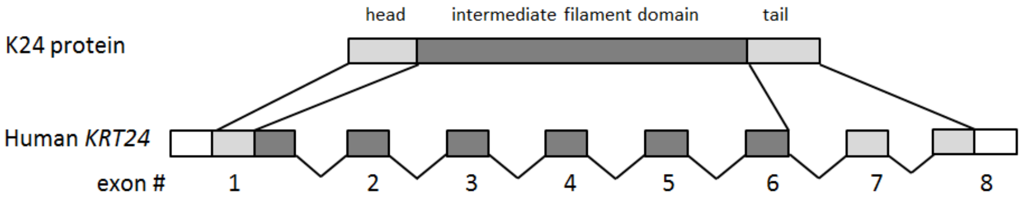

B

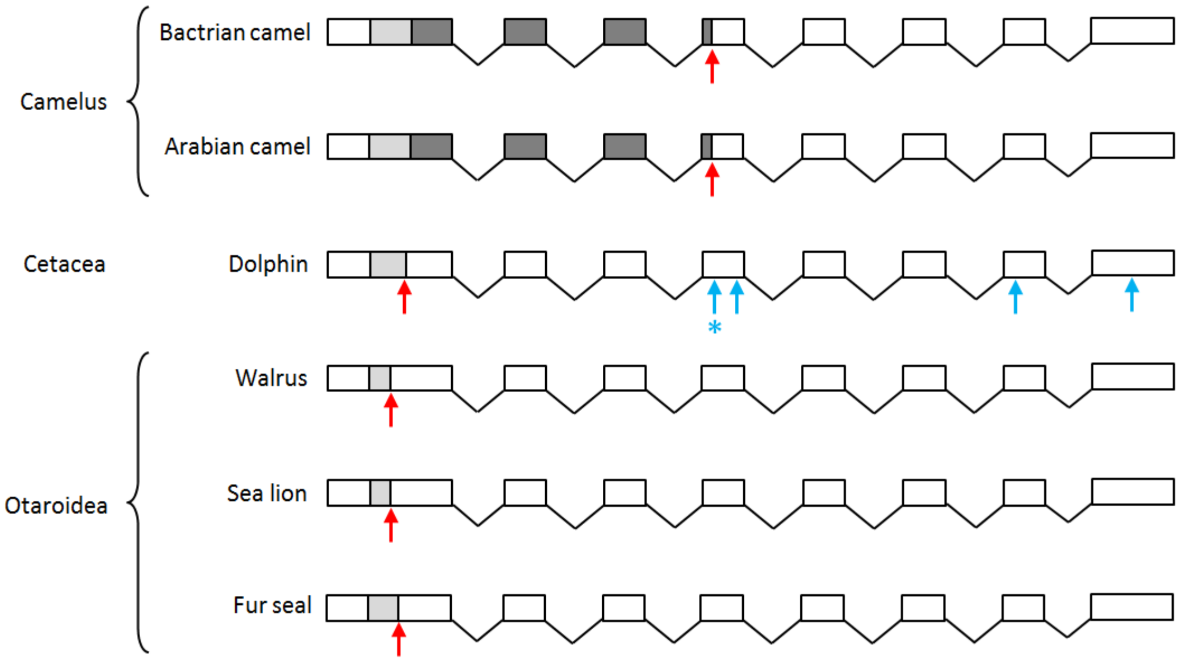

C

```
                 1                                                                                                  100
Human            MSCSSRASSSRAGGSSSARVSAGGSSFSSGSRCGLGGSSAQGFRGGASSCSLSGGSSGAFGGSFGGGFGSCSVGGGFGGASGSGTGFGGGSSFGGVSGFG
Bactrian Camel   MSCSSRVSSRTRGSSSVRVSAGGGSFSGGSSCRLRGGSARGFRGGAGSWSLSRGTSWGYGG---------------------------------GG----
Arabian Camel    MSCSSRVSSRTRGSSSVRVSAGGGSFSGGSSCRLRGGSARGFRGGAGSCGLSGGTSWGYGGSFGGG----------------------GVGSFGGG----
Alpaca           MSCSSRVSSRTRGSSAVRVSAAGGASFSGGSSCGLGRGSARGFRGGAGSCGLSGGSSWGYGGSFGGG?-----------------GLGGGSGLGGG----
Pig              MSCSTRVSSSRARGSSSVRVSAGGSSFSSGSRCGLGAGSAPGFRGGAGSCGLSGGSSSGFGGSFRGGFGSCSVAGGFGGASGSVAGYGGGSGFGGGSGFG
Cattle           MSCSSRVSSSRAGGSSSVRVSAGGSSFSSGSRIGLGGGSARGFRGGAGSYGLSGGSSGGFGG----GFGSCSVGGGFGGASGSGIGFGGGSSFGGGSSFG
Hippopotamus     MPCSSRVSSSRAGGSSSVRVSAGGSSRSSRSRCGLAGGSARGFQGGAGSCGLSGGSSGGFGGSFGGGFGSCSIAGGFGGASGSGIGFGGGSGFGGGAGFR
Dolphin          MSCSSLVSSSRA----------GGSSFSSRSRCSLGGGSARGFRGGAGSCGLSGGSSRGSGGSFGGSFGSCSIGGGLGGASGSGIGFGGGAGFRGYX--
Walrus           MSCSSCVSSSSAGSSGLIRVSAGGSSFSSGSRCGLGGGSTRGFX--------------------------------------------------------
Sea Lion         MSCSSCVSSSSAGSSGLIRVSAGGSSFSSGSRCGLGGGSTQGFX--------------------------------------------------------
Fur Seal         MSCSSCVSSSSAGSSGLIGVSAGGSSFSSGSRCGLGGGSTQGFRGGTGSCGLSGGSSSGFGGSFGGGFGSCSVGGGLGGTPGSGAGFGGSSGFGGGSGFG
Weddell Seal     MSCSSRISSSRAGSSGLIRVSAGGSSFSSGSRCGLGGGSTQGFRGGTGSCGLSGGSSSGFGGSFGGGFGSCSVGGGLGGTPGSGAGFGGGSGFGGGSGFG
Monk Seal        MSCSSRISSSRAGSSGLIRVSAGGSSFSSGSRCGLGGGSTQGFRGGTGSCGLSGGSSSGFGGSFGGGFGSCSVGGALGGTPGSGAGFGGGSGFGGGSGIG
Ferret           MSCSSRVSSSRAGSSGMVRVSAGGSSFSSGSRCGLGGGSARGFRGGTGSCGLSGGSTSGFGGSFGGGFGSCSGGGGLGGTAGSGAGFGGGSGFGGGSGFG
Panda            MSCSSRVSS-RTGGSGLVRGSAGGSSFSSGSRCGLGGGSTRGFRGGSGGCGLSGGSSSGFGGSFGGGFGSCSVGGGLGGTLGSGASFGGGSGFGGGSGFG
Dog              MSCSSRVSSSRAGGSGLARVSVGGSSFTSGSRCGVGSGSTRGFRGGTGSCGLSGVSSSGFGGSFGGGFGSCSVGGGLGGTSGSGAGFGGGSGFGGGSGFG

                 101                                                                                                200
Human            RGSGFCGSSRFSSG------------ATGGFYSY-----GGGMGGGVGDGGLFSGGEKQTMQNLNDRLANYLDKVRALEEANTDLENKIKEWYDKYGPGS
Bactrian Camel   --------------------------ASGGFYSY-----GGGLGGGIGDGGLFSGGEKQTMQNLNDRLANYLGKVRALEEANADLENKIKEWYDKFGPGS
Arabian Camel    --------------------------ASGGFYSY-----GGGLDGGIGDGGLFSGGEKQTMQNLNDRLANYLDKVRALEEANADLENKIKEWYDKFGPGS
Alpaca           --------------------------ASRGFYSY-----GGGLGGGIGDGGLFSGGEKQTMQNLNDRLANYLDKVRALEEANADLENKIKEWYDKFGPGS
Pig              GGSGFGGGSGFGGGSGFGG------GASGGFYSY-----GGGLGGGVGDGGLFSGGEKQTMQNLNDRLANYLDKVRALEEANADLEHKIKEWYDKFGPGS
Cattle           GGSSFGGGSSFGGGSGFGGSAGYAGGASGGFYSYAGG-VGGGVGGGLGDGGLFSGGEKQAMQNLNDRLANYLDKVRALEEANADLENKIKEWYEKFGPRS
Hippopotamus     EG------------------------ASGGFYSY-----GGGMGGGVGDGGLFSGGEKQTMQNLNDRLASYLDKVRALEEANTDLENKIKAWYDKFGPGS
Dolphin          ----------------------------------------------------------------------------------------------------
Walrus           ----------------------------------------------------------------------------------------------------
Sea Lion         ----------------------------------------------------------------------------------------------------
Fur Seal         GGSGIGVG------------------ATGGFYSYGGGGMGGWDGRWCWRWGAFPWRX-------------------------------------------
Weddell Seal     GGSGFGGGSGFGGGPGIG--------ASGGFYSYGGG-MGGGMGGGAGDGGLFSGGEKQTMQNLNDRLASYLDKVRALEEANTDLENKIREWYEKFGPGS
Monk Seal        --------------------------ASGGFYSY-----GGGMGGGAGDGGLFSGGEKQTMQNLNDRLASYLDKVRALEEANTDLENKIREWYEKFGPGS
Ferret           GGSGFGGGSGIGGG------------ASGGFYSCGGG-MGGGMGGGVGDGGLFSGGEKQTMQNLNDRLASYLDKVRALEEANTDLENKIREWYEKFGPGS
Panda            GGSGFGGGSGF????????------?????????????LGGGMGGGAGDGGLFSGGEKQTMQKLNDRLASYLDKVRALEEANTDLENKIRVWYEKFGPGS
Dog              GGSGFGGGSGFGGGSGFGG------GASGGFYSYGGG-MGGGMGGGIGDGGLFSGGEKQTMQNLNDRLASYLDKVRALEEANTDLENKIREWYDKFGPGS

                 201                                                                                                300
Human            GDGGSGRDYSKYYSIIEDLRNQIIAATVENAGIILHIDNARLAADDFRLKYENELCLRQSVEADINGLRKVLDDLTMTRSDLEMQIESFTEELAYLRKNH
Bactrian Camel   RDGGSGRDYSKYYPIIEDLRNQIITAMTENAGIVLQIDNARLAADDFRLKYENELHLRQTVEADTNGLRKVLDDLTTTRSGLEMQIESLTEELAYLKKNH
Arabian Camel    RDGGSGRDYSKYYPIIEDLRNQIITAMTENAGIVLQIDNARLAADDFRLKYENELHLRQTVEADTNGLRKVLDDLTTTRSGLEMQIESLTEELAYLKENH
Alpaca           RDGGSGRDYSKYYPIIEDLRNQIITAMTENAGIVLQIDNARLAADDFRLKYENELHLRQTVEVDTNGLRKVLDDLTTTRSGLEMQIESLTEELAYLKKNH
Pig              RDVGSGRDYSKYYPIIDDLRKQITAATIENAGIVLQIDNARLAADDFRLKYENELYLRQSVEADINGLRKVLDDLTMTRSDLEMQMENLVEELTYLKKNH
```

```
Cattle          GDGGAGRDYSKYYPVIEDVRTQIITATIENAGIVLQIDNARLAADDFRLKYENELHLRQTVEADINGLRKVLDDLTMTRSDLEMQIESLTEELAYLKKNH
Hippopotamus    GDGGAGRDYSKYYPIIEDLKNQIITATIENAGIILQIDNARLAADDFRLKYENELHLRQTVEADINGLRKVLDDLTMTRSDLEMQIESLTEELAYLKKNH
Dolphin         ----------------------------------------------------------------------------------------------------
Walrus          ----------------------------------------------------------------------------------------------------
Sea Lion        ----------------------------------------------------------------------------------------------------
Fur Seal        ----------------------------------------------------------------------------------------------------
Weddell Seal    GDGGSGKDYSKYYPIIEDLRNQIITATIENAGIVLQINNARLAADDFRLKYENELYLRQAVEADINGLRKVLDDLTMTRSDLEMQVESLTEELAYLKKNH
Monk Seal       GDGGSGKDYSKYYPIIEDLRNQIITATIENAGIVLQINNARLAADDFRLKYENELYLRQAVEADINGLRKVLDDLTMTRSDLEMQVESLTEELAYLKKNH
Ferret          GDGGSGKDYSKYYPVIEDLRNQIITATIENAGIVLQIDNARLAADDFRMKYENELYLRQAVEADINGLRKVLDDLTMTRSDLEMQIESLTEELAYLKKNH
Panda           GDGGSGKDYSKYYPIIEDLRGQIITATIENAGIVLQIDNARLAADDFRLKYENELYLRQAVEADINGLRKVLDDLTMTRSDLEMQIESLTEELAYLKKNH
Dog             GDGGSGKDYSKYYPIIEDLRNQIITATIENAGIVLQIDNARLAADDFRLKYENELYLRQAVEADINGLRKVLDDLTMTRSDLEMQIESLTEELAYLKKNH

                301                                                                                              400
Human           EEEMKNMQGSSGGEVTVEMNAAPGTDLTKLLNDMRAQYEELAEQNRREAEERFNKQSASLQAQISTDAGAATSAKNEITELKRTLQALEIELQSQLAMKS
Bactrian Camel  EEEMKRVQX-------------------------------------------------------------------------------------------
Arabian Camel   EEEMKRVQX-------------------------------------------------------------------------------------------
Alpaca          EEEMKRVQGGSGGDVTVEMNAAPGTDLTKLLNDMRAQYEELAEQNRREAEEQFNKQSASLQAQISTDAGAASSAKNEITELKRTLQALEIELQSQLAMKS
Pig             EEEMKSMQGSSGGDVTVEMNAAPGTDLTKLLNDMRAQYEELAEQNRREAEEQFKKQSASLQAQISTDAGAASSAKNEITELKRTLQALEIELQSQLVMKS
Cattle          EEEMKSMQGSSRGDVTVEMNAAPGTDLTKLLNDMRAQYEELAEQNRREAEEQFNKQSASLQAQISTDAGAANSAKNEITELKRTLQALEIELQSQLAMKS
Hippopotamus    EEEMKSMQGSSGGDMTVEMDAAPGTDLTKLLNDMRARYEELAEKNRQEAEEQFNKQSASLQAQICTDSGAASSAKSEITELKRTLQALEIELQSQLTMKS
Dolphin         ----------------------------------------------------------------------------------------------------
Walrus          ----------------------------------------------------------------------------------------------------
Sea Lion        ----------------------------------------------------------------------------------------------------
Fur Seal        ----------------------------------------------------------------------------------------------------
Weddell Seal    EEEMKSMQGSSRGDVTVEMNAAPGTDLTKLLNDMRAQYEELAEQNRREAEEQFNKQSASLQAQISTDAGAASSAKNEITELKRTLQALEIELQSQMAMKS
Monk Seal       EEEMKSMQGSSGGDVTVEMNAAPGTDLTKLLNDMRAQYEELAEQNRREAEEQFNKQSASLQAQISTDVGAARSAKNEITELKRTLQALEIELQSQRAMKS
Ferret          EEEMKSMQGSSGGDVTVEMNAAPGTDLTKLLNDMRAQYEELAEQNRREAEEQFNKQSASLQAQISTDAGAASSAKNEITELKRTLQALEIELQSQMAMKS
Panda           EEEMKSMQGSSGGDVTVEMNAAPGTDLTKLLNDMRAQYEELAEQNRREAEEQFNKQSASLQAQISTDAGAASSAKNEITELKRTLQALEIELQSQMAMKS
Dog             EEEMKSMQGSSGGDVTVEMNAAPGTDLTKLLNDMRARYEELAEQNRREAEEQFNKQSASLQAQISTDAGAASSAKNEITELKRTLQALEIELQSQMAMKS

                401                                                                                              500
Human           SLEGTLADTEAGYVAQLSEIQTQISALEEEICQIWGETKCQNAEYKQLLDIKTRLEVEIETYRRLLDGEGGGSSFA-----EFGGRNSGSVNMGSRDL-V
Bactrian Camel  ----------------------------------------------------------------------------------------------------
Arabian Camel   ----------------------------------------------------------------------------------------------------
Alpaca          SLEGTLADTEAGYMAQLSQIQIQISSLEEQICQIRGETECQNTEYEQLLDIKTRLEMEIETYRRLLDGEGGSSDFGGSDFRSLGSRNTGSRNMGSRDSSM
Pig             SLEGTLADTEAGYMAQLSQIQIQISSLEEQICQIRGETECQNAEYEQLLDIKTRLEMEIETYRRLLDGEGGGSGFGGCDFRNSGSRNTGSRNIGSRDSSV
Cattle          SLEGTLADTEANYMVQLSQIQMQISSLEEQICQIRGETECQNAEYEQLLDIKTRLEMEIETYRRLLDGEGGGSGFGGSDYRRSGSR-----NMGSRDSSM
Hippopotamus    SLEGTLADTEAGYMEQLSQIQMQIGGLEEQICQIRSETECQNAEYEQLLNIKTRLEMEIETYRRLLDGEAGGSGFGGFDFRNSGSRNTGSRITGSRDSSI
Dolphin         ----------------------------------------------------------------------------------------------------
Walrus          ----------------------------------------------------------------------------------------------------
Sea Lion        ----------------------------------------------------------------------------------------------------
Fur Seal        ----------------------------------------------------------------------------------------------------
Weddell Seal    SLEGTLADTEAGYMAQLSEIQMQISSLEEQICQIRGETKCQNAEYEQLLDIKTRLEMEIETYRGLLNGEGGGSGFGGSDFRNSGSRNTGSRNMGSRDLSV
Monk Seal       SLEGNLADTEAGYMAQLSEIQMQISSLEEQICQIRGETKCQNAEYEQLLDIKTRLEMEIETYRGLLNGEGGGSGFGGSDFRNSGSRNTGPRNMGSRDLSV
Ferret          SLEGTLADTEAGYMAQLSEIQMKISSLEEQICQIRGETECQNAEYEQLLNIKTRLEMEIETYRRLLDGEGGGCGSGGSDLRNSGSRSTGSRNMGS-----
Panda           SLEATLADTEGGYGAQLSEIQMQISSLEEQICQIRGETECQNAEYEQLLDIKTRLEMEIETYRRLLDGEGGGSGFGGSDCRSSGSRNTGSRNMGSRDVST
Dog             SLEATLADTEAGYMAQLSEIQMQISSLEEQICQIRGETECQNAEYEQLLDIKTRLEMEIETYRRLLDGEGCGSGFGGSDFRNSGSRNTGSRNMGSRDMSM

                501                                          548
Human           SGDSRSGSCSGQGRDSSKTRVTKTIVEELVDGKVVSSQVSSISEVKVK
Bactrian Camel  ------------------------------------------------
Arabian Camel   ------------------------------------------------
Alpaca          SGDSRSGSCSVQGRDPSKSRVTKTIIEEVVDGKVVSSQVSNVSEVKIK
Pig             SGDSRSGSCSVQGRDPSKSRVTKTIVEEVVDGKVVSSQVSNISEVKLK
Cattle          SDGSRSGT-SVQGRDPSKSRVTKTIVEEVVDGKVISSQVSNVSEVKLK
Hippopotamus    SGDSRSGSSVVQGRDPSKTRVTKTIVEEVVDGKVVSSQVSNISEVKLK
Dolphin         ------------------------------------------------
Walrus          ------------------------------------------------
Sea Lion        ------------------------------------------------
Fur Seal        ------------------------------------------------
Weddell Seal    SGDSRSGSCSGQGRDPNKTRVTKTIVEEVVDGKVISSQVSNVSEVKVK
Monk Seal       SDDSRSGSCSGQGRDPNKTRVTKTIVEEVVDGKVISSQVSNVSEVKVK
Ferret          -GDSRSGSCSGQGRDPNKTRVTKTIVEEVVDGKVISSQVSNVSEVKVK
Panda           SGDSRSGSCSGPGRDPNKTRVTKTIVEEVVDGRVISSQVSNVSEVKVK
Dog             SGESRSGSCSVQGRDPNKTRVTKTIVEEVVDGKVVSSQVSNVSEVKVK
```

Suppl. Fig. S4. Inactivating mutations of *Krt24* in distinct phylogenetic clades of mammals. (A) The structures of the K24 protein and the *KRT24* gene are schematically depicted. **(B)** Positions of premature stop codons in the *Krt24* genes of the indicated species are shown by vertical arrows. The dolphin *Krt24* gene contains several in-frame stop codons, one which (asterisk) was confirmed by PCR amplification and Sanger sequencing. **(C)** K24 amino acid sequence alignment. Amino acid sequences of K24 of the following species were obtained from GenBank whereby sequences of GenBank "low quality protein predictions" (camels, dolphins, walrus, sea lion, and fur seal) were truncated at positions corresponding to the first in-frame stop codon: *Homo sapiens* (human, accession number: NP_061889.2), *Sus scrofa* (pig, XP_020922673.1), *Bos taurus* (cattle, XP_010814563.1), *Monachus schauinslandi* (monk seal, XP_021559829.1), *Leptonychotes weddellii* (weddell seal, XP_006745203.1), *Mustela putorius furo* (ferret, XP_004764641.1), *Ailuropoda melanoleuca* (giant panda, XP_019661193.1) and *Canis lupus familiaris* (dog, XP_548129.3). The *Krt24* genes of several species were predicted differently from the current GenBank versions (March 2019) and, accordingly, the amino acid sequences at the carboxy-terminus of K24 were corrected for *Vicugna pacos* (alpaca, XP_006214348.1), *Camelus dromedarius* (Arabian camel, XP_010990151.1), *Camelus bactrianus* (Bactrian camel, XP_010958858.1), *Odobenus rosmarus* (walrus, XP_004417780.1), *Zalophus californianus* (sea lion, XP_027424281.1), *Callorhinus ursinus* (fur seal, XP_025715054.1), and *Tursiops truncatus* (dolphin, XP_004332181.2). The protein sequence of *Hippopotamus amphibius* (hippopotamus) was predicted using the Basic Local Alignment Search Tool (BLAST) on whole genome shotgun sequences (scaffold: NKPW01007369.1, coding sequence in the nucleotide range 104415-108286). The positions of premature in-frame stop codons are indicated by "X" with red shading. Question marks in the sequence of the panda (Gene ID: 100465178, genome assembly accession number: GCF_000004335.2)) and the alpaca (Gene ID: 102525652, genome assembly accession number: GCF_000164845.1) indicate unknown amino acid residues due to gaps in the genome sequences.

```
                                                        E  M  K  S  M  Q  G  S
Cattle GenBank     TTTTCATCACTATGGTCCTTTTTGCTT-CGGGCTAGGAAATGAAGAGTATGCAAGGAAGC
                   ||||||||||| |||||  |||| ||| |  ||||||||||||||||||||||| |||||
Dolphin GenBank    TTTTCATCACTCTGGTCTATTTTCCTTTCATGCTAGGAAATGAAGAGTATGCAACGAAGC
                   ||||||||||||||||||||||||||||||||||||||||||||||||||||||||||||
Dolphin PCR        TTTTCATCACTCTGGTCTATTTTCCTTTCATGCTAGGAAATGAAGAGTATGCAACGAAGC
                                                        E  M  K  S  M  Q  R  S


                    S  R  G  D  V  T  V  E  M  N  A  A  P  G  T  D  L  T  K  L
Cattle GenBank     TCCAGAGGGGACGTGACTGTGGAAATGAACGCTGCCCCGGGTACAGACCTGACTAAGTTA
                   ||| ||||| | ||||| || ||| |||||      || || || |||||    ||||||
Dolphin GenBank    TCCGGAGGG-ATGTGACCGTAGAACTGAAC------CCAGGGACGGACCT----AAGTTA
                   ||||||||| ||||||||||||||||||||      ||||||||||||||    ||||||
Dolphin PCR        TCCGGAGGG-ATGTGACCGTAGAACTGAAC------CCAGGGACGGACCT----AAGTTA
                    S  G  G   M  *  -  -  -  -  -        -  -  -  -      -  -
                                premature stop


                    L  N  D  M  R  A  Q  Y  E  E  L  A  E  Q  N  R  R  E  A  E
Cattle GenBank     CTGAATGACATGAGGGCACAGTATGAGGAGCTGGCTGAGCAGAACCGCCGCGAGGCCGAG
                   ||||||||| ||||||||||||||||||| |||||||||||||||| | ||||||| ||
Dolphin GenBank    CTGAATGACGTGAGGGCACAGTATGAGGACCTGGCTGAGCAGAACCACTGCGAGGCTGAA
                   ||||||||||||||||||||||||||||||||||||||||||||||||||||||||||||
Dolphin PCR        CTGAATGACGTGAGGGCACAGTATGAGGACCTGGCTGAGCAGAACCACTGCGAGGCTGAA
                   -  -  -  -  -  -  -  -  -  -  -  -  -  -  -  -  -  -  -  -

                    E  Q  F  N  K  Q
Cattle GenBank     GAGCAGTTCAACAAGCAGGTAGATCATCACCCTGAGCCTCCAAGTCCATGGCCACATGTA
                   |||||||||||| |||||||||||||||||||||||||||| || || || || ||   |
Dolphin GenBank    GAGCAGTTCAACGAGCAGGTAGATCATCACCCTGAGCCTCCGAGCCC-TGCCCGCAGCCA
                   ||||||||||||||||||||||||||||||||||||||||||||||| ||||||||||||
Dolphin PCR        GAGCAGTTCAACGAGCAGGTAGATCATCACCCTGAGCCTCCGAGCCC-TGCCCGCAGCCA
                   -  -  -  -  -  -
```

Suppl. Fig. S5. Confirmation of one of the mutations that disrupt the open reading frame of *Krt24* in the bottlenose dolphin. Nucleotide sequences of *Krt24* genes of *Bos taurus* (Cattle, GeneID: 788424, genome assembly accession number: GCF_002263795.1) and *Tursiops truncatus* (dolphin, GeneID: 101318927, genome assembly accession number: GCF_001922835.1) were downloaded from GenBank. A segment (corresponding to exon 4 and neighboring introns) of the *Krt24* gene of the dolphin was amplified by PCR and sequenced as described in the *Material and methods* section. The nucleotide sequences were aligned and amino acid sequences obtained by translation of the coding sequences are shown above the alignments. The theoretical translation of the exonic nucleotide sequence of the dolphin is shown below the sequence. Note that this exon is preced by at least one disruptive mutation in an upstream exon (see Suppl. Fig. S4). Blue fonts indicate a frame-shift and a premature stop codon. Intronic splice donor and acceptor signals are indicated by grey shading.

```
                               E  M  K  R  V  Q  G  G  S  G  G  D  V  T  V  E  M  N  A  A  P  G  T  D  L  T
Alpaca GB        CCCTCCTTGCTAGGAAATGAAGCGTGTGCAAGGAGGCTCCGGAGGGGATGTGACCGTAGAAATGAATGCTGCCCCAGGAACAGACCTGACT
Arab. camel GB   CCCTCCTTGCTAGGAAATGAAGCGTGTGCAATGAGGCTGCGGAGGGGATGTGACGGTAGAAATGAATGCTGCCCCAGGAACAGACCTGACT
Arab. camel PCR  CCCTCCTTGCTAGGAAATGAAGCGTGTGCAATGAGGCTGCGGAGGGGATGTGACGGTAGAAATGAATGCTGCCCCAGGAACAGACCTGACT
Bact. camel GB   CCCTCCTTGCTAGGAAATGAAGCGTGTGCAATGAGGCTCCGGAGGGGATGTGACGGTAGAAATGAATGCTGCCCCAGGAACAGACCTGACT
Bact. camel PCR  CCCTCCTTGCTAGGAAATGAAGCGTGTGCAATGAGGCTCCGGAGGGGATGTGACGGTAGAAATGAATGCTGCCCCAGGAACAGACCTGACT
                               E  M  K  R  V  Q  *  -  -  -  -  -  -  -  -  -  -  -  -  -  -  -  -  -  -  -
                                              premature stop

                  K  L  L  N  D  M  R  A  Q  Y  E  E  L  A  E  Q  N  R  R  E  A  E  E  Q  F  N  K  Q
Alpaca GB        AAGTTACTGAATGACATGAGGGCACAGTATGAGGAGCTGGCTGAGCAGAACCGCCGGGAGGCCGAGGAGCAGTTCAATAAGCAGGTAGA
Arab. camel GB   AAGTTACTGAATGACATGAGGGCACAGTATGAGGAGCTGGCTGAGCAGAACCGCCGGGAGGCCGAGGAGCAGTTCAATAAGCAGGTAGA
Arab. camel PCR  AAGTTACTGAATGACATGAGGGCACAGTATGAGGAGCTGGCTGAGCAGAACCGCCGGGAGGCCGAGGAGCAGTTCAATAAGCAGGTAGA
Bact. camel GB   AAGTTACTGAATGACATGAGGGCACAGTATGAGGAGCTGGCTGAGCAGAACCGCCGGGAGGCCGAGGAGCAGTTCAATAAGCAGGTAGA
Bact. camel PCR  AAGTTACTGAATGACATGAGGGCACAGTATGAGGAGCTGGCTGAGCAGAACCGCCGGGAGGCCGAGGAGCAGTTCAATAAGCAGGTAGA
                  -  -  -  -  -  -  -  -  -  -  -  -  -  -  -  -  -  -  -  -  -  -  -  -  -  -  -  -
```

Suppl. Fig. S6. *Krt24* is inactivated by a premature stop codon in camels. Nucleotide sequences of *Krt24* genes of *Vicugna pacos* (Alpaca, GeneID: 102525652, genome assembly accession: GCF_000164845.1), *Camelus bactrianus* (Bact. camel, GeneID: 105073329, genome assembly accession: GCF_000767855.1) and *Camelus dromedarius* (Arab. camel, GeneID: 105099065, genome assembly accession: GCF_000767585.1) were downloaded from GenBank (GB). A segment of the *Krt24* gene of *C. bactrianus* and *C. dromedarius* was amplified by PCR and sequenced as described in the *Material and methods* section. The nucleotide sequences were aligned and amino acid sequences obtained by translation of the coding sequences are shown above the alignments. Red fonts indicate premature stop codons. Intronic splice donor and acceptor signals are indicated by grey shading.
